# Supplementary material for: Transaminase-catalysis to produce trans-4-substituted cyclohexane-1-amines including a key intermediate towards cariprazine
Source: Commun Chem. 2024 Apr 18;7:86. doi: 10.1038/s42004-024-01148-9 (PMC11026398; doi:10.1038/s42004-024-01148-9)
Supplement: Supplementary file 2 — Supplementary information [file 42004_2024_1148_MOESM2_ESM.pdf]

Supporting information

Transaminase-catalysis to produce *trans*-4-substituted cyclohexylamines including a key intermediate towards cariprazine

Emese Farkas,[1,2*] Péter Sátorhelyi,[3] Zoltán Szakács,[2] Miklós Dékány,[3] Dorottya Vaskó,[1] Gábor Hornyánszky[1], László Poppe,[1,4*] János Éles,[2*]

[1] Department of Organic Chemistry and Technology, Budapest University of Technology and Economics, Műegyetem rkp. 3. H-1111, Budapest, Hungary.

[2] Gedeon Richter Plc., PO Box 27, H-1475, Budapest, Hungary.

[3] Fermentia Microbiological Ltd., Berlini út 47-49, H-1405. Budapest, Hungary

[4] Biocatalysis and Biotransformation Research Centre, Faculty of Chemistry and Chemical Engineering, Babeș-Bolyai University of Cluj-Napoca, Arany János str. 11. RO-400028, Cluj-Napoca, Romania.

Corresponding authors:
Emese Farkas (farkaseme@richter.hu); Prof. László Poppe (laszlo.poppe@vbk.bme.hu); Dr. János Éles (j.eles@richter.hu)

Table of Contents

1 Supplementary Methods 3

1.1 Supplementary Methods - Materials 3

1.1.1 Chemicals and solvents 3

1.1.2 Enzyme-coding plasmids 3

1.2 Supplementary Methods - Analytical methods 3

1.2.1 Thin layer chromatography 3

1.2.2 Infrared spectroscopy 3

1.2.3 Gas chromatography 3

1.2.4 Mass spectroscopy 4

1.2.5 Nuclear magnetic resonance spectroscopy 4

1.3 Supplementary Methods - Experimental 4

1.3.1 Preparation and characterization of materials 4

1.3.1.1 Synthesis of ketones **2a-c**[S13] 4

1.3.1.2 Synthesis of diastereomeric mixtures of ammonium hydrochloride salts (*cis/trans*-**1a-d**·HCl)[S16] 6

1.3.1.3 GC data and chromatograms of investigated compounds 9

1.3.2 Immobilization of recombinant E. coli whole-cells overexpressing TAs together with hollow silica microspheres as support by entrapment in a sol-gel system 11

1.3.3 Expression, purification and covalent immobilization of CvS-TA$_{W60C}$ on bisepoxide activated polymer support 11

1.3.4 Exploring the reaction parameters (temperature, cosolvents) for biotransformations with imm-CvS-TA$_{W60C}$ using the kinetic resolution of *rac*-**8** as test reaction 12

1.3.4.1 Test reaction for exploring the reaction conditions 12

1.3.4.2 GC analysis of the kinetic resolution from *rac*-**8** 13

1.3.4.3 Screening conditions for biotransformations with imm-CvS-TA$_{W60C}$ in batch mode 13

1.3.4.4 Screening conditions of biotransformations with imm-CvS-TAW60C in continuous-flow mode 16

1.3.4.4.1 Equipment for continuous-flow processes 16

1.3.4.4.2 Residence time 17

1.3.4.4.3 Optimization of reaction parameters for kinetic resolution of *rac*-**8** in continuous-flow mode 18

1.3.5 Deamination of cis/trans-1a-d with imm-CvS-TA$_{W60C}$ in continuous-flow mode 19

1.3.5.1 Investigation of the deamination of *cis/trans*-**1a-d** in continuous-flow mode with systems comprising different number of serially coupled imm-CvS-TA$_{W60C}$ columns 19

1.3.5.2 Production of pure *trans*-**1a-d** by deamination of *cis/trans*-**1a-d** with immobilized CvS-TA$_{W60C}$ in continuous-flow mode 19

Supplementary references 20

1 Supplementary Methods

1.1 Supplementary Methods - Materials

1.1.1 Chemicals and solvents

Except otherwise not stated, all solvents and chemicals were purchased from the following commercial suppliers: Sigma Aldrich (Saint Louis, MO, USA), Alfa Aesar Europe (Karlsruhe, Germany), Merck (Darmstadt, Germany), Fluka (Milwaukee, WI, USA) and used without further purification. MAT540 (MATSPHERE™ SERIES 540 - hollow silica microspheres etched with aminoalkyl and vinyl functions, with an average particle diameter of 10 µm) was obtained from Materium Innovations (Granby, QC, Canada). Ethyleneamine-functionalized methacrylic polymer resins (ReliZyme™ EA403/S; polymethyl methacrylate supports, particle size 150-300 µm, pore size 400-600 Å) and epoxide-functionalized methacrylic polymer resins (ReliZyme™ EP403/S; polymethyl methacrylate supports, particle size 150-300 µm, pore size 400-600 Å) from Resindion S.r.L. (Binasco, Italy).

1.1.2 Enzyme-coding plasmids

Original data on cloning, sequence and 3D-structure of the TAs in this work: *Arthrobacter citreus* mutant CNB05-01[S1] (*ArS*-TA)[S2] [Seq. ID 16 in Ref. S1]; from *Arthrobacter* sp. KNK168 (*ArR*-TA)[S3] [Uniprot code: F7J696, PDB code: 3WWH[S4]]; mutated variant of *Arthrobacter* sp. KNK168 (*ArR*-TA_{mut11})[S5] [PDB code: 3WWJ[S4] and 5FR9[S6]], *Aspergillus terreus* (*AtR*-TA)[S7] [Uniprot code: Q0C8G1, PDB code: 4CE5[S8]], *Chromobacterium violaceum* (*CvS*-TA_{W60C})[S9,S10] [Uniprot code: A0A1R0MXM9, PDB code: 6SNU], and *Vibrio fluvialis* (*VfS*-TA)[S11] [Uniprot code: F2XBU9, PDB codes: 3NUI, 4E3Q[S12]].

Plasmids encoding transaminase from *Arthrobacter citreus* (*ArS*-TA), from *Vibrio fluvialis* (*VfS*-TA), from *Aspergillus terreus* (*AtR*-TA), from *Arthrobacter* sp. (*ArR*-TA) and its mutated variant (*ArR*-TA_{mut11}) were a kind gift of Prof. Wolfgang Kroutil (University of Graz, Austria). The plasmid encoding the W60C mutant of ω-transaminase from *Chromobacterium violaceum* (*CvS*-TA_{W60C}) was a kind gift from Prof. Per Berglund (KTH Royal Institute of Technology, Sweden).

1.2 Supplementary Methods - Analytical methods

1.2.1 Thin layer chromatography

TLC was carried out using Kieselgel 60 F254 (Merck) sheets. Spots were visualized under UV light (Vilber Lourmat VL-6.LC, 254 nm) or after treatment with 5% ethanolic phosphomolybdic acid solution or 3% isopropanol ninhydrin solution and heating of the dried plates.

1.2.2 Infrared spectroscopy

Infrared spectra were recorded on a Bruker ALPHA FT-IR spectrometer using Opus software (Brucker) and wavenumbers of bands are listed in cm^{-1}. The IR spectra of all examined products are available in the Supplementary Data 3.

1.2.3 Gas chromatography

The reaction mixtures from amination and deamination were analyzed on GC equipped with flame ionization detector (FID) with the following column: an Agilent 5890 GC equipped with an achiral, non-polar HP-5 column (Agilent J&W; 30 m × 0.25 mm × 0.25 µm film thickness of (5%-Phenyl)-methylpolysiloxane), or an Agilent 4890 GC equipped with a chiral Hydrodex β-6 TBDM column (Macherey-Nagel; 25 m × 0.25 mm × 0.25 µm film thickness of heptakis-(2,3-di-*O*-methyl-6-*O*-t-butyl-dimethylsilyl)-β-cyclodextrin). Operation conditions: FID (250 °C), injector (250 °C), carrier gas H_2 (head pressure 12 psi, split ratio: 1:50). GC chromatograms for TA-catalyzed aminations and deaminations are presented in the Supplementary Data 2. Data were collected and analyzed using ChemStation software (Agilent).

1.2.4 Mass spectroscopy

HRMS and MS-MS analyses were performed on a Thermo Velos Pro Orbitrap Elite (Thermo Fisher Scientific) system. The ionization method was ESI operated in positive ion mode. The protonated molecular ion peaks were fragmented by CID at a normalized collision energy of 35%. For the CID experiment helium was used as the collision gas. The samples were dissolved in methanol. Data acquisition and analysis were accomplished with Xcalibur software version 2.0 (Thermo Fisher Scientific).

1.2.5 Nuclear magnetic resonance spectroscopy

All NMR samples were dissolved in DMSO-d_6 solvent and the spectra were acquired in standard 5-mm tubes at 25 °C on either of the following Avance III HDX spectrometers from Bruker BioSpin GmbH, Rheinstetten, Germany (proton frequencies are given): 400 MHz (with ^{1}H-^{19}F/^{15}N-^{31}P Prodigy CryoProbe and a SampleCase sample changer), 500 MHz (500 S2 ^{1}H/^{13}C/^{15}N TCI Extended Temperature CryoProbe) or 800 MHz (800 SA ^{1}H&^{19}F/^{13}C/^{15}N TCI CryoProbe). All probeheads were equipped with pulsed-field Z gradient units. Standard pulse programs included in the TopSpin software (version 3.5) were used. One-dimensional ^{1}H and DEPTQ spectra were recorded for all compounds. ^{1}H-^{1}H connectivities through bonds were established by 2D COSY or TOCSY (with a 15 ms Malcolm-Levitt spinlock) spectra. A ^{1}H,^{13}C HMBC spectrum optimized for $^{n}J_{C,H}$ = 8 Hz couplings and/or a multiplicity-edited ^{1}H,^{13}C HSQC spectrum were recorded to complete ^{13}C signal assignment and aid structure verification. Axial or equatorial positions of protons on the cyclohexane ring with chair conformation were determined by interpreting the vicinal coupling constants using a Karplus-type relationship. In the case of overlapping multiplets of stereoisomers, the individual spin systems visualized separately by selective 1D TOCSY spectra (90 ms mixing time and an RSNOB soft pulse for selective inversion). Spatial relationships were also explored by observing through-space proton-proton connectivities in 2D NOESY (with 500 ms mixing time) or ROESY (with 200 ms mixing time) spectra for some compounds. NMR spectra of all examined products are available in the Supplementary Data 1.

1.3 Supplementary Methods - Experimental

1.3.1 Preparation and characterization of materials

1.3.1.1 Synthesis of ketones **2a-c**[S13]

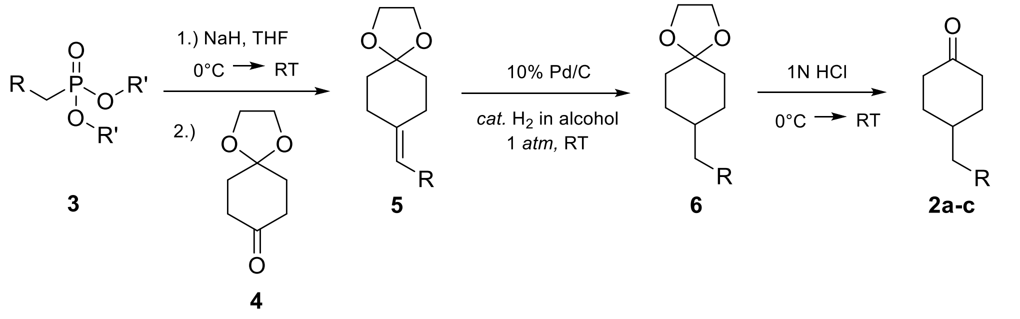

Figure S1 Synthesis of **2a-c** (Horner-Wadsworth-Emmons reaction, hydrogenation followed by deprotecting) performed on multigram scale

To a dry round bottom flask was added the solution of the previously hexane-washed sodium hydride (2 eq.) in dry tetrahydrofuran and cooled to (-5)-0 °C. Holding the temperature at 0-5 °C the solution of corresponding phosphonate (1.2 eq.) in dry tetrahydrofuran was added dropwise, and the resulted mixture was stirred at 0 C for 0.5 h and at room temperature for an hour. After it was cooled again to (-5)-0 °C and the solution of 1,4-cyclohexanedione mono ethylene ketal (1 eq.) in dry THF was dropped, and the resulting mixture was stirred at 0°C for an hour then at room temperature overnight. The THF was

evaporated from the reaction mixture and the residue was diluted with brine (60 ml) and the aqueous phase was extracted with ethyl acetate (3·80 ml). The unified organic phases were extracted saturated brine (80 ml) and dried over Na_2SO_4 and concentrated in vacuum to yield the crude product (eluent: Hex:EtOAc=2:1).

Without further purification the unsaturated crude products were hydrogenated. They (1 eq.) were dissolved in the corresponding alcohol and treated with 10% Pd/C (10w/w%) under 1 atm of hydrogen until the hydrogenation was complete (followed by TLC, eluent: Hex:EtOAc=2:1). After completion of the reaction the mixture was filtered through Celite® and the solvent was removed by vacuum rotary evaporation to yield the saturated products.

The final step was the removal of protecting group. The saturated products (1 eq.) were dissolved in the corresponding alcohol and cooled to 0 °C. 1N HCl (3 eq.) solution was added dropwise and stirred at 0 °C for an hour than at RT overnight. After the reaction was complete, it was cooled to 0 °C and the pH was adjusted to pH 7 by 1N NaOH. The mixture was extracted with ethyl acetate (3·80 ml) and the unified organic phases were extracted with saturated brine and dried over Na_2SO_4. and concentrated in vacuum. The crude products **2a-c** were purified by silica gel column chromatography (eluent: hexane-EtOAc=4:1) to give rise to the ketone substrates.

Ethyl-2-(4-oxocyclohexyl)acetate (**2a**)

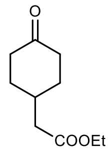

2a

Reaction of the solution of ethyl-2-(diethoxyphosphoryl)acetate (18.3 mL, 20.7 g, 92.2 mmol) in dry THF (50 ml) and washed by hexane from oil NaH (3.69 g, 154 mmol) in dry THF (40 ml) with 1,4-cyclohexanedione mono ethylene ketal (12.00 g, 76.84 mmol) in dry THF (50 ml) afforded ethyl-2-(1,4-dioxaspiro[4,5]decan-8-ylidene)acetate (16.81 g, 97 % crude yield) as colorless liquid.[S13]

The reaction of ethyl-2-(1,4-dioxaspiro[4,5]decan-8-ylidene)acetate (16.65 g, 71.00 mmol) and 10% Pd/C (1.67 g) in ethanol (70 ml) under atmospheric hydrogen afforded ethyl-2-(1,4-dioxaspiro[4,5]decan-8-yl)acetate (16.48 g, 98% crude yield) as colorless oil.[S13]

Reaction of the solution of ethyl-2-(1,4-dioxaspiro[4,5]decan-8-yl)acetate (15.00 g, 65.70 mmol) in ethanol (150 ml) and 1 N HCl (150 ml) afforded ethyl-2-(4-oxocyclohexyl)acetate **2a** (5.32 g, 42 % purified yield) as a colorless oil.[S14]

^{1}H NMR (500 MHz, DMSO-d_6) δ_H: 4.07 (2H, q, J=7.1 Hz, OCH_2-CH_3), 2.39 (2H, td, J=13.7 Hz, J=5.9 Hz, 2×CH_{ax}), 2.31 (2H, d, J=7.1 Hz, CH_2-COOEt), 2.21-2.15 (2H+1H, m, 2×CH_{eq}+CH_{ax}-CH_2COOEt), 1.98-1.92 (2H, m, 2×CH_{eq}), 1.40 (2H, qd, J=12.1 Hz, J=4.3 Hz, 2×CH_{ax}), 1.19 (3H, t, J=7.1 Hz, CH_3-CH_2);

^{13}C NMR (125 MHz, DMSO-d_6) δ_C: 210.4 (*C*O), 171.9 (CH_2-*C*OOEt), 59.7 (O*C*H_2-CH_3), 39.8 (2x*C*H_2), 39.3 (*C*H_2), 32.3 (*C*H-CH_2COOEt), 31.5 (2x*C*H_2), 14.0 (*C*H_3).

HRMS (*m/z*): [M$^+$] calcd. for $C_{10}H_{17}O_3$, 185.11722; found 185.11727.

IR (liquid film) ν_{max}: 2933, 1710, 1449, 1368, 1345, 1278, 1201, 1150, 1094, 1029, 968, 754, 503 cm^{-1}.

Isopropyl-2-(4-oxocyclohexyl)acetate (**2b**)

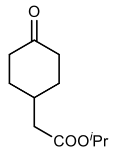

2b

Reaction of isopropyl-2-(diisopropoxyphosphoryl)acetate (25.00 g, 93.3 mmol) and washed by hexane from oil NaH (3.75 g, 156.4 mmol) in dry THF (100 ml) with 1,4-cyclohexanedione mono ethylene ketal (12.2 g, 78.2 mmol) in dry THF (50 ml) afforded isopropyl-2-(1,4-dioxaspiro[4,5]decan-8-ylidene)acetate (17.12 g, 91 % crude yield) as colorless liquid.

The reaction of isopropyl-2-(1,4-dioxaspiro[4,5]decan-8-ylidene)acetate (15.00 g, 62.46 mmol) and 10% Pd/C (1.5 g) in isopropanol (220 ml) under atmospheric hydrogen afforded isopropyl-2-(1,4-dioxaspiro[4,5]decan-8-yl)acetate (14.63 g, 97 % crude yield) as colorless liquid.

Reaction of the solution of isopropyl-2-(1,4-dioxaspiro[4,5]decan-8-yl)acetate (14.00 g, 57.85 mmol) in isopropanol (170 ml) and 1 N HCl (170 ml) afforded ethyl-2-(4-oxocyclohexyl)acetate **2b** (8.47 g, 74 % purified yield) as a colorless oil.

^{1}H NMR (500 MHz, DMSO-d_6) δ_H: 4.91 (1H, quint, J=6.3 Hz, CH-(CH$_3$)$_2$), 2.39 (2H, td, J=13.8 Hz, J=6.0 Hz, 2×CH_{ax}), 2.28-2.27 (2H, m, CH_2), 2.19-2.14 (1H+2H, m, CH_{ax}-CH$_2$COO^{i}Pr, 2×CH_{eq}), 1.96-1.92 (2H, m, 2×CH_{eq}), 1.40 (2H, qd, J=13.0 Hz, J=4.1 Hz, 2×CH_{ax}), 1.19 (6H, d, J=6.3 Hz, 2×CH_3);
^{13}C NMR (125 MHz, DMSO-d_6) δ_C: 210.4 (CO), 171.4 (COO^{i}Pr), 66.9 (CH-(CH$_3$)$_2$), 39.6 (CH$_2$-COO^{i}Pr), 32.3 (CH-CH$_2$COO^{i}Pr)+2xCH$_2$), 31.4 (2xCH$_2$), 21.5 (CH$_3$).
HRMS: (m/z): [M$^+$] calcd. for $C_{11}H_{19}O_3$, 199.13287; found 199.13276.
IR (liquid film) ν_{max}: 2979, 1711, 1449, 1374, 1278, 1203, 1161, 1107, 967 cm^{-1}.

4-Benzylcyclohexyl-1-one (**2c**)

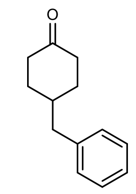
2c

Reaction of diethyl-(2-oxo-4-phenylbuthyl)phosphonate (16.0 ml, 17.50 g, 76.83 mmol) and washed by hexane from oil NaH (3.07 g, 128.04 mmol) in dry THF (40 ml) with 1,4-cyclohexanedione mono ethylene ketal (10.00 g, 64.02 mmol) in dry THF (80) afforded 8-benzylidene-1,4-dioxaspiro[4,5]decane (13.92 g, 94 % crude yield) as yellow liquid.
The reaction of 8-benzylidene-1,4-dioxaspiro[4,5]decane (13.60 g, 59.05 mmol) and 10% Pd/C (1.36 g) in methanol (80 ml) under atmospheric hydrogen afforded 8-benzyl-1,4-dioxaspiro[4,5]decane (13.47 g, 98 % crude yield) as yellow liquid.
Reaction of the solution of 8-benzyl-1,4-dioxaspiro[4,5]decane (13.00 g, 55.96 mmol) in methanol (168 ml) and 1 N HCl (168 ml) afforded 4-benzylcyclohexyl-1-one **2c** (8.54 g, 81% purified yield) as white solid.[S15]
^{1}H NMR (500 MHz, DMSO-d_6) δ_H: 7.31-7.28 (2H, m, ArH), 7.21-7.17 (3H, m, ArH), 2.57 (2H, d, J=7.2 Hz, CH_2Ph), 2.33 (2H, td, J=13.8 Hz, J=5.9 Hz, 2×CH_{ax}-CO), 2.19-2.15 (2H, m, 2×CH_{eq}-CO), 2.04-1.96 (1H, m, CH_{ax}-CH$_2$Ph), 1.90-1.85 (2H, m, 2×CH_{eq}-CHCH$_2$Ph), 1.36 (2H, qd, J=13.3 Hz, J=4.6 Hz, 2×CH_{ax});
^{13}C NMR (125 MHz, DMSO-d_6) δ_C: 210.9 (C=O), 140.4 (ArC), 128.8 (ArCH$_{orto}$); 128.1 (ArCH$_{meta}$), 125.8 (ArCH$_{para}$), 41.1 (CH$_2$Ph), 39.9 (2xCH$_2$CO), 36.9 (CHCH$_2$Ph), 31.6 (2xCH$_2$CH).
HRMS: (m/z): [M$^+$] calcd. for $C_{13}H_{17}O$, 189.12739; found 189.12733.
IR (liquid film) ν_{max}: 3026, 2933, 2852, 1719, 1602, 1494, 1457, 1420, 1333, 1297, 1159, 962, 763, 704 cm^{-1}.

1.3.1.2 Synthesis of diastereomeric mixtures of ammonium hydrochloride salts (*cis/trans*-**1a-d**·HCl)[S16]

To a round-bottom flask was added the solution of substrate ketone (**2a-d**, 7.97-44.6 mmol, 1 eq.), 10% Pd/C (10 w/w%) and ammonium formate (6 eq.) in the corresponding alcohol (30-100 ml) and stirred at 30-40°C. After completion of the reaction the mixture was filtered through Celite® and the solvent was removed by vacuum rotary evaporation. The pH of the residue was adjusted to 1 by aqueous cc. HCl, and the remaining ketone was removed by extraction with dichloromethane (3×40 mL). After removal of the ketone, pH of the aqueous phase was adjusted to 10 by addition of ammonium hydroxide (25%) and the residual amine was extracted with dichloromethane (3×40 mL). The unified organic phase (after pH adjustment) was extracted with saturate brine (30 mL) and dried over Na_2SO_4 and concentrated in vacuum to yield the product amine. The amine product (**1a-d**·HCl) was dissolved in diethyl ether before introducing HCl-gas. The salt was then isolated by filtration and dried to give amine as its hydrochloride salt (**1a-d·HCl**).

cis/trans-4-(2-Ethoxy-2-oxoethyl)cyclohexan-1-aminium chloride (cis/trans-**1a**·HCl)

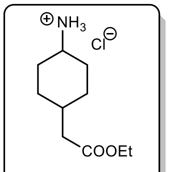

cis/trans-**1a**·HCl

Reaction of ethyl-2-(4-oxocyclohexyl)acetate **2a** (1.50 g, 8.14 mmol) and 10% Pd/C (0.15 g) with ammonium formate (3.08 g, 48.8 mmol) in ethanol (40 ml) afforded *cis/trans*-4-(2-ethoxy-2-oxoethyl)cyclohexane-1-amine *cis/trans*-**1a** (1.24 g, 83 %yield) as colorless liquid. Lastly after the introducing of HCl-gas the *cis/trans* diastereomeric mixture of 4-(2-ethoxy-2-oxoethyl)cyclohexan-1-aminium chloride *cis/trans*-**1a**·HCl (1.30 g, 72 % yield) was formed as white solid.

HRMS: (*m/z*): [M^+] calcd. for $C_{10}H_{20}O_2N$, 186.14886; found 186.14853.

IR (liquid film) ν_{max}: 2933, 2552, 2037, 1731, 1604, 1509, 1451, 1370, 1291, 1177, 1033 cm^{-1}.

cis:trans=2.30:1.00 (^{1}H-NMR)

cis: **^{1}H NMR** (500 MHz, DMSO-*d₆*) δ_H: 8.14 (3H, br, NH_3^+), 4.09-4.02 (2H, m, OCH_2), 3.18-3.09 (1H, m, CH_{ax}-NH_3^+), 2.27 (2H, d, *J*=7.5 Hz, CH_2COOEt), 1.98-1.86 (1H, m, CH_{eq}-CH_2COOEt), 1.69-1.62 (4H, m, 4×CH), 1.53-1.43 (4H, m, 4×CH), 1.18 (3H, t, *J*=7.2 Hz, CH_3);

^{13}C NMR (125 MHz, DMSO-d_6) δ_C: 171.96 (CO), 59.62 (OCH_2), 47.2 (*C*H-NH_3^+), 37.92 (CH_2COOEt), 30.58 (CH_{ax}-CH_2COOEt), 25.96 (2xCH_2CH), 25.89 (2xCH_2CHN$H_3^+$$Cl^-$), 14.04 ($CH_3$).

trans: **^{1}H NMR** (500 MHz, DMSO-d_6) δ_H: 8.14 (3H, br, NH_3^+), 4.09-4.02 (2H, m, OCH_2), 2.94-2.82 (1H, m, CH_{ax}-NH_3^+), 2.18 (2H, d, *J*=7.6 Hz, CH_2COOEt), 1.98-1.86 (2H, m, 2×CH_{eq}), 1.72 (2H, br d, *J*=14.0 Hz, CH_{eq}CHNH_3^+),1.64-1.55 (1H, m, CH_{ax}-CH_2COOEt), 1.34 (2H, qd, *J*=12.4 Hz, *J*=3.1 Hz, 2×CH_{ax}), 1.18 (3H, t, *J*=7.2 Hz, CH_3), 1.03 (2H, qd, *J*=12.7 Hz, *J*=3.5 Hz, 2×CH_{ax});

^{13}C NMR (125 MHz, DMSO-d_6) δ_C: 171.8 (CO), 59.6 (OCH_2), 48.9 (*C*H-NH_3^+), 40.3 (CH_2COOEt), 33.1 (CH_{ax}-CH_2COOEt), 29.8 (2xCH_2), 29.75 (2xCH_2), 14.0 (CH_3).

cis/trans-4-(2-Isopropoxy-2-oxoethyl)cyclohexan-1-aminium chloride (*cis/trans*-**1b**·HCl)

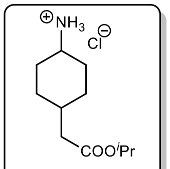

cis/trans-**1b**·HCl

Reaction of isopropyl-2-(4-oxocyclohexyl)acetate **2b** (2.00 g, 10.1 mmol) and 10% Pd/C (0.20 g) with ammonium formate (3.82 g, 60.5 mmol) in isopropanol (40 ml) afforded the *cis/trans*-4-(2-ethoxy-2-oxoethyl)cyclohexane-1-amine *cis/trans*-**1b** (1.71 g, 85 % yield) as colorless liquid. Lastly after the introducing of HCl-gas the *cis/trans* diastereomeric mixture of 4-(2-ethoxy-2-oxoethyl)cyclohexan-1-aminium chloride *cis/trans*-**1b**·HCl (1.75 g, 73 % yield) was formed as white solid.

HRMS: (*m/z*): [M^+] calcd. for $C_{11}H_{22}O_2N$, 200.16451; found 200.16423.

IR (liquid film) ν_{max}: 2944, 2627, 2553, 2056, 1729, 1607, 1510, 1458, 1391, 1297, 1182, 1107 cm^{-1}.

cis:trans=1.07:1.00 (^{1}H-NMR)

cis: **^{1}H NMR** (500 MHz, DMSO-d_6) δ_H: 8.10 (3H, br, NH_3^+), 4.89 (1H, quint, *J*=6.25 Hz, C*H*-(CH_3)$_2$), 3.13 (1H, quint, *J*=5.6 Hz, CH_{eq}-NH_3^+), 2.21 (2H, d, *J*=7.55 Hz, CH_2-COO^{i}Pr), 1.95-1.89 (1H, m, CH_{ax}-CH_2COO^{i}Pr), 1.67-1.64 (4H, m, 4×C*H*), 1.53-1.43 (4H, m, 4×C*H*), 1.18 (6H, d, *J*=1.71 Hz, 2×CH_3);

^{13}C NMR (125 MHz, DMSO-d_6) δ_C: 171. 5 (*C*O); 66.9 (*C*H-(CH_3)$_2$); 47.3 (*C*H-NH_3^+), 38.2 (CH_2-COO^{i}Pr), 30.6 (*C*H-CH_2COO^{i}Pr), 26.0 (2xCH_2),25.9 (2xCH_2), 21.5 (CH_3).

trans: **^{1}H NMR** (500 MHz, DMSO-d_6) δ_H: 8.10 (3H, br, NH_3^+), 4.88 (1H, quint, *J*=6.25 Hz, C*H*-(CH_3)$_2$), 2.88 (1H, tt, *J*=11.8 Hz, J=3.9 Hz, CH_{ax}-NH_3^+), 2.14 (2H, d, *J*=6.96 Hz, CH_2-COO^{i}Pr), 1.95-1.89 (2H, m 2×CH_{eq}), 1.73-1.70 (2H, m, 2×C*H*), 1.62-1.55 (1H, CH_{ax}-CH_2COO^{i}Pr), 1.33 (2H, qd, *J*=12.7 Hz, *J*=3.2 Hz, 2×CH_{ax}), 1.17 (6H, d, *J*=1.75 Hz, 2×CH_3), 1.02 (2H, qd, *J*=12.8 Hz, *J*=3.2 Hz, 2×C*H*);

^{13}C NMR (125 MHz, DMSO-d_6) δ_C: 171.3 (*C*O); 66.9 (*C*H-(CH_3)$_2$); 48.9 (*C*H-NH_3^+), 40.6 (CH_2-COO^{i}Pr), 33.2 (*C*H-CH_2COO^{i}Pr), 29.8 (2xCH_2), 29.7 (2xCH_2), 21.5 (CH_3).

cis/trans-4-Benzylcyclohexan-1-aminium chloride (*cis/trans*-**1c**·HCl)

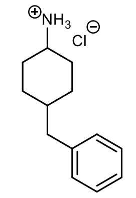

cis/trans-**1c**·HCl

Reaction of 4-benzylcyclohexyl-1-one **2c** (1.50 g, 7.97 mmol) and 10% Pd/C (0.45 g) with ammonium formate (3.01 g, 47.8 mmol) in methanol (60 ml) afforded *cis/trans*-4-(2-ethoxy-2-oxoethyl)cyclohexane-1-amine *cis/trans*-**1c** (0.29 g, 19 %yield) as liquid. Lastly after the introducing of HCl-gas the *cis/trans* diastereomeric mixture of 4-(2-ethoxy-2-oxoethyl)cyclohexan-1-aminium chloride *cis/trans*-**1c**·HCl (0.22 g, 12 % yield) was formed as white solid.

HRMS: (*m/z*): [M^+] calcd. for $C_{13}H_{20}N$, 190.15903; found 190.15850.

IR (liquid film) ν_{max}: 3073, 2610, 2035, 1610, 1511, 1494, 1453, 1392, 1347, 1203, 1062, 744, 701 cm^{-1}.

cis:trans=1.00:1.08 (^{1}H-NMR)

cis: **^{1}H NMR** (500 MHz, DMSO-d_6) δ_H: 8.14 (3H, br, NH_3^+), 7.29-7.25 (2H, m, ArH_{meta}), 7.19-7.13 (3H, m, ArH_{para}, ArH_{orto}), 3.14-3.13 (1H, m, C*H*-NH_3^+), 2.55 (2H, d, *J*=7.64 Hz, CH_2-Ph), 1.78-1.71 (3H, m, 2×C*H*, CH_{ax}-CH_2Ph), 1.66-1.59 (2H, m, 2×C*H*), 1.44-1.41 (4H, m, 4×C*H*);

^{13}C NMR (125 MHz, DMSO-d_6) δ_C: 140.6 (Ar*C*), 128.7 (2xArC_{orto}), 128.1 (2xArC_{meta}), 125.7 (Ar*C, para*), 47.6 (*C*H-NH_3^+), 39.3 (*C*H_2-Ph), 35.4 (*C*H-CH_2-Ph), 26.0 (*C*H_2), 25.9 (*C*H_2).

trans: **^{1}H NMR** (500 MHz, DMSO-d_6) δ_H: 8.14 (3H, br, NH_3^+), 7.29-7.25 (2H, m, ArH_{meta}), 7.19-7.13 (3H, m, ArH_{orto}, ArH_{para}), 2.88 (1H, tt, *J*=11.8 Hz, *J*=3.2 Hz, CH_{ax}-NH_3^+), 2.45 (2H, d, *J*=6.9 Hz, CH_2-Ph), 1.93-1.91 (2H, m, 2×CH_{eq}), 1.66-1.59 (2H, m, 2×CH_{eq}), 1.44-1.41 (1H, m, CH_{ax}-CH_2Ph), 1.28 (2H, qd, *J*=12.61 Hz, *J*=3.2 Hz, 2×CH_{ax}), 1.00 (2H, qd, *J*=13.58 Hz, *J*=2.9 Hz, 2×CH_{ax}) ;

^{13}C NMR (125 MHz, DMSO-d_6) δ_C: 140.3 (Ar*C*), 128.8 (2xArC_{orto}), 128.0 (2xArC_{meta}), 125.7 (ArC_{para}), 49.3 (*C*H-NH_3^+), 42.3 (*C*H_2-Ph), 37.9 (*C*H-CH_2Ph), 30.0 (2x*C*H_2), 29.9 (2x*C*H_2).

cis/trans-4-Phenylcyclohexan-1-aminium chloride (*cis/trans*-**1d**·HCl)

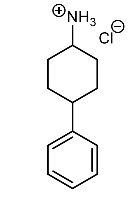

cis/trans-**1d**·HCl

Reaction of 4-phenylcyclohexane-1-one **2d** (4.00 g, 22.9 mmol) and 10% Pd/C (0.40 g) with ammonium formate (8.66 g, 137.4 mmol) in methanol (80 ml) afforded *cis/trans*-4-(2-ethoxy-2-oxoethyl)cyclohexane-1-amine **1d** (2.93 g, 73 %yield) as liquid. Lastly after the introducing of HCl-gas the *cis/trans* diastereomeric mixture of 4-(2-ethoxy-2-oxoethyl)cyclohexan-1-aminium chloride *cis/trans*-**1d·HCl** (2.2 g, 45 % yield) was formed as white solid.

HRMS: (*m/z*): [M^+] calcd. for $C_{12}H_{18}N$, 176.14338; found 176.14302.

IR (liquid film) ν_{max}: 2939, 2544, 2038, 1610, 1504, 1451, 1390, 1182, 1073, 1020, 758, 700 cm^{-1}.

cis:trans= 1.00:3.70 (^{1}H NMR)

cis: **^{1}H NMR** (500 MHz, DMSO-d_6) δ_H: 8.03 (3H, br, NH_3^+), 7.34-7.32 (H, m, 2xArH_{orto}), 7.31-7.27 (H, m, 2xArH_{meta}), 7.19-7.17 (H, m, ArH_{para}), 3.42-3.41 (1H, m, CH_{eq}-NH_3^+), 2.57 (1H, tt, J=11.4 Hz, J=3.4 Hz, CH_{ax}-Ph), 2.20-2.16 (2H, m, 2xCH_{eq}), 1.91-1.85 (2H, m, 2xCH_{eq}), 1.63-1.43 (4H, m, 4xCH_{ax});

^{13}C NMR (126 MHz, DMSO-d_6) δ_C: 146.2 (Ar*C*), 128.2 (2xAr*C*H_{meta}), 126.9 (2xAr*C*H_{orto}), 125.9 (Ar*C*H_{para}), 45.8 (*C*H-NH_3^+), 41.7 (*C*H-Ph), 27.8 (*C*H_2); 26.6 (*C*H_2).

trans: **^{1}H NMR** (500 MHz, DMSO-d_6) δ_H: 8.03 (3H, br, NH_3^+), 7.31-7.27 (H, m, 2xArH_{meta}), 7.24-7.23 (H, m, 2xArH_{orto}), 7.19-7.17 (H, m, ArH_{para}), 3.06 (1H, tt, *J*=11.6 Hz, *J*=3.9 Hz, CH_{ax}-NH_3^+), 2.47 (1H, tt, *J*=12.0 Hz, *J*=3.4 Hz, CH_{ax}-Ph), 2.10-2.00 (2H, m, 2xCH_{eq}), 1.88-1.79 (2H, m, 2xCH_{eq}). 1.58-1.43(4H, m, 4xCH_{ax});

^{13}C NMR (126 MHz, DMSO-d_6) δ_C: 146.0 (Ar*C*), 128.2 (2xAr*C*H_{meta}), 126.6 (2xAr*C*H_{orto}), 126.0 (Ar*C*H_{para}), 48.9 (*C*H-NH_3^+), 42.2 (*C*H-Ph), 31.4 (*C*H_2); 30.4 (*C*H_2).

1.3.1.3 GC data and chromatograms of investigated compounds

Table S1. GC data of reference substrates and products

Entry	Amine	Column	Temp. program	Retention time (min) Ketone	Amide[a] (**7a-d**)		Resp. factor **2:7**	Ref. chrom.
1	**1a**	[b]	[c]	1.95	3.93	4.11	1:1.03	Figure S2
2	**1b**	[b]	[c]	1.58	4.20	4.40	1:0.96	Figure S3
3	**1c**	[b]	[c]	2.74	6.24	6.56	1:0.89	Figure S4
4	**1d**	[b]	[c]	2.32	5.17	5.51	1:1.06	Figure S5

[a] After derivatization of the amine **1a-d** to acetamide **7a-d** with Ac_2O.
[b] HP-5.
[c] 180-210 °C, 5 °C min^{-1}; 210 °C, 1 min.

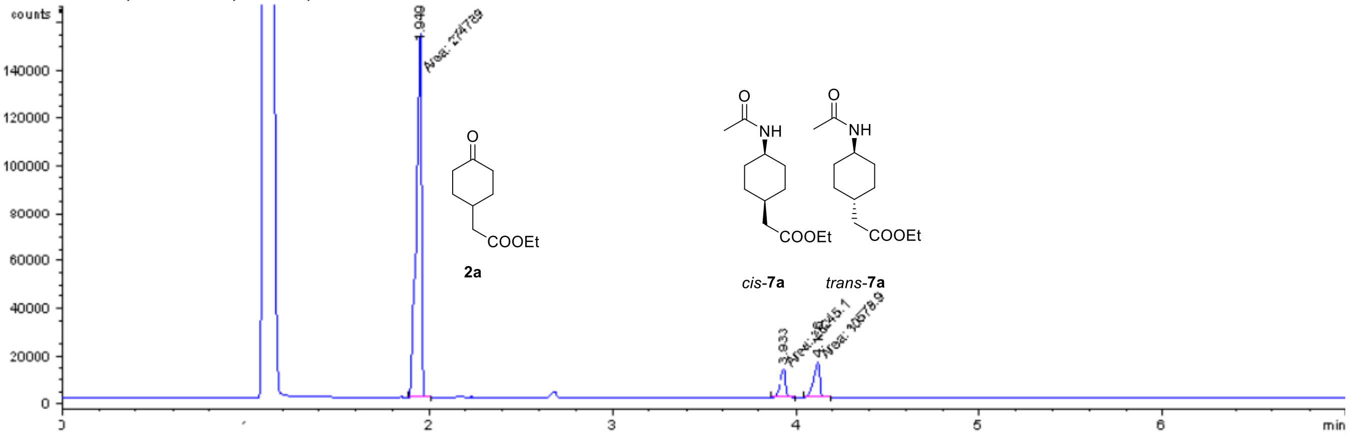

#	Time	Area	Height	Width	Area%	Symmetry
[illegible]	1.949	274783.8	157245.5	0.0291	83.115	2.991
2	3.933	25245.1	11782.1	0.0357	7.636	1.978
3	4.110	30570.9	14540.5	0.005	[illegible].249	2.202

Figure S2. GC chromatogram of **2a**, *cis*-**7a** and *trans*-**7** (HP-5; temperature program: 180-210 °C 5 °C/min, 210 °C 1 min).

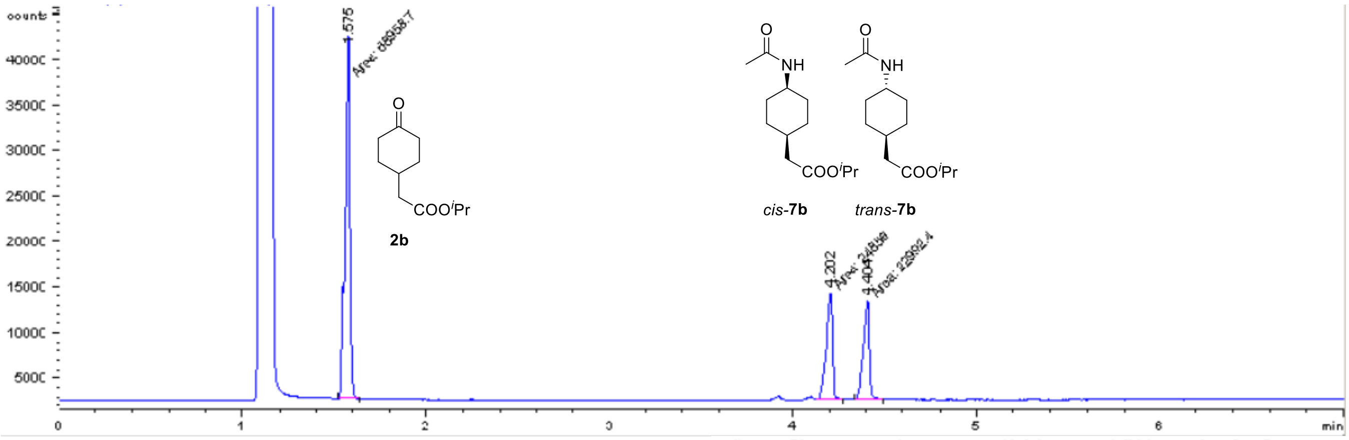

#	Time	Area	Height	Width	Area%	Symmetry
[illegible]	1.575	689587	40297	0.0285	59.036	1.693
2	4.202	2485[illegible]	11673.2	0.0355	21.280	2.142
3	4.404	229924	10772.9	0.0356	19.684	1.845

Figure S3. GC chromatogram of **2b**, *cis*-**7b** and *trans*-**7b** (HP-5; temperature program: 180-210 °C 5 °C/min, 210 °C 1 min).

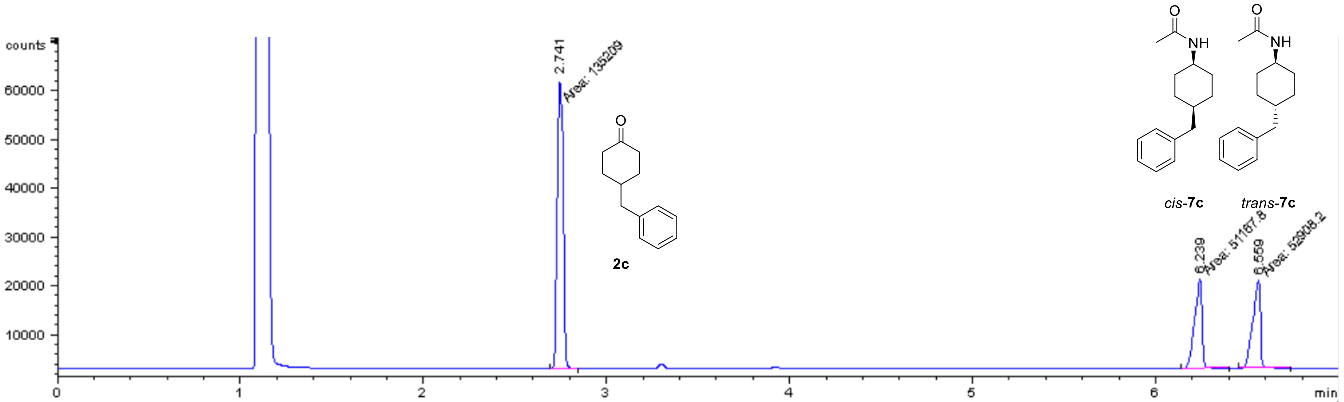

#	Time	Area	Height	Width	Area%	Symmetry
1	2.741	135209.4	59311.8	0.038	56.506	0.693
2	6.239	51167.8	18410.4	0.0463	21.384	2.409
3	6.559	52908.2	18247.4	0.0483	22.111	2.798

Figure S4. GC chromatogram of **2c**, *cis*-**7c** and *trans*-**7c** (HP-5; temperature program: 180-210 °C 5 °C/min, 210 °C 1 min).

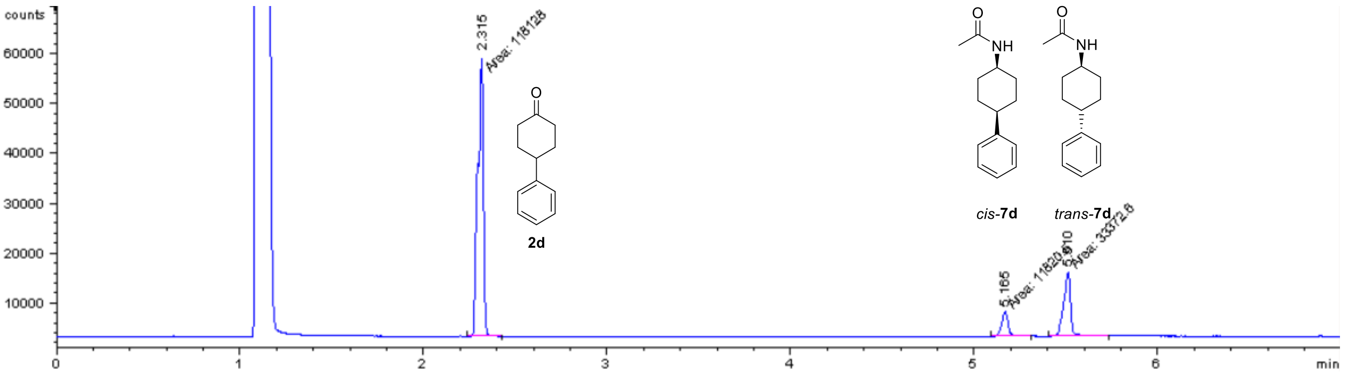

#	Time	Area	Height	Width	Area%	Symmetry
1	2.315	118128.1	56211.8	0.035	72.329	2.328
2	5.165	11820.5	4954.9	0.0398	7.238	1.06
3	5.51	33372.6	12959.9	0.0429	20.434	1.872

Figure S5. GC chromatogram of **2d**, *cis*-**7d** and *trans*-**7d** (HP-5; temperature program: 180-210 °C 5 °C/min, 210 °C 1 min).

1.3.2 *Immobilization of recombinant E. coli whole-cells overexpressing TAs together with hollow silica microspheres as support by entrapment in a sol-gel system*

The full description of the whole-cell TA immobilization was published earlier.[S17] *Briefly*: a silica sol prepared from tetraethyl orthosilicate using sonication technique in acidic phase was kept at 4 °C for overnight. To this silica sol, a homogeneous suspension of wet-cell-paste (harvested from the corresponding TA-overexpressing fermentation) and MAT540 support in phosphate buffer was added. After intensive shaking, gelation started approximately within 30 min at room temperature. The gel was aged at 4 °C for 48 h. The crude whole-cell biocatalyst was washed with distilled water, and dried at room temperature for overnight. The biocatalyst was stored at 4 °C.

1.3.3 *Expression, purification and covalent immobilization of CvS-TAW60C on bisepoxide activated polymer support*

The description of expression and purification covalent immobilization of *CvS*-TA_{W60C} can be found in the original papers.[S9,S10] *CvS*-TA_{W60C} was overexpressed in *E. coli* BL21(DE3) at 10 L scale at Fermentia and purified on Ni-NTA resin according to the protocol published earlier.[S18] Cofactor PLP was added to stock solutions of *CvS*-TA_{W60C} which were kept at -20 °C in 20% glycerol solution until further use. Immobilization of purified *CvS*-TA_{W60C} was performed on glycerol-1,3-diglycidyl ether-activated ethyleneamine-functionalized methacrylic polymer support (ReliZyme™ EA403/S) as published earlier.[S19] The biocatalyst was stored at 4 °C.

A brief summary of the methods: Overexpression of *CvS*-TA_{W60C} was achieved in *E. coli* BL21(D3) containing the recombinant pET28a(+) plasmid with the gene of *CvS*-TA_{W60C}. Production of the recombinant *CvS*-TA_{W60C} with a His-tag was performed in 10 L of Terrific broth supplemented with kanamycin (50 µg mL^{-1}) as antibiotic marker in agitated submerse fermentation. The culture was inoculated aseptically with 1% of seed culture and incubated at 37 °C, 300 rpm agitation, 5 L min^{-1} aeration and 0.35 bar overpressure; the pH was set to 7.0 (±0.1) using 2M NaOH. At an optical density between 0.8 and 1.0 (measured at 600 nm), isopropyl β–D-1-thiogalactopyranoside (IPTG) was added to a final concentration of 1 mM to induce the expression of *CvS*-TA_{W60C}. The fermentation was continued at 28 °C for about 16 hours. The cells were then harvested by centrifugation.

After fermentation, the *E. coli* cells containing *CvS*-TA_{W60C} were disrupted by French press, centrifuged and crude cell extract was purified by Ni-NTA resin as described previously.[S20] The molecular weight of *CvS*-TA_{W60C} is ~104 kDa as homodimer. The purified enzyme was investigated by SDS-PAGE (Supplementary Fig. S6, referring to the purity of the monomeric units of *CvS*$_{W60C}$-TA at ~50 kDa). The stock solution of *CvS*$_{W60C}$-TA was kept at -20 °C in 20% glycerol solution until further use.

Surface activation of ethyleneamine-functionalized methacrylic polymer support (RelyZyme™ S EA403-resin) was performed by glycerol-1,3-diglycidyl ether in isopropanol.[S19] In a Erlenmeyer flask purified *CvS*-TA_{W60C} was diluted with HEPES buffer, and then the immobilization support (enzyme:support ratio= 1:10) was added to the solution. The resulted suspension was shaken at 900 rpm for 24 h at 25 °C. The *CvS*-TA_{W60C} was centrifugated, washed with HEPES buffer. *CvS*-TA_{W60C} concentrations in the supernatant before and after the immobilization were determined spectrophotometrically. The imm-*CvS*$_{W60C}$-TA biocatalyst was dried at room temperature for overnight and stored at 4 °C.

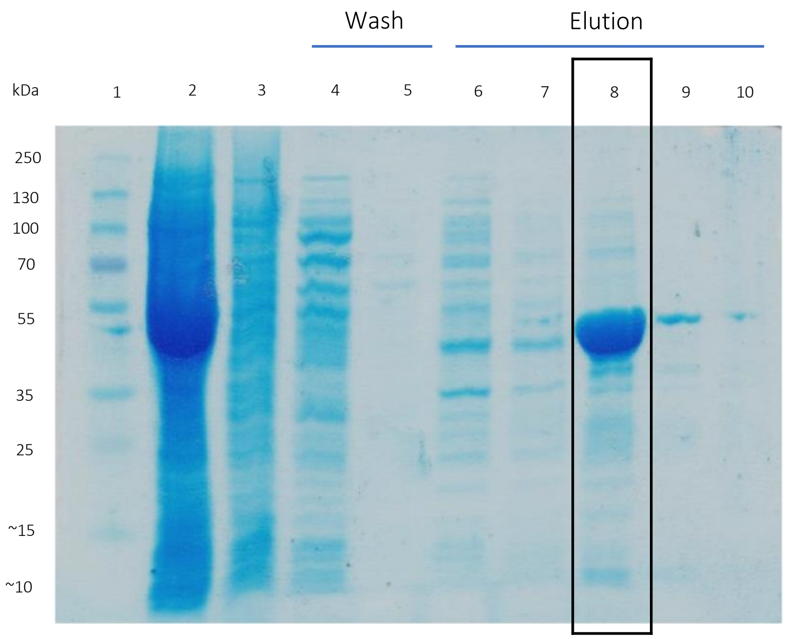

Figure S6 The SDS-PAGE analysis of the purification procedure of *CvS*-TA$_{W60C}$ on Ni-NTA resin. Solutions for washing and elution: LS (50 mM HEPES, 30 mM KCl); LIm (25 mM imidazole in LS solution); HIm (500 mM imidazole in LS solution). Lanes: 1 - protein marker; 2 - *CvS*-TA$_{W60C}$ lysate load; 3 - flow-through; 4 - LS1 wash; 5 - LS2 wash; 6 - LIm1 elution; 7 - LIm2 elution; 8 - HIm1 elution; 9 - HIm2 elution; 10 - HIm3 elution.

*1.3.4 Exploring the reaction parameters (temperature, cosolvents) for biotransformations with imm-CvS-TAW60C using the kinetic resolution of rac-**8** as test reaction*

1.3.4.1 Test reaction for exploring the reaction conditions

For exploring the reaction parameters (heat and cosolvent tolerance of imm-*CvS*-TA$_{W60C}$) kinetic resolution of *rac*-**8** (Supplementary Fig. S7) was applied as test reaction with slight modifications compared to our previous studies. [S17]

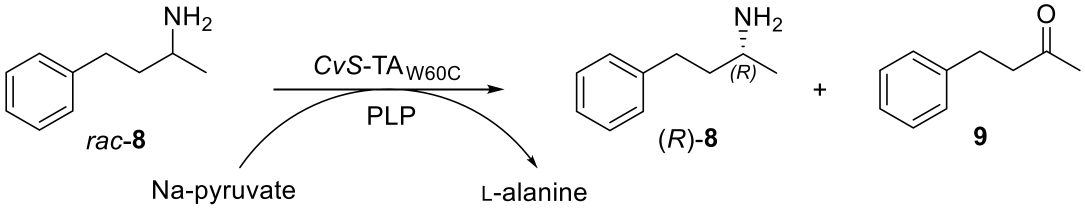

Figure S7 Kinetic resolution of *rac*-**8** catalyzed by imm-*CvS*-TA$_{W60C}$ as test reaction

The imm-*CvS*-TA$_{W60C}$ (50 mg) was suspended in TRIS buffer (1.48 ml, 50 mM, pH=7.0) containing pyridoxal-5'-phosphate monohydrate (0.2 mM) in 4 mL vials and incubated for 1 h. Then the *rac*-**8** (20 mM) was added to the suspension and incubated for 30 min again. Finally, sodium pyruvate (0.6 eq.) was added. The final reaction volume was 2 mL. The reaction mixture was shaken on an orbital shaker (600 rpm) at 30 °C. After addition of sodium hydroxide (100 µL, 1 M) to the samples taken from the reaction mixture (170 µL), the resulting mixture was extracted with ethyl acetate (800 µL). After derivatizing the amines (*R*)-**8** in the extract by addition of acetic anhydride (10 µL, 60 °C, 1 h), the organic phase was dried over Na_2SO_4 and analyzed by gas chromatography on Agilent 4890 equipment equipped with Hydrodex β-6 TBDM, H_2 carrier gas, injector: 250 °C, detector: 250 °C, head pressure: 12 psi, split ratio: 50:1]. A typical GC chromatogram of the analysis is shown as Supplementary Fig. S8.

1.3.4.2 GC analysis of the kinetic resolution from *rac*-**8**

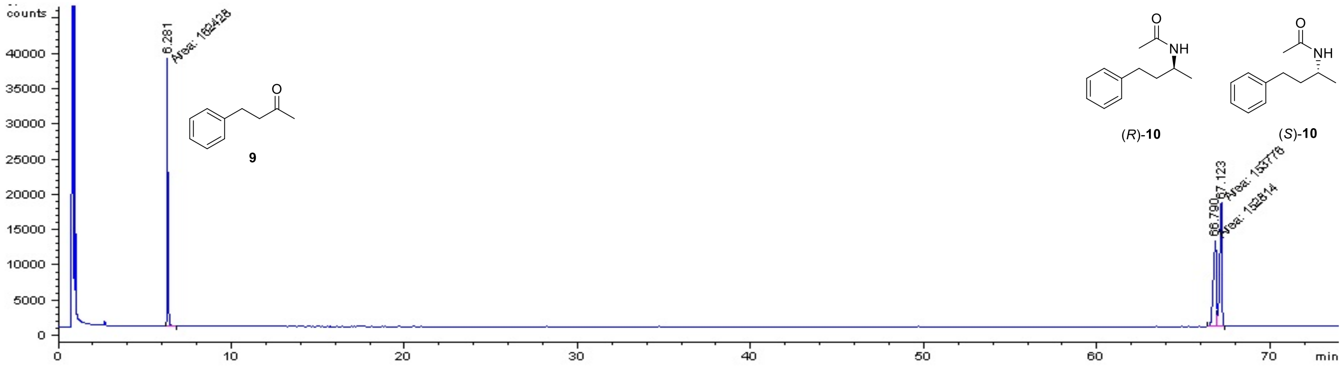

#	Time	Area	Height	Width	Area%	Symmetry
1	6.281	162428.1	38072.3	0.0711	34.631	0.324
2	66.79	152814.2	12151.9	0.2096	32.582	2.124
3	67.123	153776.1	17563.8	0.1459	32.787	1.71

Figure S8. Typical GC chromatogram of a sample from kinetic resolution of *rac*-**8**.
Signals are shown for **9**, (*R*)-**10** and (*S*)-**10** (acetamides (*R*)-**10** and (*S*)-**10** were obtained from the corresponding amine (*R*)-**8** and (*S*)-**8** by derivatization to *rac*-**10** with A_2O; column: Hydrodex β-6 TBDM; temperature program: 130 °C, 60 min; 130-140 °C, 2 °C/min, 140-180 °C, 10 °C/min, 180 °C, 3 min; response factor **9**:**10**=1:1.20).

1.3.4.3 Screening conditions for biotransformations with imm-CvS-TA_{W60C} in batch mode

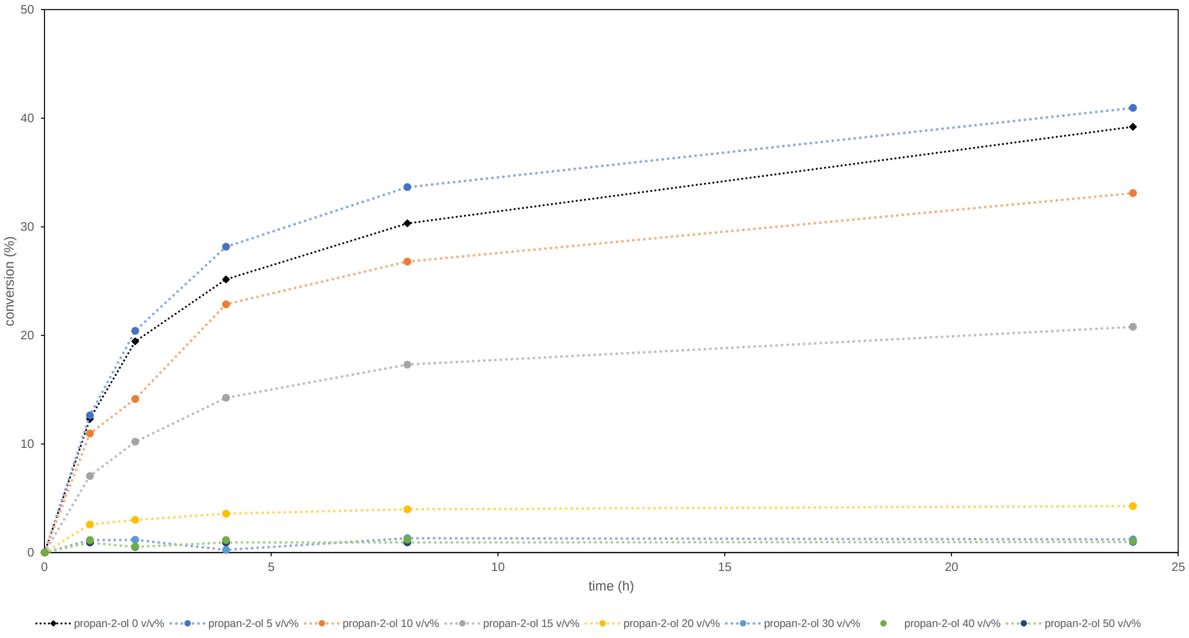

Figure S9 Effect of propan-2-ol on kinetic resolution of *rac*-**8** in batch mode. Time course profiles are shown for reactions in the presence of different v/v% of propan-2-ol concentration [imm-*CvS*-TA_{W60C} (50 mg), *rac*-**8** (20 mM), sodium pyruvate (0.6 eq.), PLP (0.2 mM), TRIS buffer containing propan-2-ol (50 mM, pH=7.0) in a final volume of 2 mL, at 30°C and 600 rpm)].

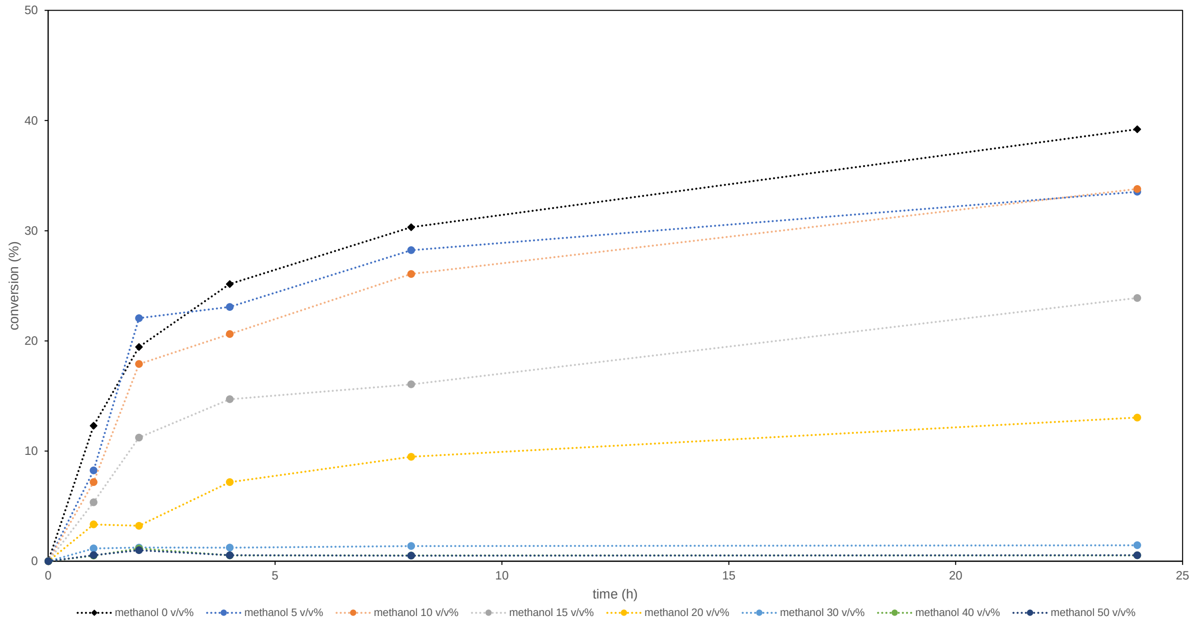

Figure S10 Effect of metanol on kinetic resolution of *rac*-**8** in batch mode. Time course profiles are shown for reactions in the presence of different v/v% of methanol concentration [imm-*CvS*-TA$_{W60C}$ (50 mg), *rac*-**8** (20 mM), sodium pyruvate (0.6 eq.), PLP (0.2 mM), TRIS buffer containing methanol (50 mM, pH=7.0) in a final volume of 2 mL, at 30°C and 600 rpm)].

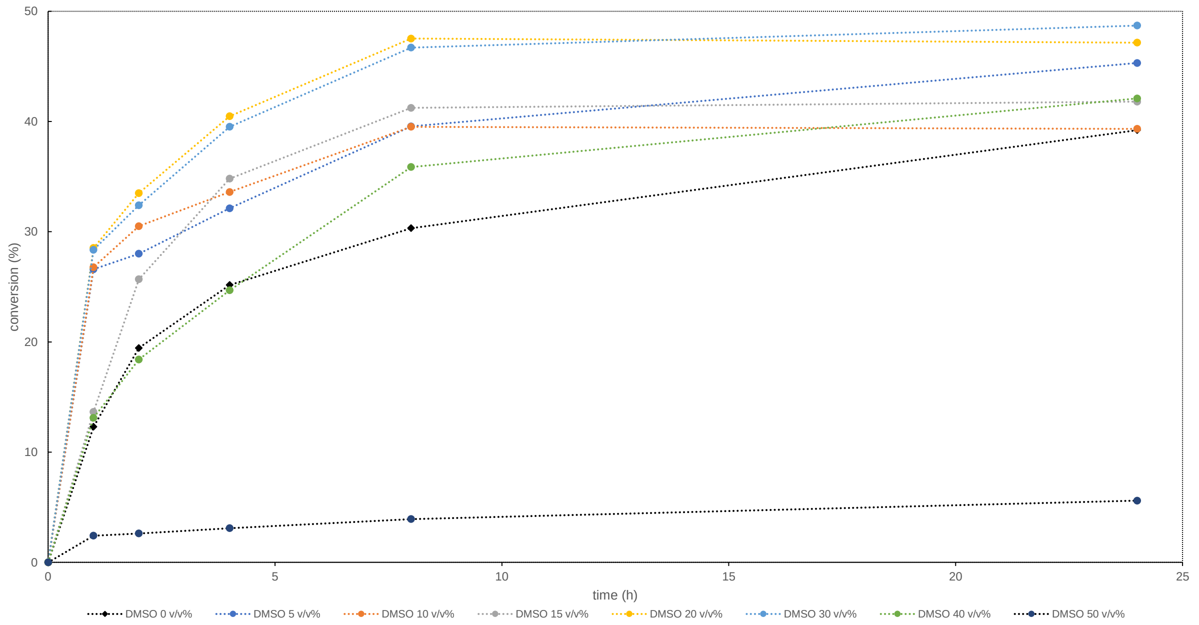

Figure S11 Effect of dimethyl sulfoxide on kinetic resolution of *rac*-**8** in batch mode. Time course profiles are shown for reactions in the presence of different v/v% of dimethyl sulfoxide [imm-*CvS*-TA$_{W60C}$ (50 mg), *rac*-**8** (20 mM), sodium pyruvate (0.6 eq.), PLP (0.2 mM), TRIS buffer containing dimethyl sulfoxide (50 mM, pH=7.0) in a final volume of 2 mL, at 30°C and 600 rpm)].

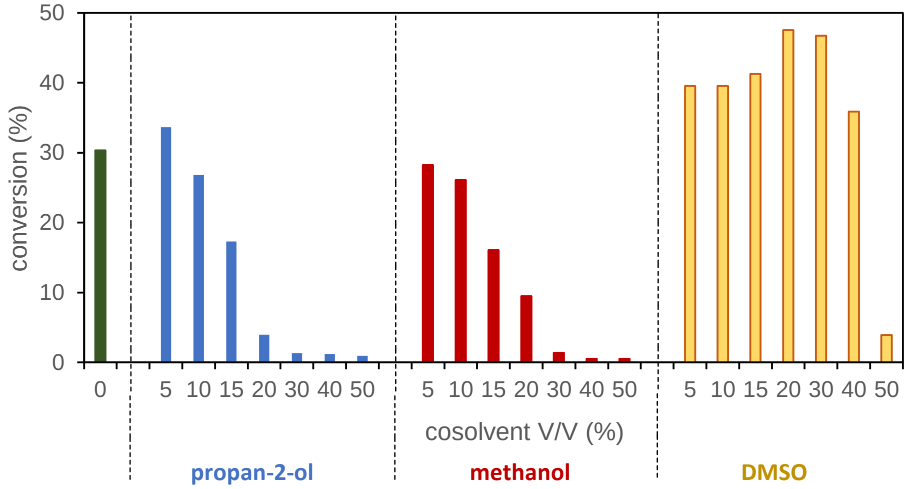

Figure S12 Comparison of the effect of cosolvents on the kinetic resolution of *rac*-**8** in batch mode at 8 h reaction time. Reaction conditions: imm-*CvS*-TA$_{W60C}$ (50 mg), *rac*-**8** (20 mM), sodium pyruvate (0.6 eq.), PLP (0.2 mM), TRIS buffer (50 mM, pH=7.0) at 30 °C, at 600 rpm.

In the test reactions, the effect of different kind and amount of cosolvents (propan-2-ol, methanol, and dimethyl sulfoxide) on the KR of *rac*-**8** with imm-*CvS*-TA$_{W60C}$ was examined modelling solubility enhancement in aqueous media for relatively hydrophobic substrates. Compared to cosolvent free reaction as benchmark, only the 5% propan-2-ol system increased the activity slightly, while methanol had no positive effect for transamination at all (Supplementary Figure S12). The imm-*CvS*-TA$_{W60C}$ exhibited good resistance to 5, 10, 15, 20, 30 and 40 V/V% dimethyl sulfoxide with improved activity, while 50V/V% DMSO deactivated the enzyme (Supplementary Figure S12). This advantageous enhancement can be rationalized by assuming significant stabilization of the tertiary structure of *CvS*-TA$_{W60C}$ in this immobilized form by DMSO.[21] Since the conversion could be improved in medium containing propan-2-ol or DMSO, these two solvents as auxiliary cosolvents were investigated further in continuous-flow experiments.

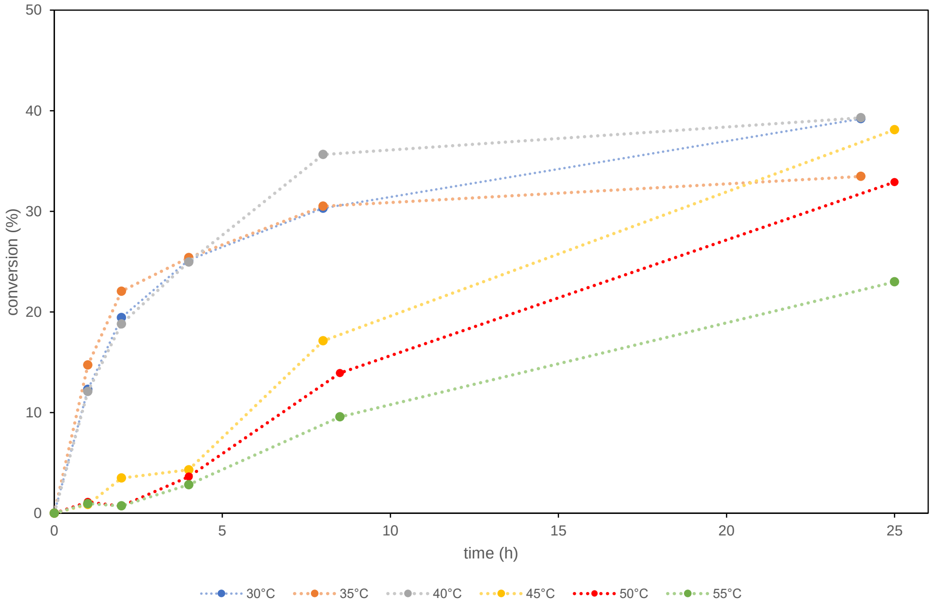

Figure S13 Effect of temperature on kinetic resolution of *rac*-**8** in batch mode. Time course profiles are shown for reactions at different temperatures between 30–55 °C [imm-*CvS*-TA$_{W60C}$ (50 mg), *rac*-**8** (20 mM), sodium pyruvate (0.6 eq.), PLP (0.2 mM), TRIS buffer (50 mM, pH=7.0) in a final volume of 2 mL, at 600 rpm)].

In the test reactions, the effect of temperature (30–55 °C) on the KR of *rac*-**8** with imm-*CvS*-TA_{W60C} was examined (Supplementary Fig. S13). Since the highest rate could be achieved at 40 °C, the further continuous-flow experiments were conducted around this temperature (±10 °C).

1.3.4.4 Screening conditions of biotransformations with imm-CvS-TAW60C in continuous-flow mode

1.3.4.4.1 Equipment for continuous-flow processes

The laboratory scale flow reactor (Supplementary Fig. S14) comprised of syringe pump (Asia® Syringe Pump system, Syrris Ltd., Royston, UK) or Knauer Azura P4.1S or 2.1S isocratic HPLC pump) attached to CatCart™ columns (Thalesnano, Budapest, Hungary; stainless steel, inner diameter: 4 mm; total length: 70 mm; packed length: 65 mm; inner volume: 0.816 mL) filled with imm-*CvS*-TA (filling weights: 375±12 mg/column) in an in-house made stainless steel metal block reactor heater. The column was sealed by filter membranes made of PTFE [Whatman® Sigma-Aldrich, WHA10411311, pore size 0.45 µm]. The sealing elements were made of PTFE. PTFE tubing (1/16” outer diameter and 0.8 mm inner diameter, VICI AG International, Schenkon, Switzerland) and PEEK fingertight (Sigma Aldrich) were used to connect columns (purchased from commercial vendors).

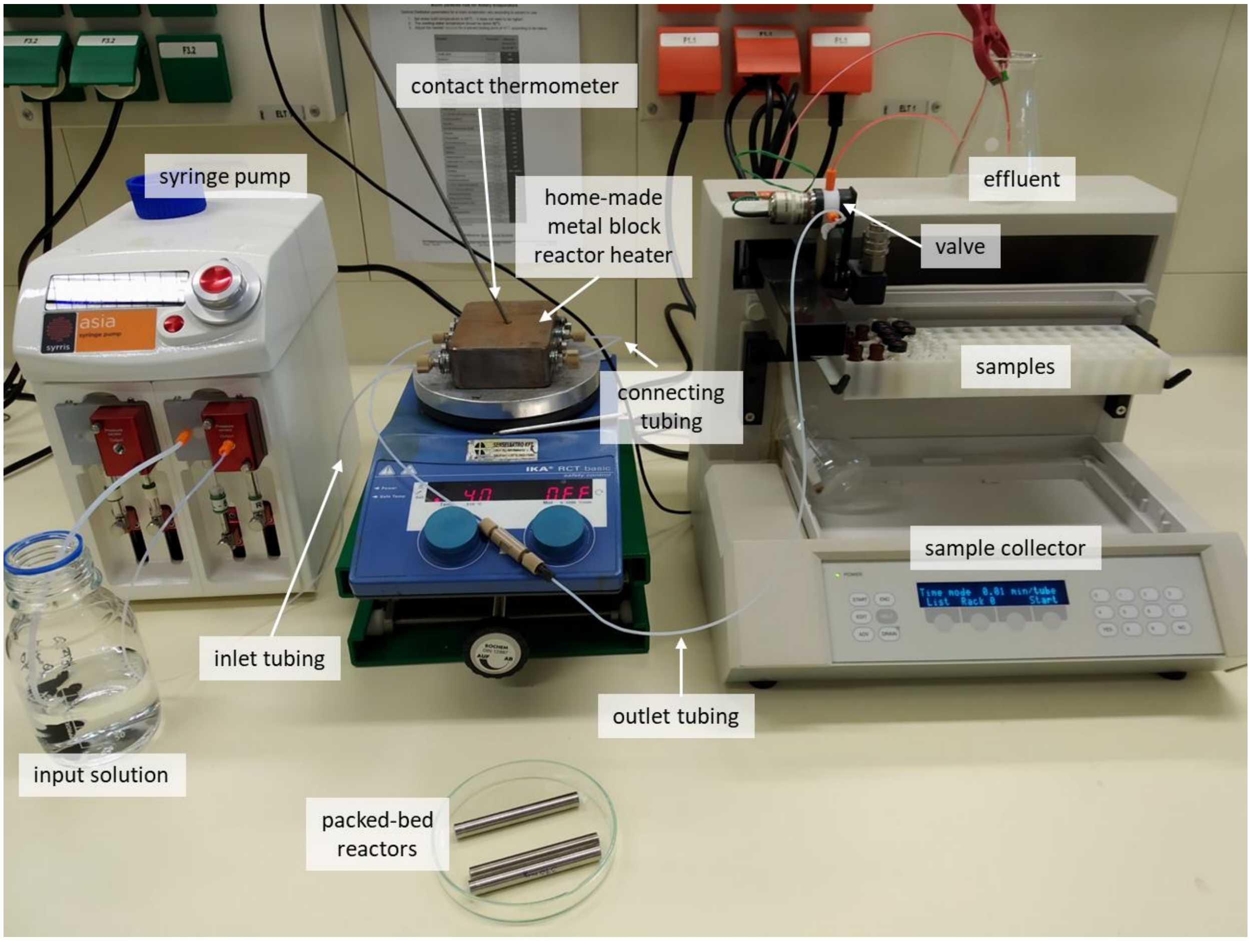

Figure S14 The benchtop system used for the continuous-flow experiments.

1.3.4.4.2 Residence time

To estimate the residence time in a packed-bed column, the volume of the reaction solvent is required.

The difference between the mass of a dry imm-*CvS*-TA_{W60C}-filled and a buffer loaded imm-*CvS*-TA_{W60C}-filled column is the mass of reaction solvent (m_{solv}) within the filled column.

$$m_{solv} = m_{gross} - m_{dry} = 21.8647 - 21.2665 = 0.5982\ mg \qquad \text{(Equation S1)}$$

In Equation S1, m_{solv} is the net mass of the solvent (50 mM HEPES buffer, pH=7.0) in the imm-*CvS*-TA_{W60C}-filled column, m_{gross} is the mass of buffer loaded imm-*CvS*-TA_{W60C}-filled column (g), and m_{dry} is the mass of dry imm-*CvS*-TA_{W60C}-filled column (g).

The density of the solvent (ρ_{solv}) (50 mM HEPES, pH=7.0) was determined by measuring the mass of samples (m_{cal}= 27.6851±0.2156 g, triplicate) filled in calibrated flasks of known volume (V_{cal}= 25 mL) according to Equation S2.

$$\rho_{solv} = \frac{m_{cal}}{V_{cal}} = \frac{27.6851 \pm 0.2156}{25} = 1.107 \pm 0.0086\ g\ mL^{-1} \qquad \text{(Equation S2)}$$

The solvent-filled volume (V_{solv}) of a single column can be determined by the Equation S3:

$$V_{solv} = \frac{m_{solv}}{\rho_{solv}} = \frac{0.5982}{1.107} = 0.540\ mL \qquad \text{(Equation S3)}$$

The apparent residence time (τ_{app}) in a single column at the actual flow-rate (f= 0.01 mL·min^{-1}) can be determined by the Equation S4:

$$\tau_{app} = \frac{V_{solv}}{f} = \frac{0.540}{0.01} = 54\ min \qquad \text{(Equation S4)}$$

This residence time (τ_{app}) should be considered as apparent for such macroporous biocatalyst systems since the solvent-filled volume (V_{solv}) is the sum of volume of interparticle solvent content (in which the compound molecules are moving at the same linear rate as the solvent molecules) and the volume of solvent content in the pores of the particles (within the pores the substrate and product molecules are moving only by diffusion, thereby this part is flow-rate independent). Therefore, the residence time calculated by using the solvent-filled volume (V_{solv}) containing unknown fraction of flow-independent in-pore solvent volume for the macroporous beads should be lower than a non-porous particles-filled system with the same solvent-filled volume (V_{solv}).

1.3.4.4.3 Optimization of reaction parameters for kinetic resolution of *rac*-**8** in continuous-flow mode

Based on preliminary continuous-flow experiments using different buffers (TRIS, HEPES, phosphate, data not shown), HEPES was selected as reaction media for the further experiments.

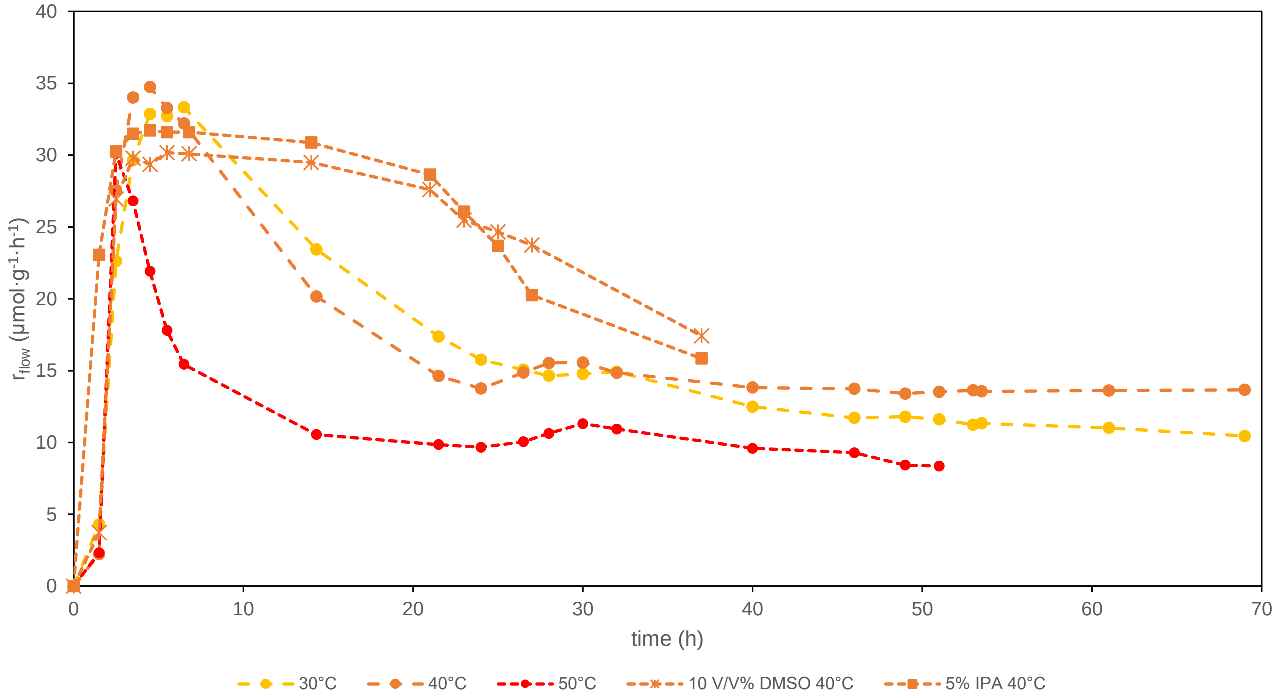

Figure S15. Effect of operational temperature and cosolvents on continuous-flow kinetic resolution of *rac*-**8**. Reaction conditions: imm-*CvS*-TA_{W60C} (375±12 mg mg), *rac*-**8** (20 mM), sodium pyruvate (0.6 eq.), PLP (0.2 mM), HEPES buffer (50 mM, pH=7.0) at 20 µL min^{-1}.

The kinetic resolution of *rac*-**8** as model reaction for transamination with imm-*CvS*-TA_{W60C} was examined in continuous-flow mode (**Figure S15**). Based on the batch mode results, the continuous-flow KR of *rac*-**8** was performed in packed-bed reactor at different temperatures (30 °C, 40 °C, and 50 °C). Without cosolvent at 50 °C deactivation of the imm-*CvS*-TA_{W60C} was quite rapid, and in all cases, after an initial activity loss a longer stable period was observed. On that basis, two forms—one more active but less stable and another less active but more stable—of the imm-*CvS*-TA_{W60C} could be assumed. At 30 °C and 40 °C the residual less active imm-*CvS*-TA_{W60C} form seemed to be more stable. Since the highest degree of stable conversion could be achieved at 40 °C, this temperature was investigated further employing the best amounts of cosolvents (5V/V% IPA and 10V/V% DMSO). In both cases the first activity loss was slower, therefore employing DMSO as cosolvent had positive effect on conversion even after 10 hours operation. Based on these results, the deaminations of the targeted diastereomeric *cis/trans*-**1a-d** mixtures by imm-*CvS*-TA_{W60C} in continuous-flow mode was performed at 40 °C using DMSO as cosolvent.

1.3.5 Deamination of cis/trans-1a-d with imm-CvS-TA$_{W60C}$ in continuous-flow mode

1.3.5.1 Investigation of the deamination of *cis/trans*-**1a-d** in continuous-flow mode with systems comprising different number of serially coupled imm-CvS-TA$_{W60C}$ columns

Table S2. Results of deamination of *cis/trans*-**1a-d** catalyzed by imm-*CvS*-TA$_{W60C}$ with different number of columns in continuous-flow mode[a]

Amine	Number of columns							
	n=1		n=2		n=3		n=4	
	$x_{1a\text{-}d}$ (%)[b]	$de_{trans\text{-}1a\text{-}d}$ (%)[b]	$x_{1a\text{-}d}$ (%)[b]	$de_{trans\text{-}1a\text{-}d}$ (%)[b]	$x_{1a\text{-}d}$ (%)[b]	$de_{trans\text{-}1a\text{-}d}$ (%)[b]	$x_{1a\text{-}d}$ (%)[b]	$de_{trans\text{-}1a\text{-}d}$ (%)[b]
**1a**	52.5	86.7	55.9	97.5	61.2	>99.9	-	-
**1b**	23.7	49.6	30.7	72.6	39.5	75.2	50.0	>99.5
**1c**	40.2	>99.5	-	-	-	-	-	
**1d**	13.6	90.9	16.8	>99.5	-	-	-	

[a] Reaction conditions: *cis/trans*-**1a-d** as HCl salt (*cis/trans*-**1a-b** 20 mM, *cis/trans*-**1c-d** 15 mM), sodium pyruvate (1 equiv. to *cis/trans*-**1a-d**) and PLP cofactor (1 n/n%) in HEPES (50 mM, pH=7.0) with DMSO (*cis/trans*-**1a-b** 10% v/v, *cis/trans*-1**c-d** 20% v/v) in packed-bed reactors (n) at 40 °C with 10 µL min^{-1}.
[b] Determined after derivatization with Ac_2O by GC.

1.3.5.2 Production of pure *trans*-**1a-d** by deamination of *cis/trans*-**1a-d** with immobilized CvS-TA$_{W60C}$ in continuous-flow mode

The products from effluents collected during the indicated periods were isolated as HCl salts (for details, see the "Deamination of *cis/trans*-**1a-d** with immobilized *CvS*-TA$_{W60C}$ in continuous-flow mode" part of the Methods section in the main article).

trans-4-(2-Ethoxy-2-oxoethyl)cyclohexan-1-aminium chloride (*trans*-**1a**·HCl)

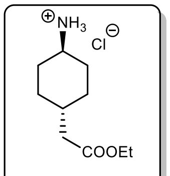

trans-**1a**·HCl

The reaction afforded the product of *trans*-**1a**·HCl from stationary state between 6-54 hour (39.2 mg, isolated yield 30.7%) as a white solid.[S22]
^{1}H NMR (500 MHz, DMSO-*d$_6$*) δ_H: 8.09 (3H, br, N*H*$_3^+$), 4.04 (2H, q, *J*=7.22 Hz, OC*H*$_2$), 2.94-2.83 (1H, m, C*H*$_{ax}$-NH$_3^+$), 2.17 (2H, d, *J*=7.0 Hz, C*H*$_2$-COOEt), 1.93 (2H, br d, *J*=13.5 Hz, 2× C*H*$_{eq}$CHNH$_3^+$), 1.72 (2H, br d, *J*=13.0 Hz, 2×C*H*$_{eq}$), 1.64-1.56 (1H, m, C*H*$_{ax}$-CH$_2$COOEt), 1.32 (2H, qd, *J*=12.4 Hz, *J*=2.9 Hz, 2×C*H*$_{ax}$-CHNH$_3^+$), 1.17 (3H, t, *J*=7.2 Hz, C*H*$_3$), 1.02 (2H, qd, *J*=12.8 Hz, *J*=2.7 Hz, 2×C*H*$_{ax}$);
^{13}C NMR (125 MHz, DMSO-*d$_6$*) δ_C: 171.8 (*C*O), 59.6 (O*C*H$_2$), 48.9 (*C*H-NH$_3^+$), 40.4 (*C*H$_2$-COOEt), 33.1 (*C*H$_2$), 29.8 (*C*H$_2$), 14.0 (*C*H$_3$).
HRMS: (*m/z*): [M$^+$] calcd. for $C_{10}H_{20}O_2N$, 186.14886; found 186.14853.

trans-4-(2-Isopropoxy-2-oxoethyl)cyclohexan-1-aminium chloride (*trans*-**1b**·HCl)

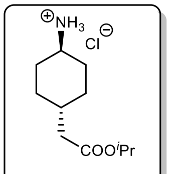

trans-**1b**·HCl

The reaction afforded the product of *trans*-**1b**·HCl from stationary state between 6-37 hour (40.8 mg, isolated yield 46.5%) as a white solid.
^{1}H NMR (500 MHz, DMSO-*d$_6$*) δ_H: 8.05 (3H, br, N*H*$_3^+$), 4.88 (1H, quint, *J*=6.3 Hz, C*H*-(CH$_3$)$_2$), 2.89-2.87 (1H, m, C*H*$_{ax}$-N*H*$_3^+$), 2.14 (2H, d, *J*=6.96 Hz, C*H*$_2$-COO^{i}Pr), 1.94-1.91 (2H, m, 2×C*H*$_{eq}$), 1.72-1.70 (2H, m, 2×CH$_{eq}$), 1.63-1.55 (1H, m, C*H*$_{ax}$-CH$_2$COO^{i}Pr), 1.32 (2H, qd, *J*=12.7 Hz, *J*=3.0 Hz, 2×CH$_{ax}$), 1.17 (6H, d, *J*=6.25 Hz, 2×CH$_3$), 1.02 (2H, qd, *J*=12.9 Hz, *J*=3.1 Hz, 2×CH$_{ax}$;

^{13}C NMR (125 MHz, DMSO-*d$_6$*) δ_C: 171.3 (*C*O), 66.9 (*C*H-(CH_3)$_2$), 48.9 (*C*H-NH_3^+), 40.6 (CH-*C*H_2-COO^{i}Pr), 33.2 (*C*H-CH_2COO^{i}Pr), 29.8 (2x*C*H_2), 29.7 (2x*C*H_2), 21.5 (2x*C*H_3).
HRMS: (*m/z*): [M$^+$] calcd. for $C_{11}H_{22}O_2N$, 200.16451; found 200.16423.

trans-4-Benzylcyclohexan-1-aminium chloride (*trans*-**1c**·HCl)

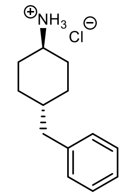

trans-**1c**·HCl

The reaction afforded the product of *trans*-**1c**·HCl from stationary state between 8-43 hour (36.1 mg, isolated yield 54.1%) as a white solid.
^{1}H NMR (500 MHz, DMSO-*d$_6$*) δ_H: 7.98 (3H, br, N*H$_3^+$*), 7.29-7.26 (2H, m, Ar*H$_{meta}$*), 7.19-7.13 (3H, m, Ar*H$_{orto}$* Ar*H$_{para}$*), 2.91-2.86 (1H, m, C*H*$_{ax}$-NH_3^+,), 2.45 (2H, d, J=7.04 Hz, C*H$_2$*-Ph), 1.92-1.89 (2H, m, 2×C*H$_{eq}$*), 1.67-1.65 (2H, m, 2×C*H$_{eq}$*), 1.48-1.39 (1H, m, C*H$_{ax}$*-CH_2-Ph), 1.26 (2H, qd, *J*=13.7 Hz, *J*=3.22 Hz, 2×C*H*$_{ax}$), 1.01 (2H, qd, *J*=13.4 Hz, *J*=3.10 Hz, 2×C*H*$_{ax}$);
^{13}C NMR (125 MHz, DMSO-*d$_6$*) δ_C: 140.3 (Ar*C*), 128.8 (2xAr*CH$_{orto}$*), 128.0 (2xAr*CH$_{meta}$*), 125.7 (Ar*C$_{para}$*), 49.3 (*C*H$_{ax}$-NH_3^+), 42.3 (*C*H_2-Ph), 37.8 (*C*H-CH_2-Ph), 30.0 (2x*C*H_2-CHCH$_2$Ph), 29.9 (2x*C*H_2-CH $_{ax}$NH$_3^+$).
HRMS: (*m/z*): [M$^+$] calcd. for $C_{13}H_{20}N$, 190.15903; found 190.15882.

trans-4-Phenylcyclohexan-1-aminium chloride (*trans*-**1d**·HCl)

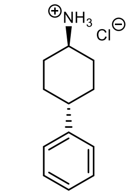

trans-**1d**·HCl

The reaction afforded the product of *trans*-**1d**·HCl from stationary state between 4.5-53 hour (76.6 mg, isolated yield 77.7%) as a whitish-yellow solid.
^{1}H NMR (500 MHz, DMSO-*d$_6$*) δ_H: 8.17 (3H, br, N*H$_3^+$*), 7.29-7.26 (2H, m, Ar*H$_{meta}$*), 7.24-7.22 (2H, m, Ar*H$_{orto}$*), 7.18 (1H, tt, *J*=7.11 Hz, *J*=1.43 Hz, Ar*H$_{para}$*), 3.05-3.03 (1H, m, C*H*$_{ax}$-NH_3^+), 2.46-2.40 (1H, m, C*H*$_{ax}$-Ph), 2.06-2.04 (2H, m, 2×C*H$_{eq}$*), 1.83-1.82 (2H, m, 2×C*H$_{eq}$*); 1.57-1.44 (4H, m, 4×C*H$_{ax}$*);
^{13}C NMR (126 MHz, DMSO-*d$_6$*) δ_C: 146.0 (Ar*C*), 128.2 (Ar*C*H$_{meta}$), 126.6 (Ar*C*H$_{orto}$), 126.0 (Ar*C*H$_{para}$), 48.8 (*C*H-NH_3^+), 42.2 (*C*H-Ph), 31.4 (2x*C*H_2), 30.4 (2x*C*H_2).
HRMS: (*m/z*): [M$^+$] calcd. for $C_{12}H_{18}N$, 176.14338; found 176.14312.

Supplementary references

S1 Pannuri, S.; Kamat, S. V.; Garcia, A. R. M. Thermostable omega-transaminases. *WIPO Pat. Appl.*, WO2006/063336 A2 (2006).

S2 Koszelewski, D.; Göritzer, M.; Clay, D.; Seisser, B.; Kroutil,W. Synthesis of optically active amines employing recombinant ω-transaminases in *E. coli* cells. *ChemCatChem*, **2**, 73–77 (2010).

S3 Iwasaki, A.; Matsumoto, K.; Hasegawa, J.; Yasohara, Y. A novel transaminase, (*R*)-amine:pyruvate aminotransferase, from *Arthrobacter* sp. KNK168 (FERM BP-5228): purification, characterization, and gene cloning. *Appl. Microbiol. Biotechnol.*, **93**, 1563–1573 (2012).

S4 Guan, L. J.; Ohtsuka, J.; Okai, M.; Miyakawa, T.; Mase1 T.; Zhi, Y.; Hou, F.; Ito, N.; Iwasaki, A.; Yasohara, Y.; Tanokura, M. A new target region for changing the substrate specificity of amine transaminases. *Sci. Rep.*, **5**, 10753 (2015).

S5 Savile, C. K.; Janey, J. M.; Mundorff, E. C.; Moore, J. C.; Tam, S.; Jarvis, W. R.; Colbeck, J. C.; Krebber, A.; Fleitz, F. J.; Brands, J.; Devine, P. N.; Huisman, G. W.; Hughes, G. J. Biocatalytic asymmetric synthesis of chiral amines from ketones applied to sitagliptin manufacture. *Science*, **329**, 305–309 (2010).

S6 Cuetos, A.; García-Ramos, M.; Fischereder, E. M.; Díaz-Rodríguez, A.; Grogan, G.; Gotor, V.; Kroutil, W.; Lavandera, I. Catalytic Promiscuity of Transaminases: Preparation of Enantioenriched β-Fluoroamines by Formal Tandem Hydrodefluorination/Deamination. *Angew. Chem. Int. Ed. Engl.*, **55**(9), 3144-3147 (2016).

S7 Mutti, F.G.; Fuchs, C.S.; Pressnitz, D.; Sattler, J.H.; Kroutil, W. Stereoselectivity of four (*R*)-selective transaminases for the asymmetric amination of ketones. *Adv. Synth. Catal.*, **353**, 3227–3233 (2011).

S8 Łyskowski, A.; Gruber, C.; Steinkellner, G.; Schürmann, M.; Schwab, H.; Gruber, K.; Steiner, K. Crystal Structure of an (*R*)-Selective ω-Transaminase from *Aspergillus terreus*. *PLoSONE*, **9**(1), e87350 (2014).

S9 Cassimjee, K.E.; Humble, M.S.; Land, H.; Abedi, V.; Berglund, P. *Chromobacterium violaceum* ω-transaminase variant Trp60Cys shows increased specificity for (S)-1-phenylethylamine and 4'-substituted acetophenones and follows Swain–Lupton parameterisation. *Org. Biomol. Chem.*, **10**, 5466–5470 (2012).

S10 Humble, M. S.; Cassimjee, K. E.; Abedi, V.; Federsel, H.-J.; Berglund, P. Key aminoacid residues for reversed or improved enantiospecificity of an ω-transaminase, *ChemCatChem*, **4**, 1167–1172 (2012).

S11 Mutti, F.G.; Fuchs, C.S.; Pressnitz, D.; Turrini, N.G.; Sattler, J.H.; Lerchner, A.; Skerra, A.; Kroutil, W. Amination of ketones by employing two new (S)-selective ω-transaminases and the His-tagged ω-TA from *Vibrio fluvialis*. *Eur. J. Org. Chem.*, **2012**, 1003–1007 (2012).

S12 Midelfort, K. S.; Kumar, R.; Han, S.; Karmilowicz, M. J.; McConnell, K.; Gehlhaar, D. K.; Mistry, A.; Chang, J. S.; Anderson, M.; Villalobos, A.; Minshull, J.; Govindarajan, S.; Wong, J. W. Redesigning and characterizing the substrate specificity and activity of *Vibrio fluvialis* aminotransferase for the synthesis of imagabalin. *Protein Eng. Des. Sel.,* **26**, 25-33 (2013).

S13 Itagaki, N.; Kimura, M.; Sugahara, T.; Iwabuchi, Y. Organocatalytic Entry to Chiral Bicyclo[3.n.1]Alkanones via Direct Asymmetric Intramolecular Aldolization. *Org. Lett.*, **7**, 4185–4188 (2005).

S14 Stocks, P. A.; Bray, P.G.; Barton, V.E.; Al-Helal, M.; Jones, M.; Araujo, N.C..; Gibbons, P.; Ward, S. A.; Hughes, R. H.; Biagini, G. A.; Davies, J. Amewu, R.; Mercer, A. E.; Ellis, G.; O'Neill, P. M. Evidence for a Common Non-Heme Chelatable-Iron-Dependent Activation Mechanism for Semisynthetic and Synthetic Endoperoxide Antimalarial Drugs. *Angew. Chem. Int. Ed.* **46**, 6278-6283 (2007).

S15 Rosowsky, A.; Papoulis, A. T.; Forsch, R. A.; Queener, S. F. Synthesis and antiparasitic and Antitumor of 2,4-Diamino-6-(arylmethyl)-5,6,7,8-tetrahydroquinazoline Analogues of Piritrexim. *J. Med. Chem.* **42**, 1007-1017 (1999).

S16 Falus, P.; Boros, Z.; Hornyánszky, G.; Nagy, J.; Darvas, F.; Ürge, L.; Poppe, L. Reductive Amination of Ketones: Novel One-Step Transfer Hydrogenations in Batch and Continuous-Flow Mode. *Tetrahedron Lett*. **52**, 1310–1312 (2011).

S17 Molnár, Z.; Farkas, E.; Lakó, Á.; Erdélyi, B.; Kroutil, W.; Vértessy, B. G.; Paizs, C.; Poppe, L. Immobilized Whole-Cell Transaminase Biocatalysts for Continuous-Flow Kinetic Resolution of Amines. *Catalysts* **9** (5), 438 (2019).

S18 Cassimjee K. E.; Humble M. S.; Miceli V.; Colomina C. G.; Berglund P. Active Site Quantification of an ω-Transaminase by Performing a Half Transamination Reaction. *ACS Catal.* **1**, 1051-1055 (2011).

S19 Abaházi, E.; Sátorhelyi, P.; Erdélyi, B.; Vértessy, B. G.; Land, H.; Paizs, C.; Berglund, P.; Poppe, L.: Covalently immobilized Trp60Cys mutant of ω-transaminase from *Chromobacterium violaceum* for kinetic resolution of racemic amines in batch and continuous-flow modes. *Biochem. Eng. J.*, **132**, 270-278 (2018).

S20 Cassimjee K. E.; Humble M. S.; Miceli V.; Colomina C. G.; Berglund P. Active Site Quantification of an ω-Transaminase by Performing a Half Transamination Reaction. *ACS Catal.* **1**, 1051-1055 (2011).

S21 Chen, S.; Land, H.; Berglund, P.; Humble, M. S. Stabilization of an Amine Transaminase for Biocatalysis. *J. Mol. Catal. B Enzym*. **124**, 20–28 (2016).

S22 Máthé, T.; Hegedűs, L.; Czibula, L.; Juhász, B.; Nagyné Bágdy, J. M. D. (Gedeon Richter Ltd.) Process for the Preparation of trans 4-Amino-Cyclohexyl Acetic Acid Ethyl Ester HCl, *WIPO Pat. Appl.*, WO 2010/070368 A1 (2010).
