# Supplementary material for: Transaminase-catalysis to produce trans-4-substituted cyclohexane-1-amines including a key intermediate towards cariprazine
Source: Commun Chem. 2024 Apr 18;7:86. doi: 10.1038/s42004-024-01148-9 (PMC11026398; doi:10.1038/s42004-024-01148-9)
Supplement: Supplementary file 3 — Description of Additional Supplementary Files [file 42004_2024_1148_MOESM3_ESM.pdf]

Description of Additional Supplementary Files

File name: Supplementary Data 1

Description: NMR spectra of the synthesized substrates and products.

File name: Supplementary Data 2

Description: GC chromatograms of reactions and products.

File name: Supplementary Data 3

Description: IR spectra of synthesized substrates and intermediates.

File name: Supplementary Data 4

Description: Source data for Figure 2.

File name: Supplementary Data 5

Description: Source data for Figure 3.

File name: Supplementary Data 6

Description: Data and calculations for Table 1

File name: Supplementary Data 7

Description: Source data for Section 3.4.3 in Supplementary Information and FigS15.
