# Supplementary material for: Transaminase-catalysis to produce trans-4-substituted cyclohexane-1-amines including a key intermediate towards cariprazine
Source: Commun Chem. 2024 Apr 18;7:86. doi: 10.1038/s42004-024-01148-9 (PMC11026398; doi:10.1038/s42004-024-01148-9)
Supplement: Supplementary file 4 — Supplementary Data 1 [file 42004_2024_1148_MOESM4_ESM.pdf]

The NMR spectra of the synthesized substrates and products

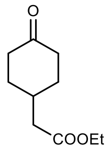

2a

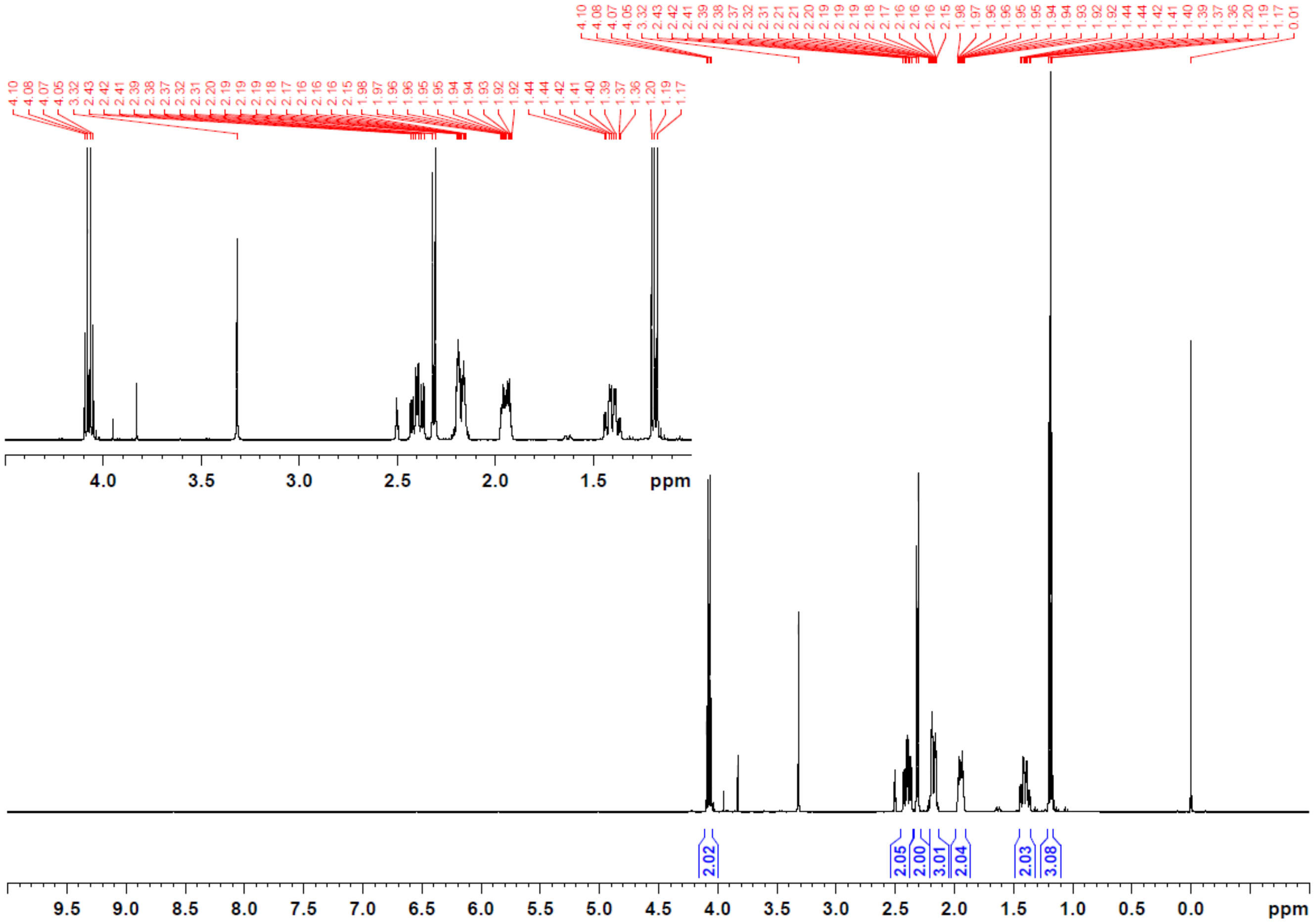

Figure NMR1 ^{1}H-NMR (500 MHz, DMSO-d_6) spectrum of ethyl-2-(4-oxocyclohexyl)acetate **2a**

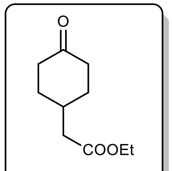

2a

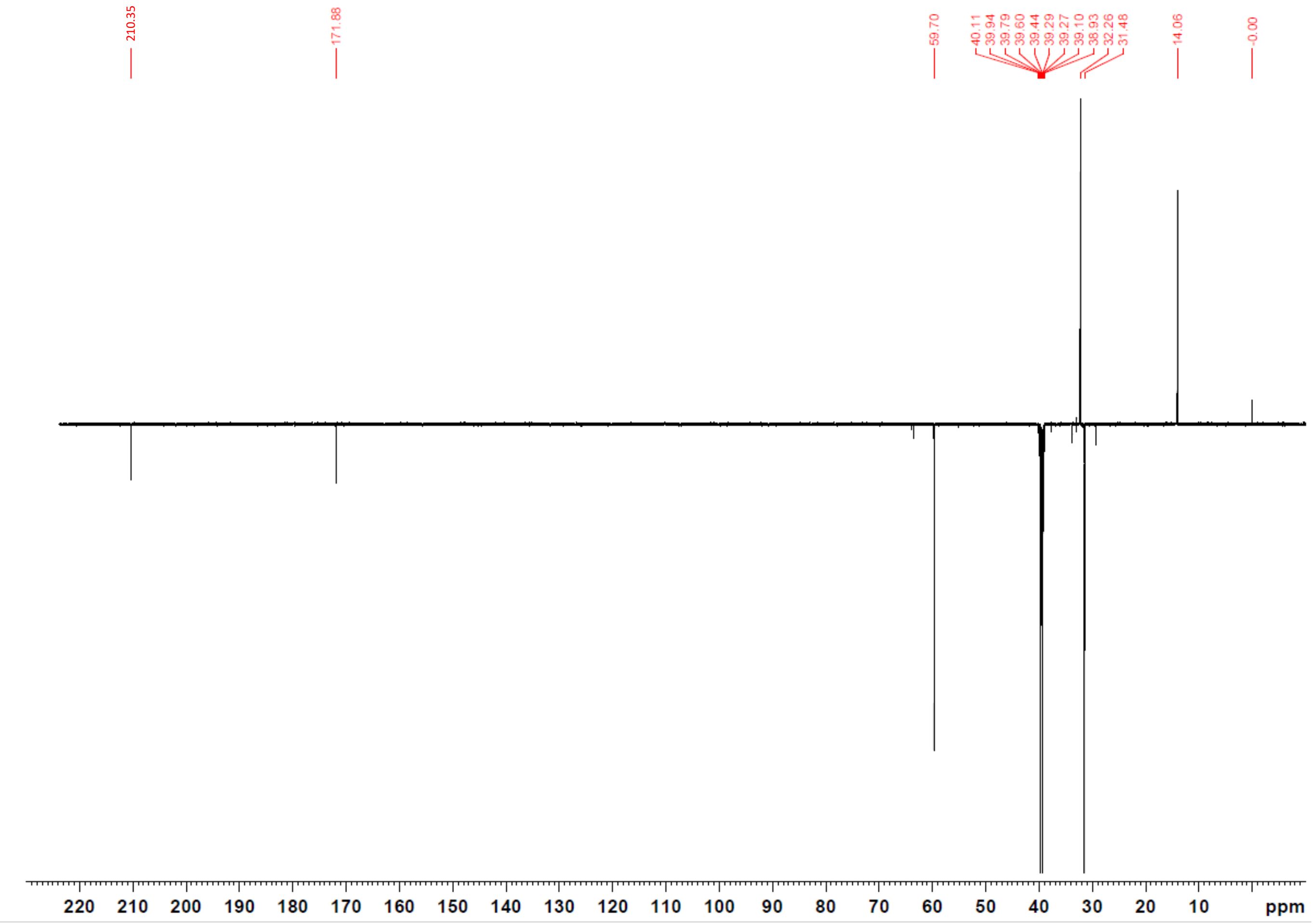

Figure NMR2 DEPTQ-NMR (125 MHz, DMSO-d_6) spectrum of ethyl-2-(4-oxocyclohexyl)acetate **2a**

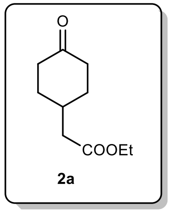

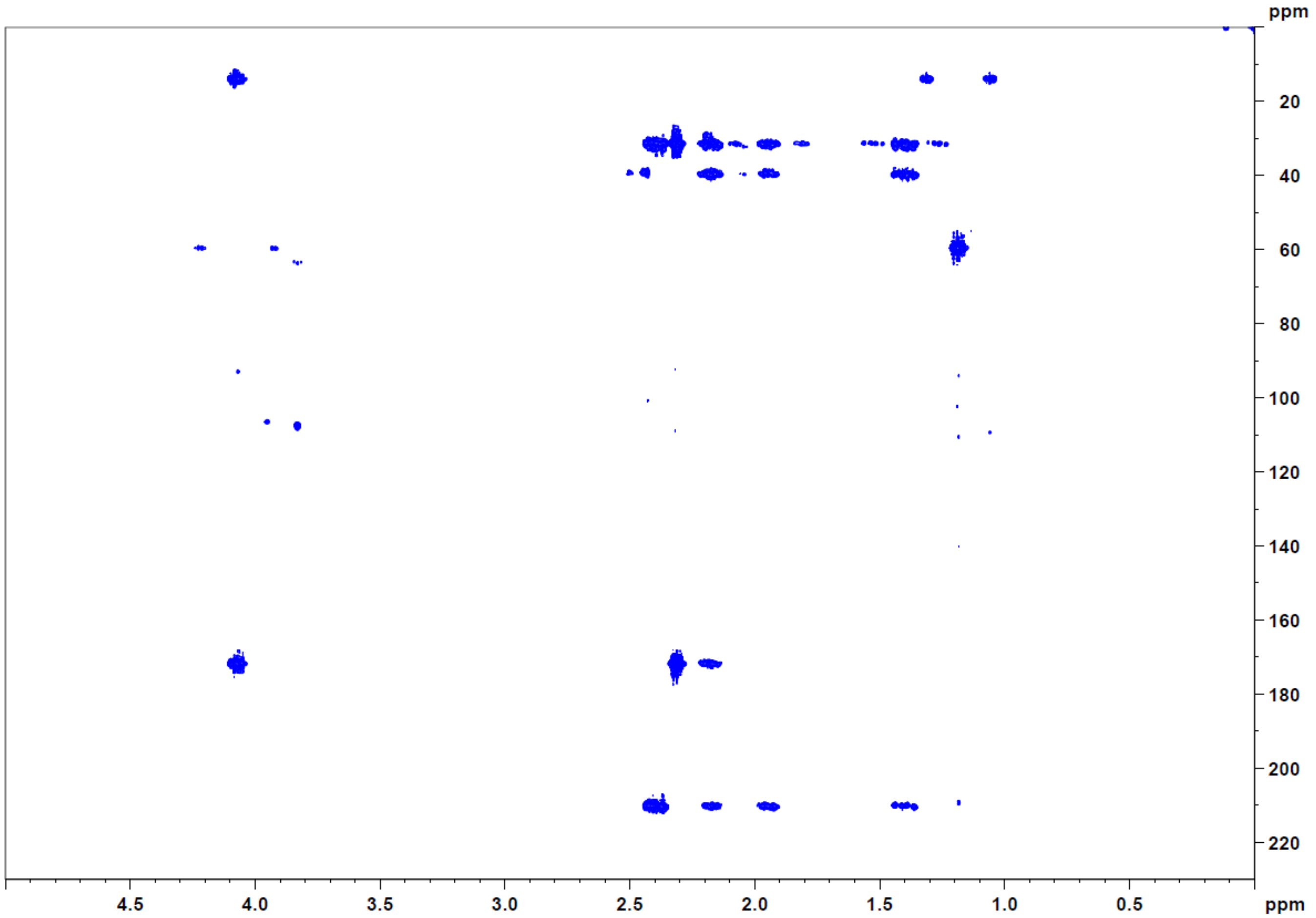

Figure NMR3 HMBC spectrum of ethyl-2-(4-oxocyclohexyl)acetate **2a**

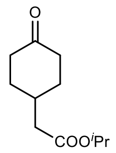

2b

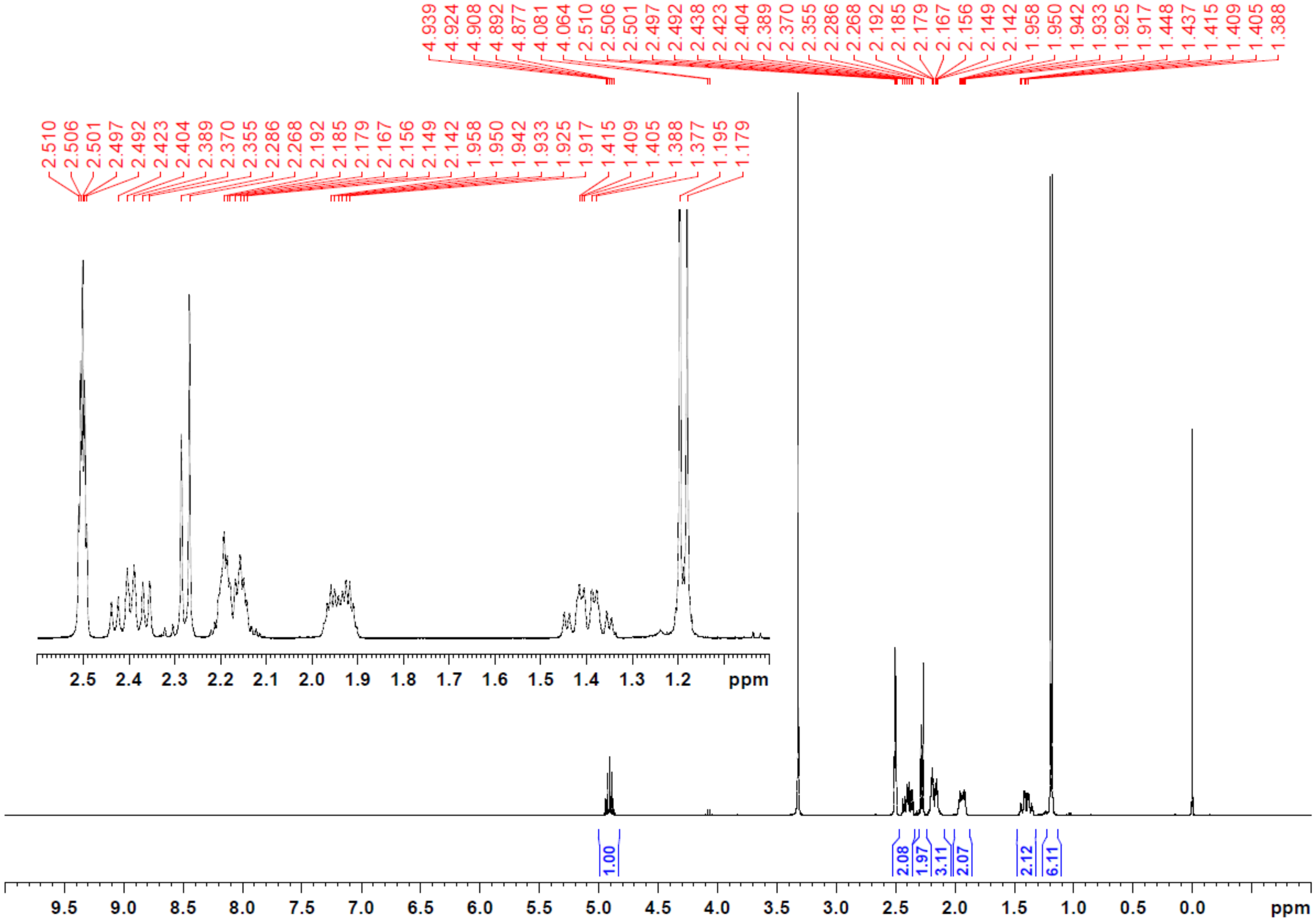

Figure NMR4 ^{1}H-NMR (500 MHz, DMSO-d_6) spectrum of isopropyl-2-(4-oxocyclohexyl)acetate **2b.**

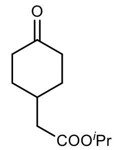
2b

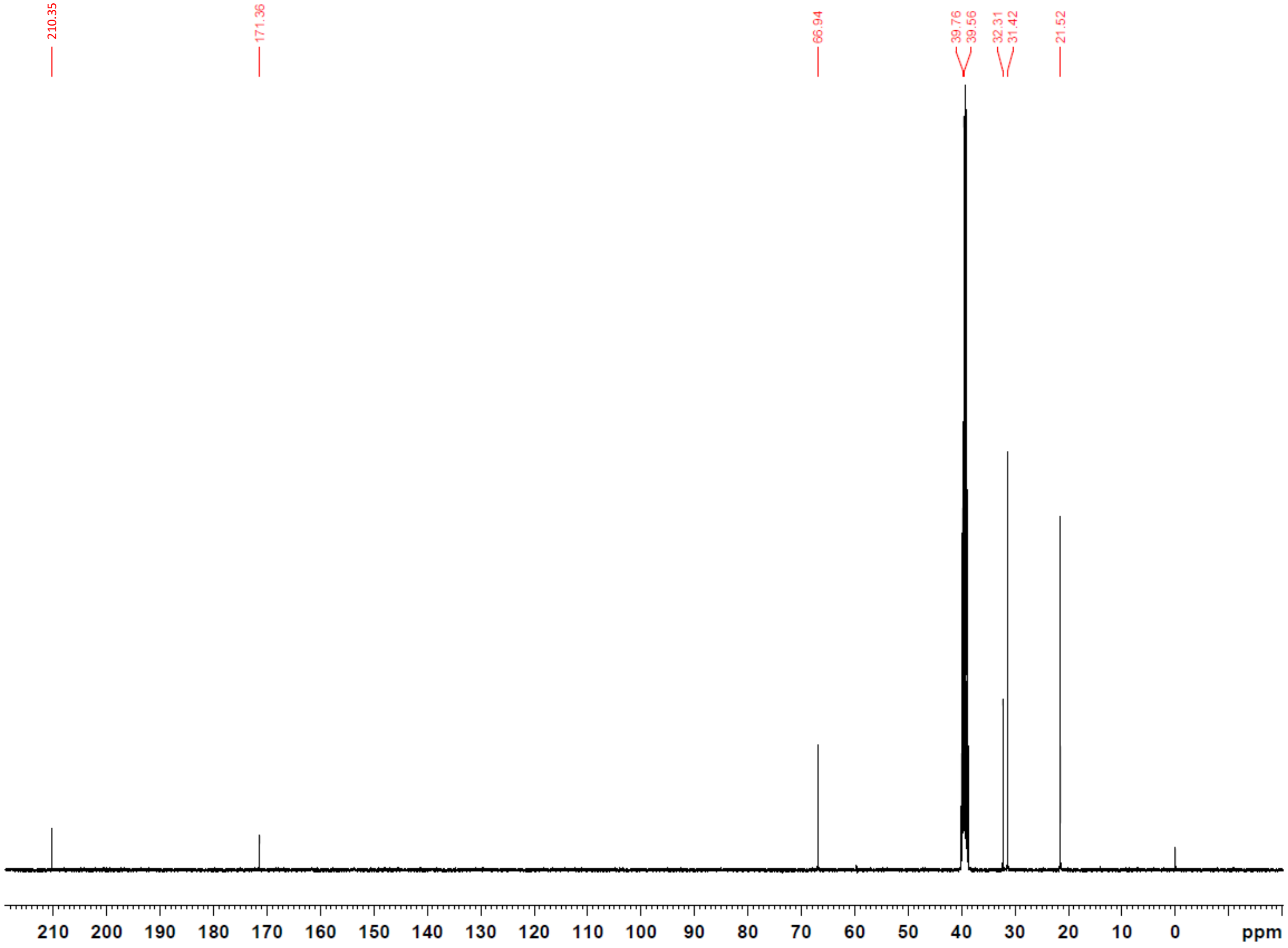

Figure NMR5 DEPTQ -NMR (125 MHz, DMSO-d_6) spectrum of isopropyl-2-(4-oxocyclohexyl)acetate **2b.**

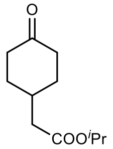

2b

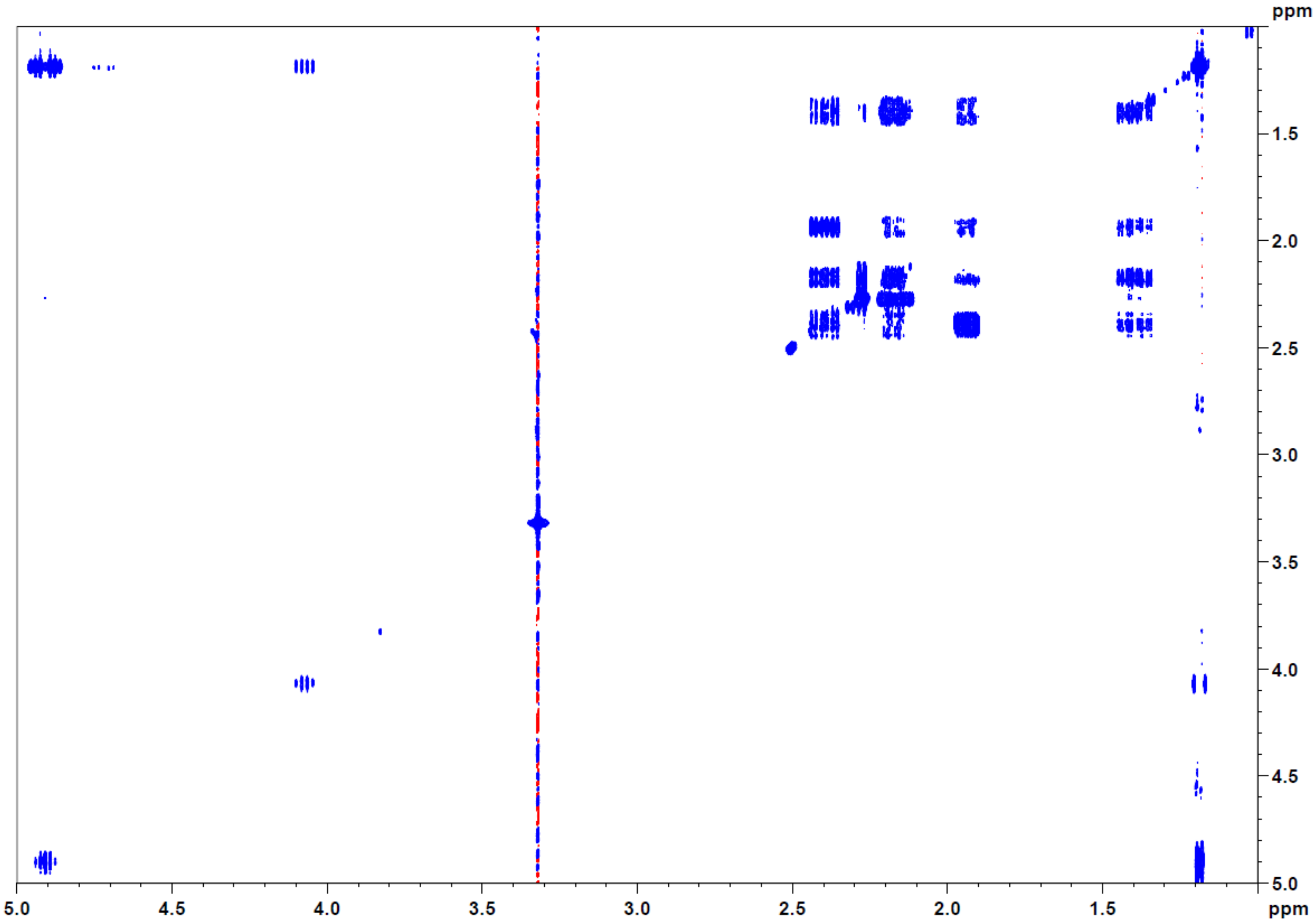

Figure NMR6 COSY spectrum of isopropyl-2-(4-oxocyclohexyl)acetate **2b.**

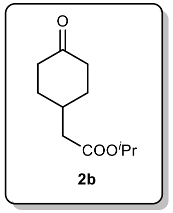

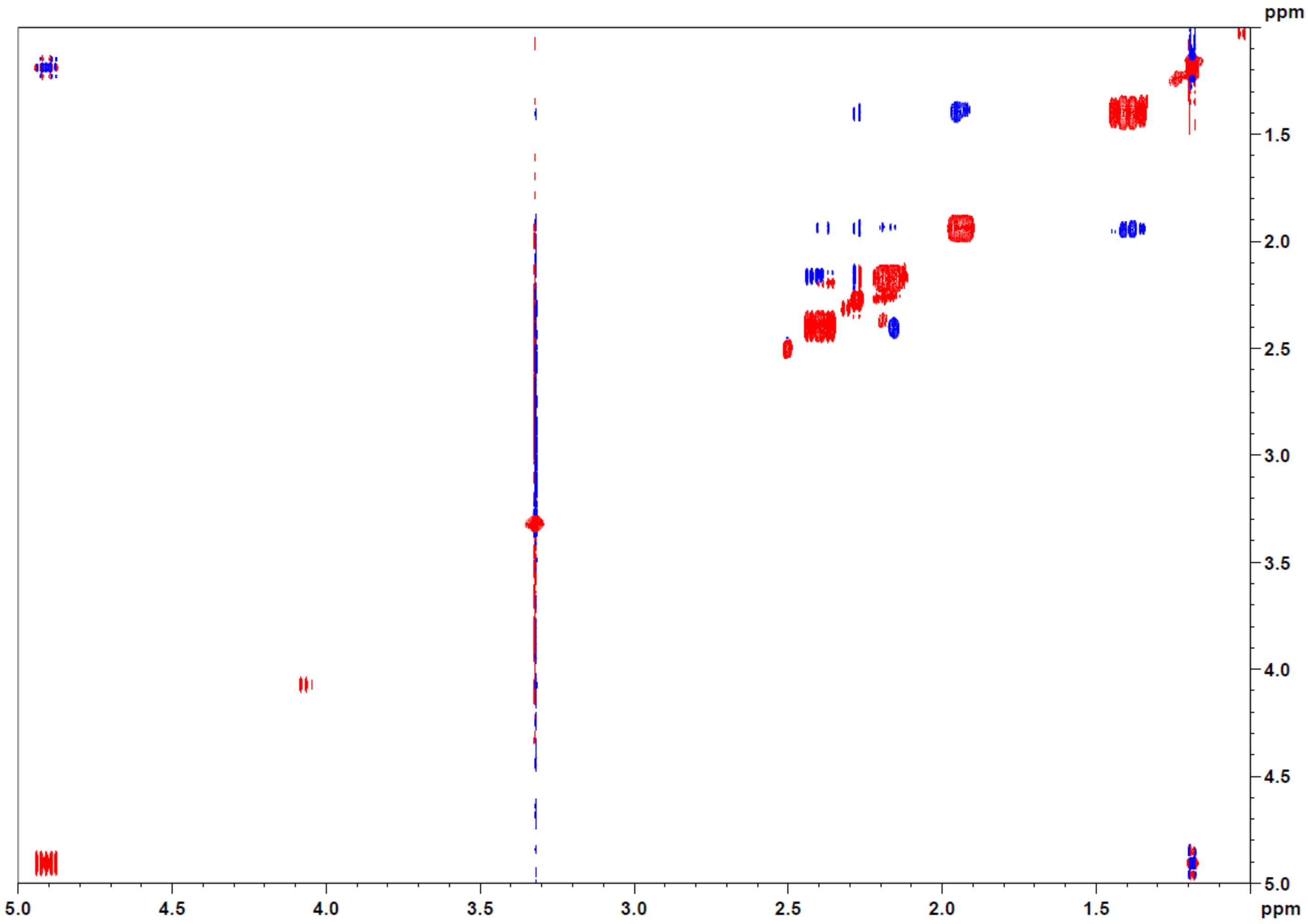

Figure NMR7 NOESY spectrum of isopropyl-2-(4-oxocyclohexyl)acetate **2b.**

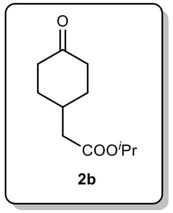

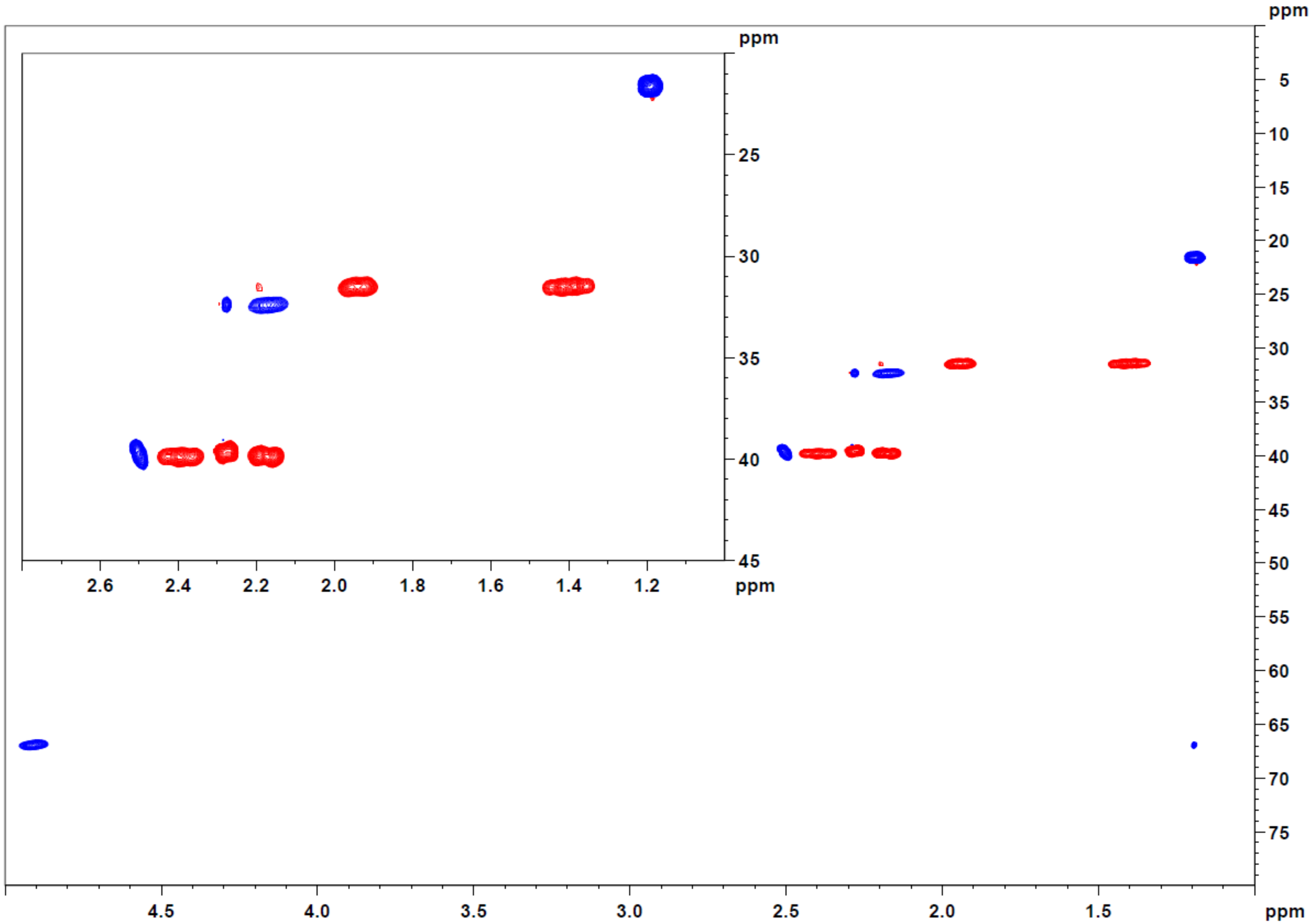

Figure NMR8 HSQC spectrum of isopropyl-2-(4-oxocyclohexyl)acetate **2b.**

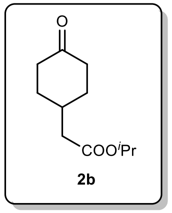

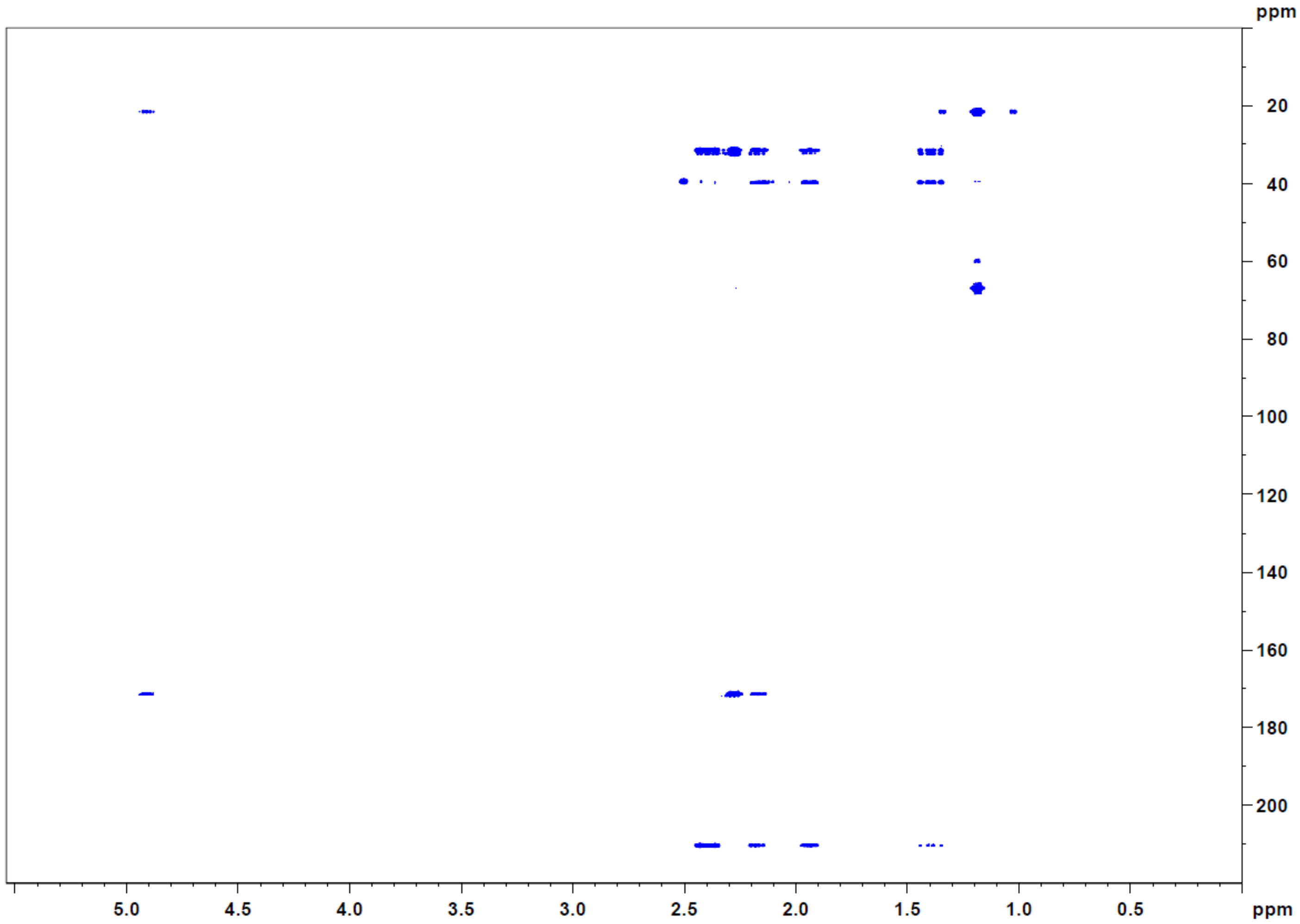

Figure NMR9 HMBC spectrum of isopropyl-2-(4-oxocyclohexyl)acetate **2b.**

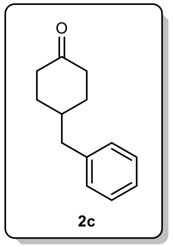

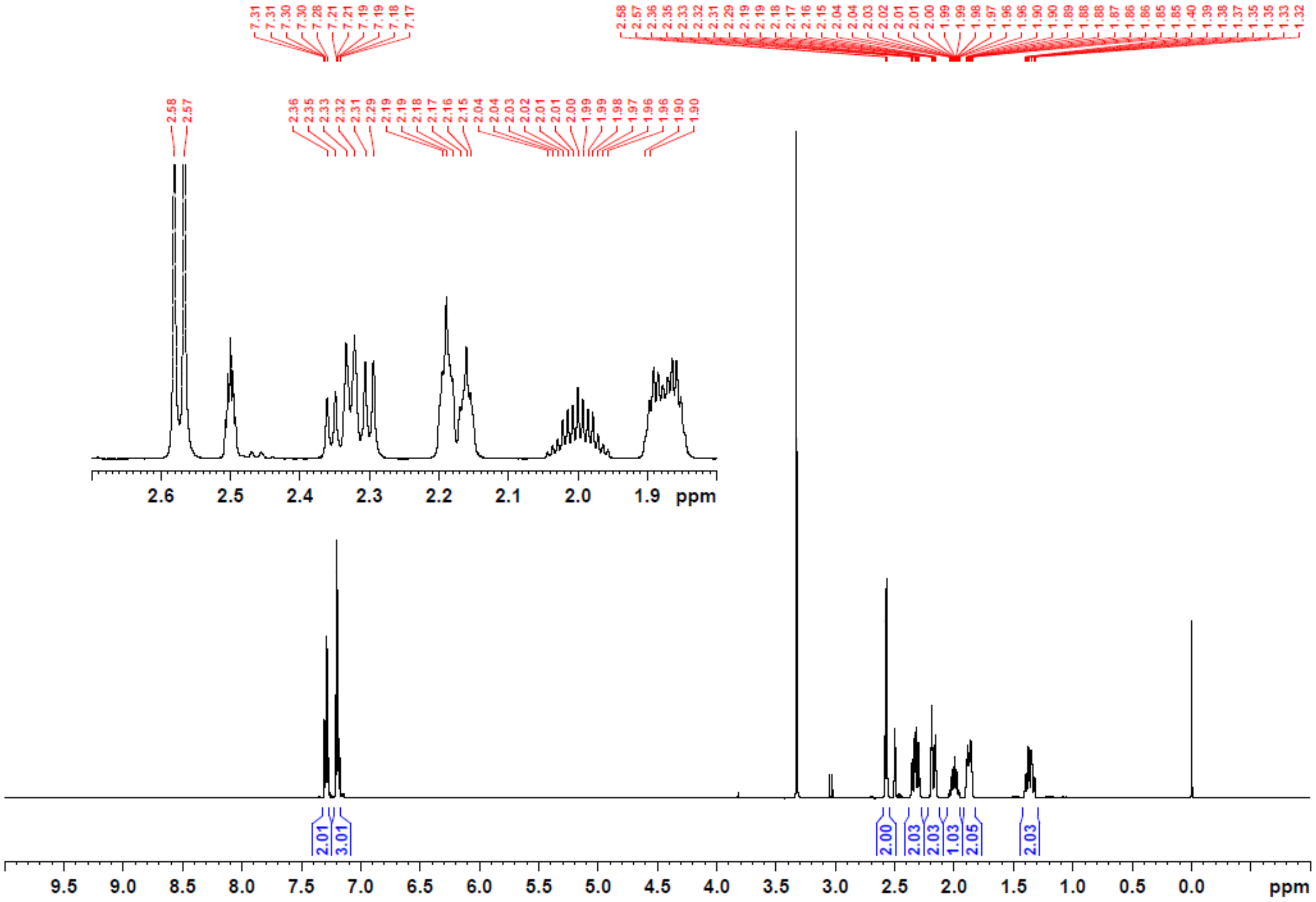

Figure NMR10 ^{1}H-NMR (500 MHz, DMSO-d_6) spectrum of 4-benzylcyclohexyl-1-one **2c.**

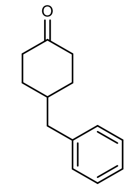

2c

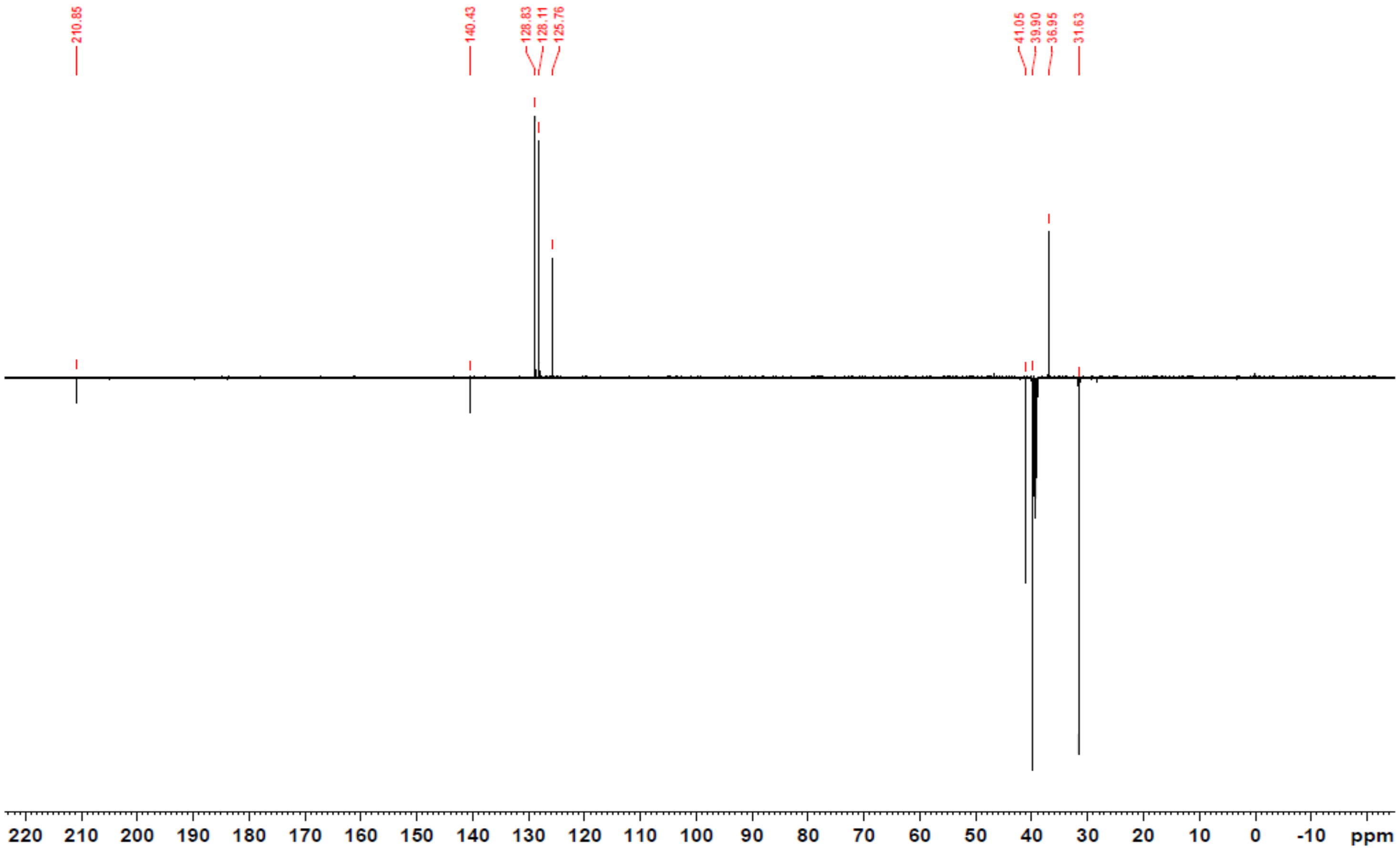

Figure NMR11 DEPTQ -NMR (125 MHz, DMSO-d_6) spectrum of 4-benzylcyclohexyl-1-one **2c.**

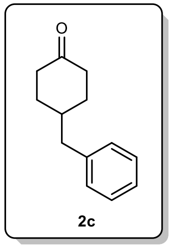

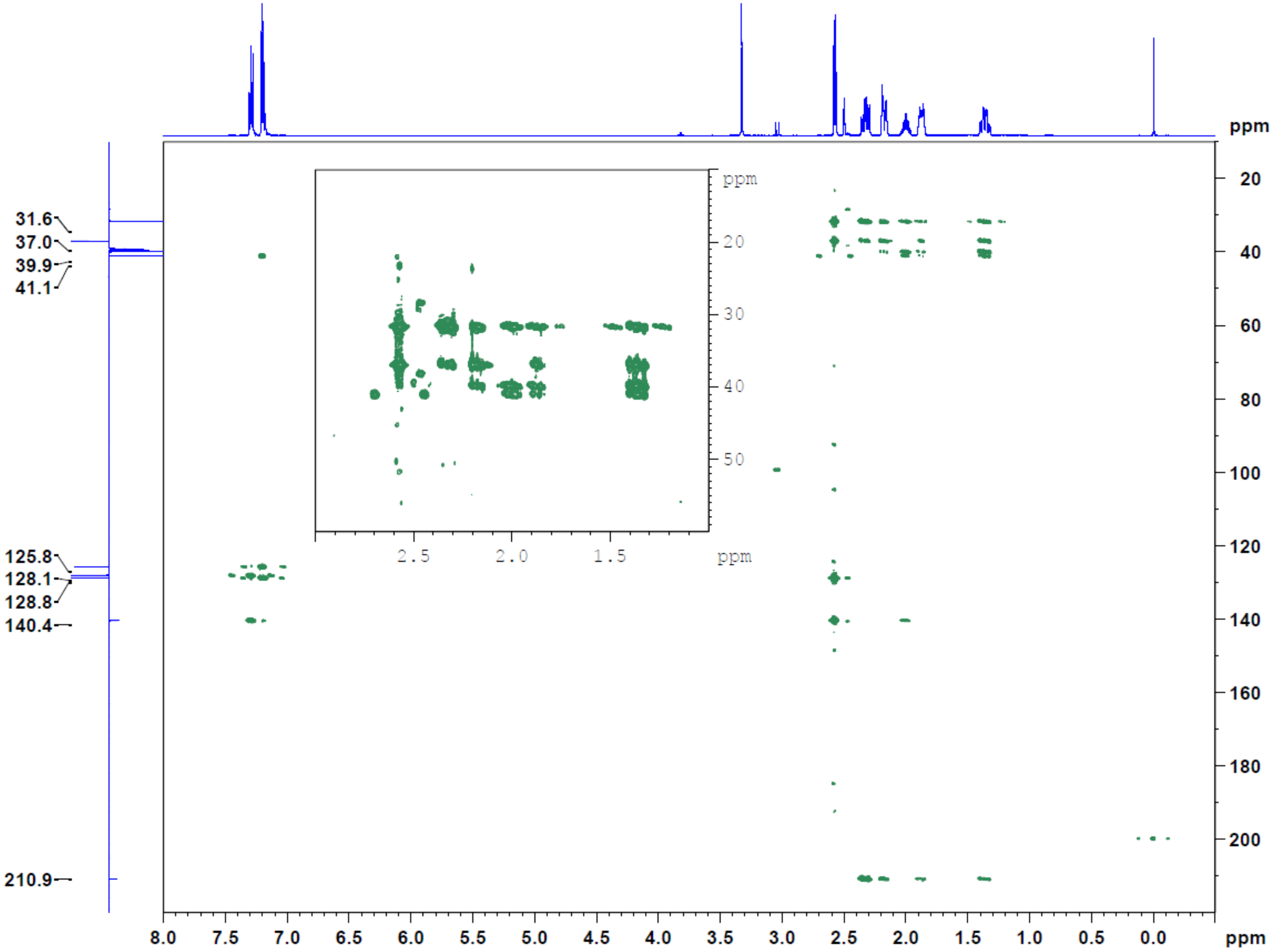

Figure NMR12 HMBC spectrum of 4-benzylcyclohexyl-1-one **2c.**

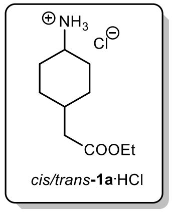

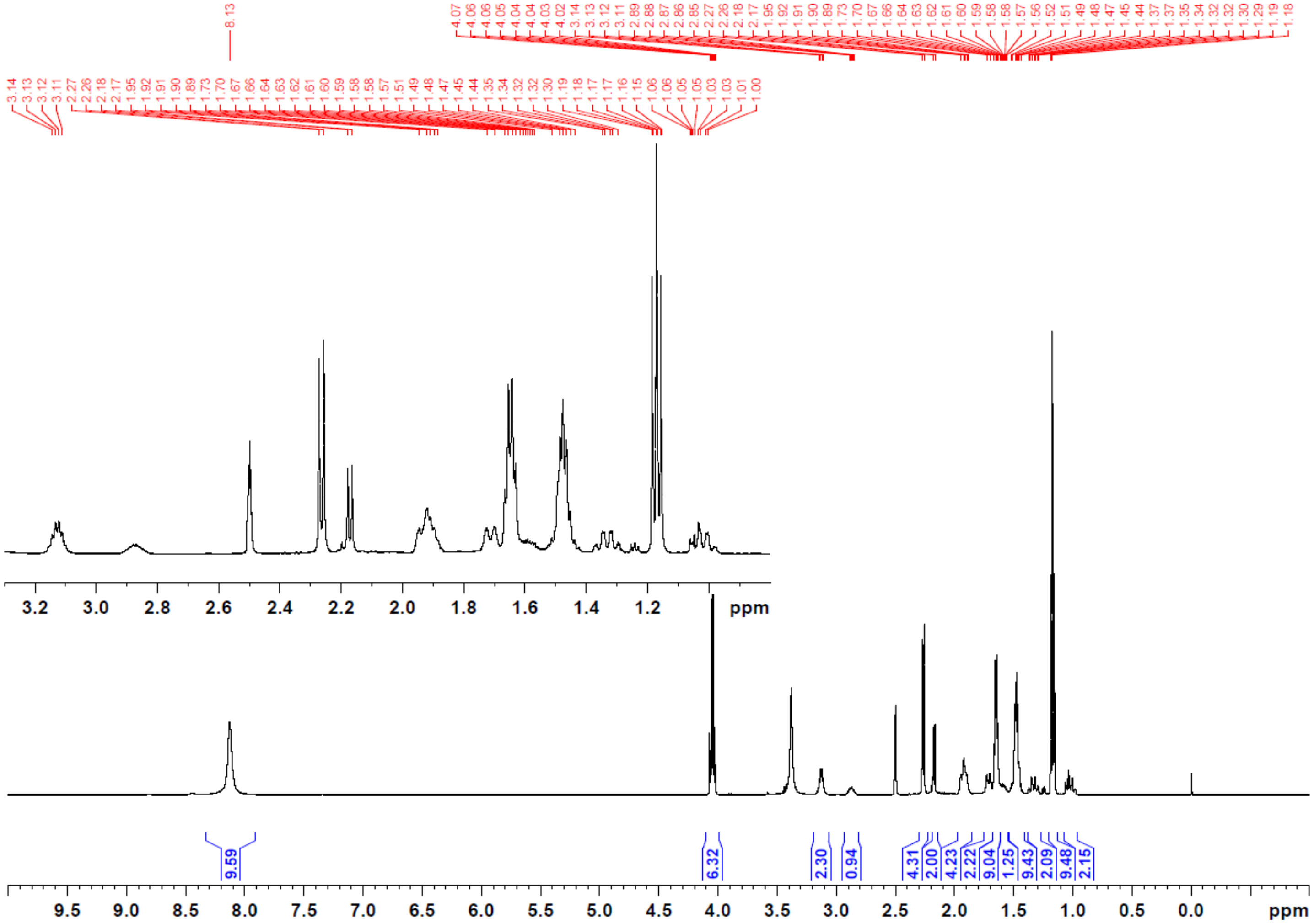

Figure NMR13 ^{1}H-NMR (500 MHz, DMSO-d_6) spectrum of *cis*/*trans*-4-(2-ethoxy-2-oxoethyl)cyclohexan-1-aminium chloride (*cis*/*trans*-**1a·HCl**)

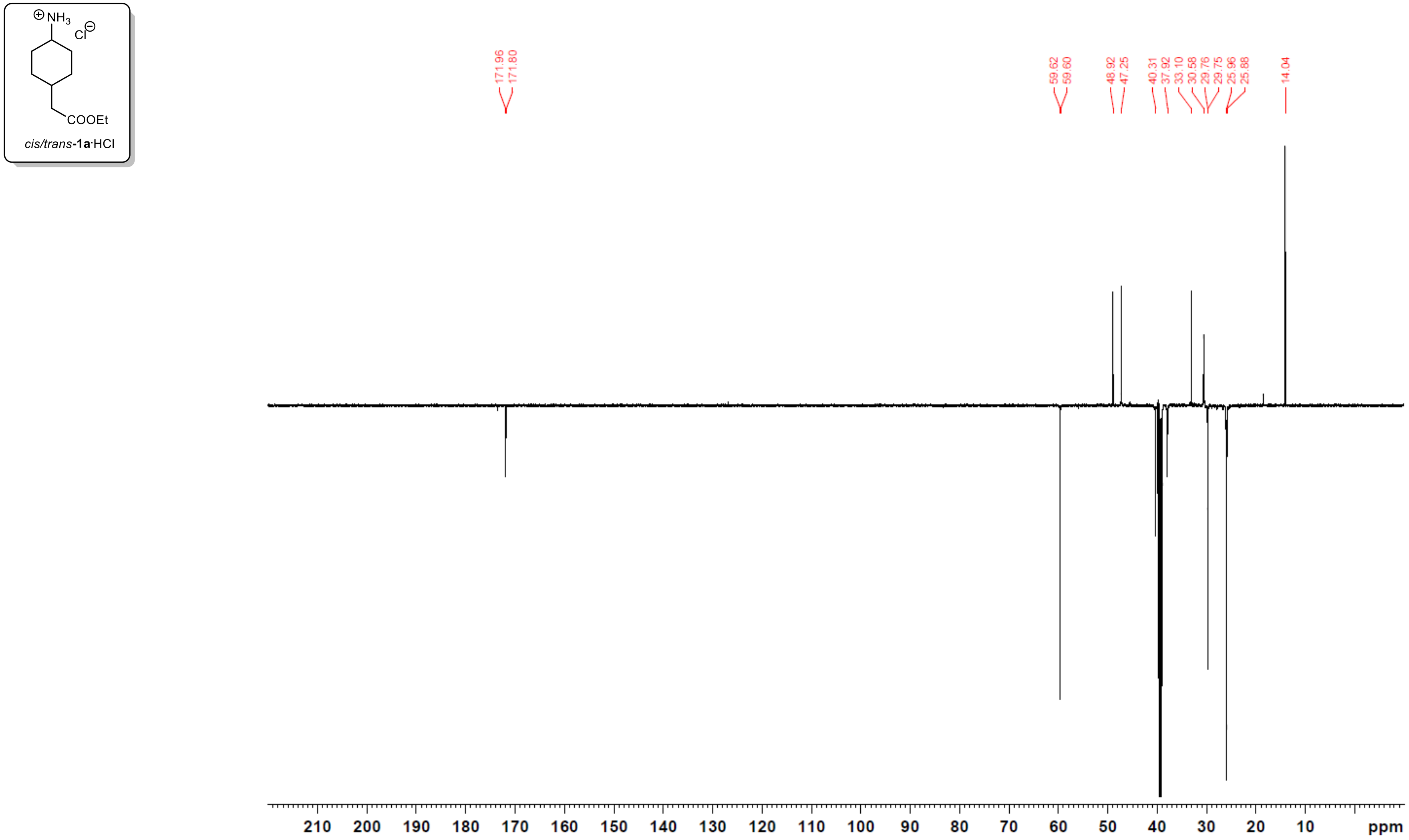

Figure NMR14 DEPTQ -NMR (125 MHz, DMSO-d_6) spectrum of *cis/trans*-4-(2-ethoxy-2-oxoethyl)cyclohexan-1-aminium chloride (*cis/trans*-**1a·HCl**)

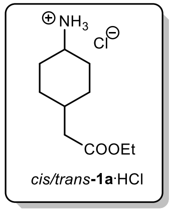

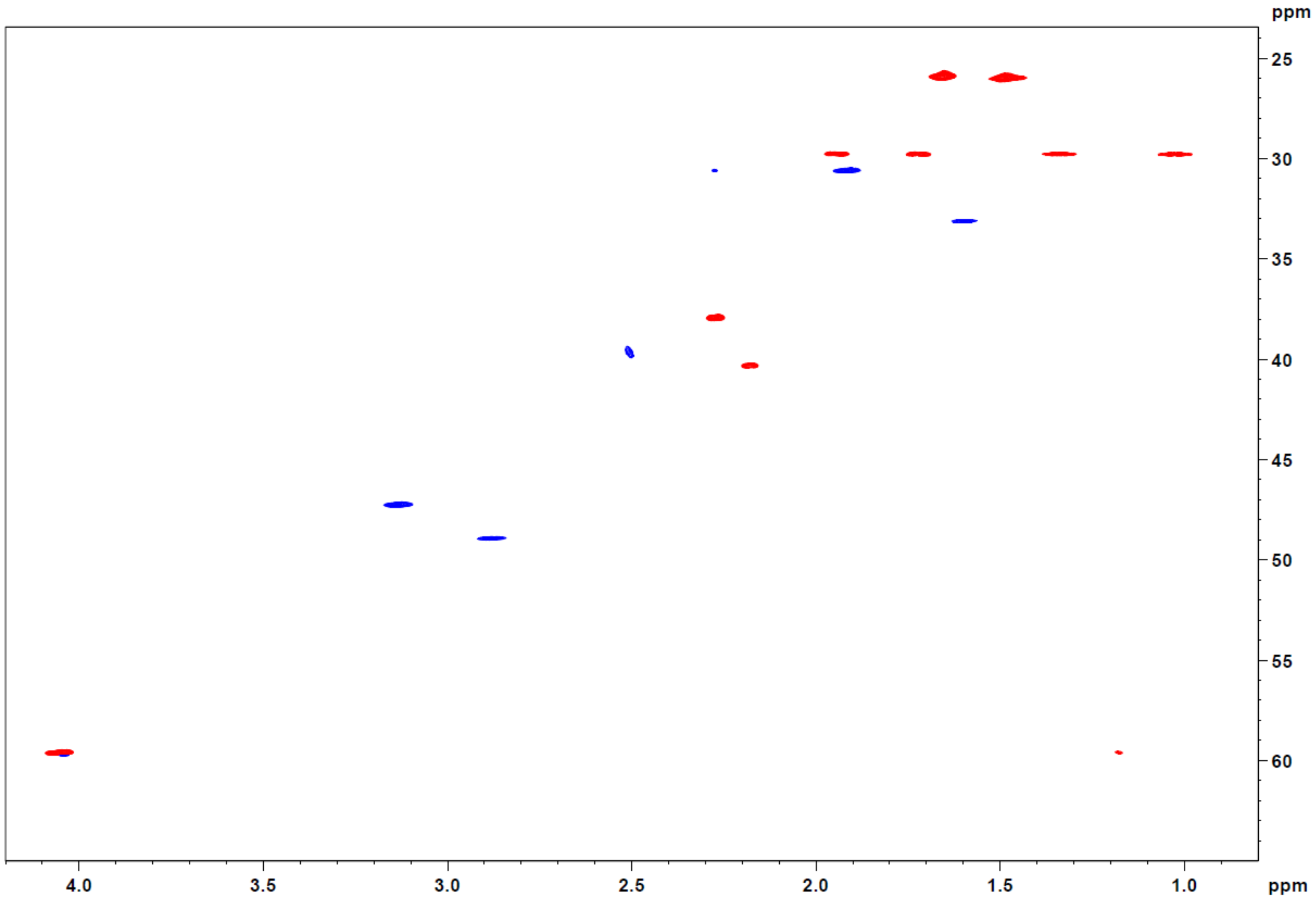

Figure NMR15 HSQC spectrum of *cis*/*trans*-4-(2-ethoxy-2-oxoethyl)cyclohexan-1-aminium chloride (*cis*/*trans*-**1a·HCl**)

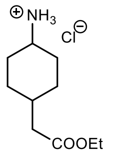

cis/trans-**1a**·HCl

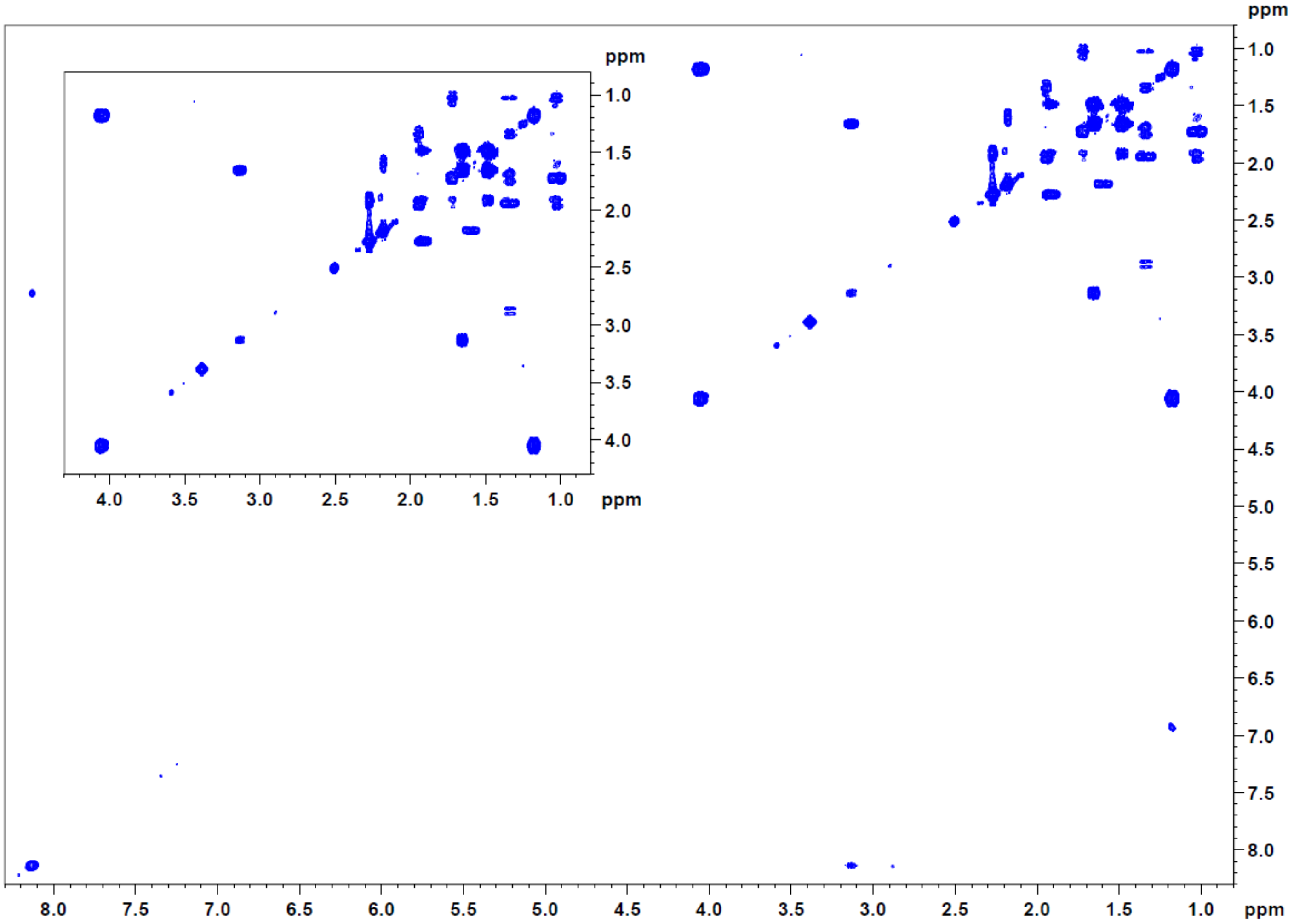

Figure NMR16 HSQC spectrum of *cis*/*trans*-4-(2-ethoxy-2-oxoethyl)cyclohexan-1-aminium chloride (*cis*/*trans*-**1a·HCl**)

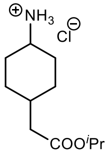

cis/trans-**1b**·HCl

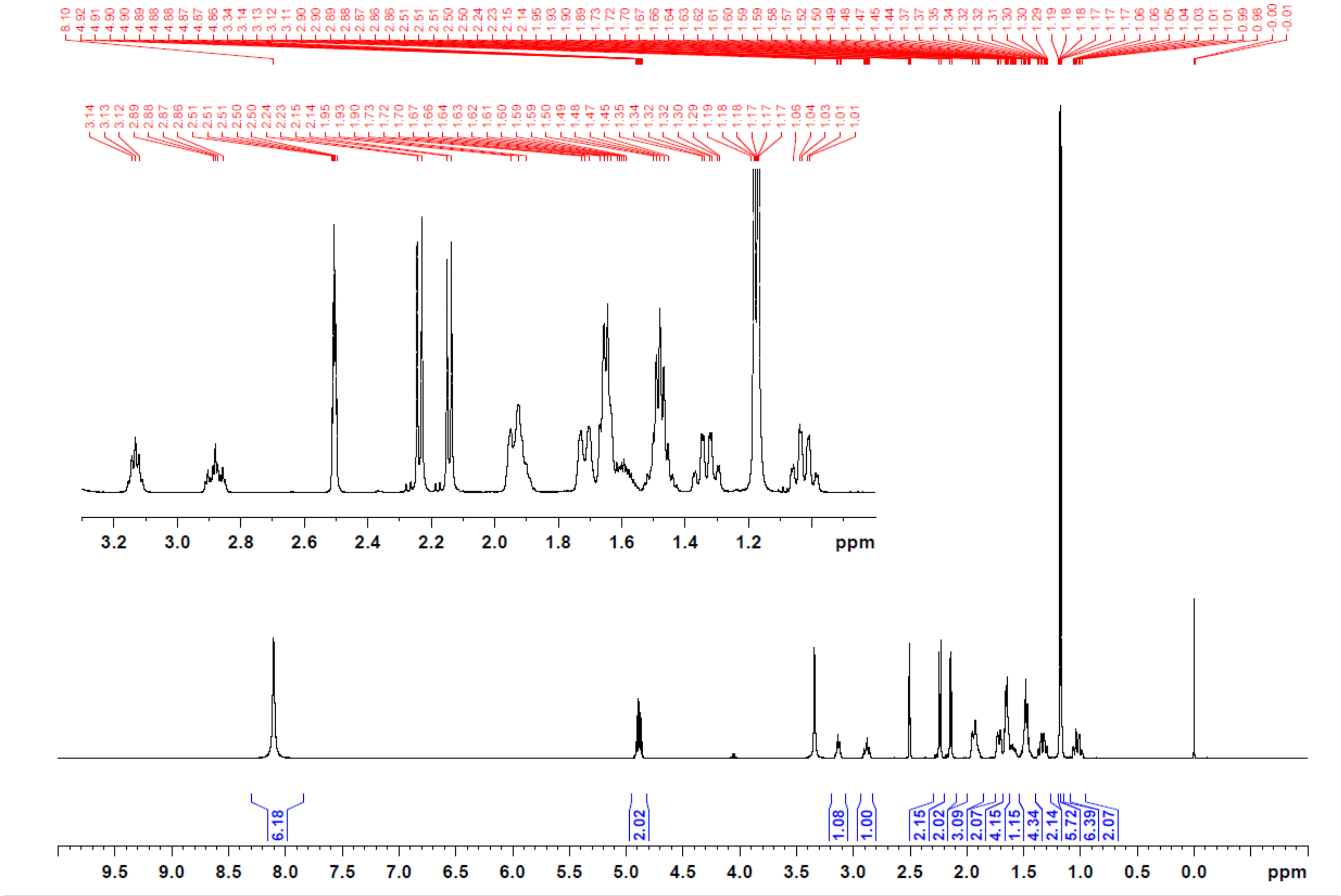

Figure NMR17 ^{1}H-NMR (500 MHz, DMSO-d_6) spectrum of *cis/trans*-4-(2-ethoxy-2-oxoethyl)cyclohexan-1-aminium chloride (*cis/trans*-**1b·HCl**)

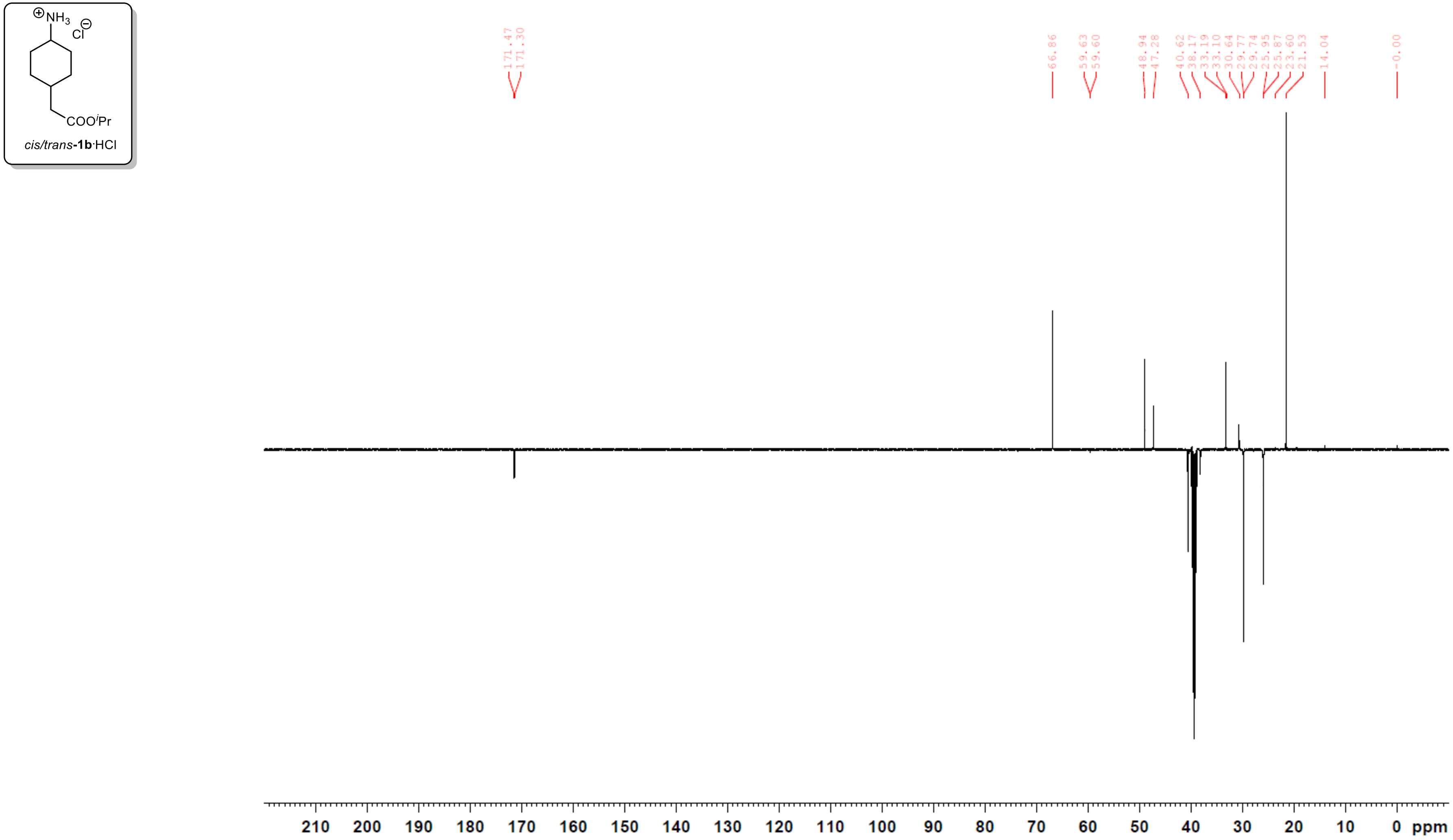

Figure NMR18 DEPTQ -NMR (125 MHz, DMSO-d_6) spectrum of *cis*/*trans*-4-(2-ethoxy-2-oxoethyl)cyclohexan-1-aminium chloride (*cis*/*trans*-**1b·HCl**)

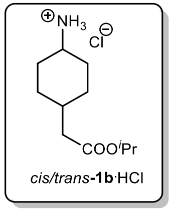

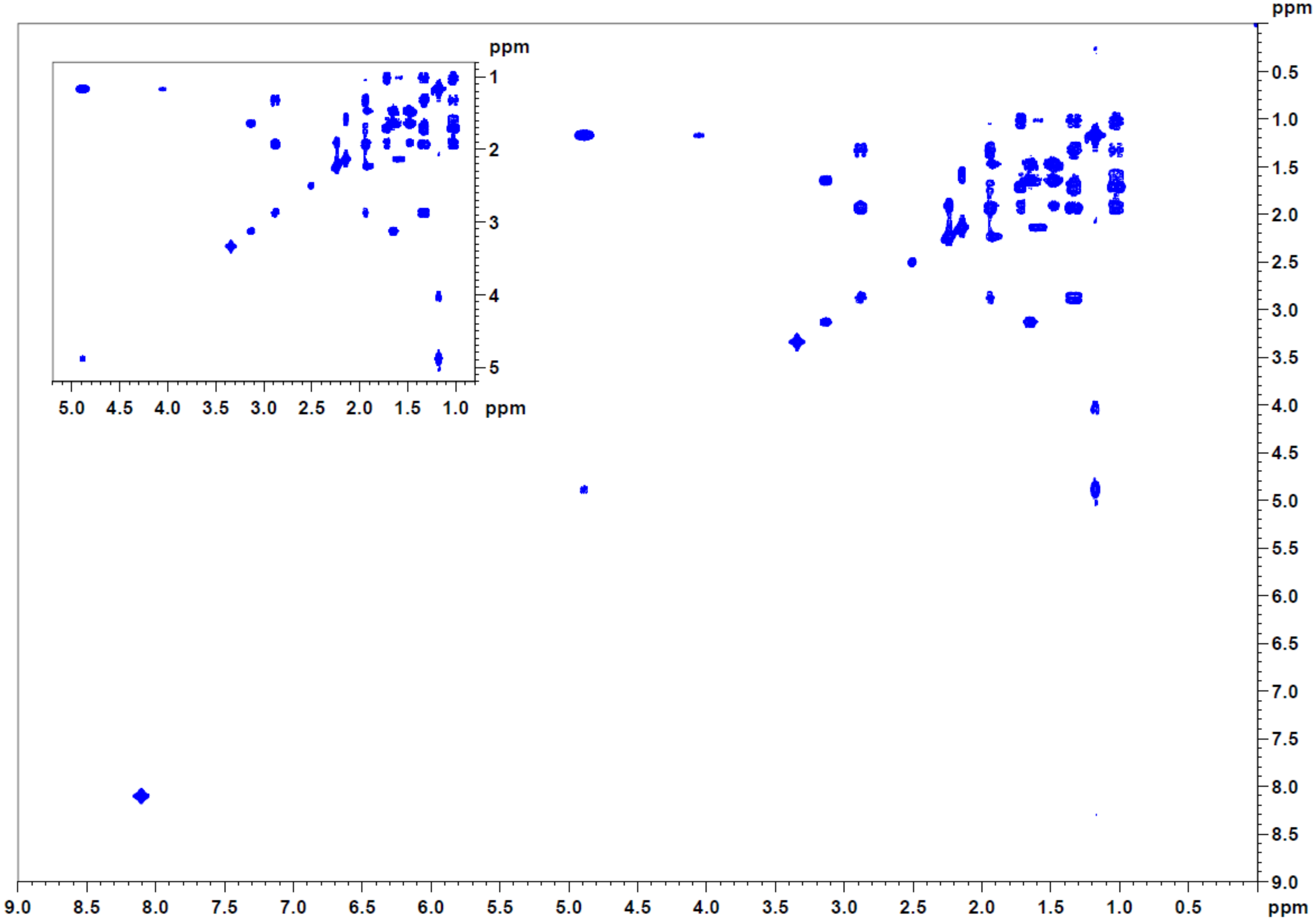

Figure NMR19 COSY spectrum of *cis*/*trans*-4-(2-ethoxy-2-oxoethyl)cyclohexan-1-aminium chloride (*cis*/*trans*-**1b**·HCl)

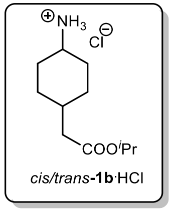

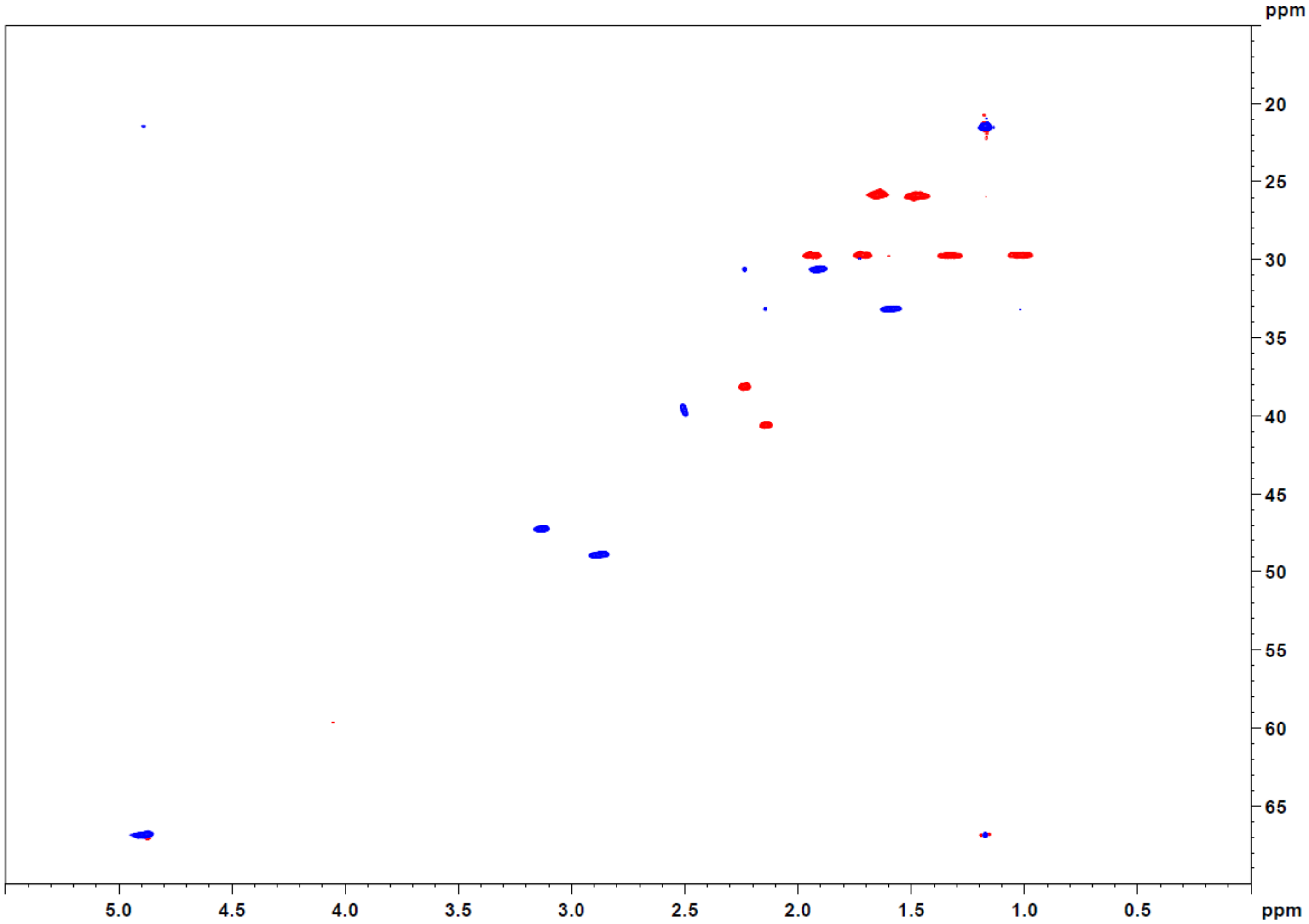

Figure NMR20 HSQC spectrum of *cis*/*trans*-4-(2-ethoxy-2-oxoethyl)cyclohexan-1-aminium chloride (*cis*/*trans*-**1b**·HCl)

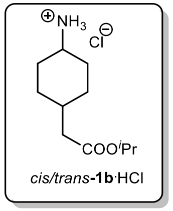

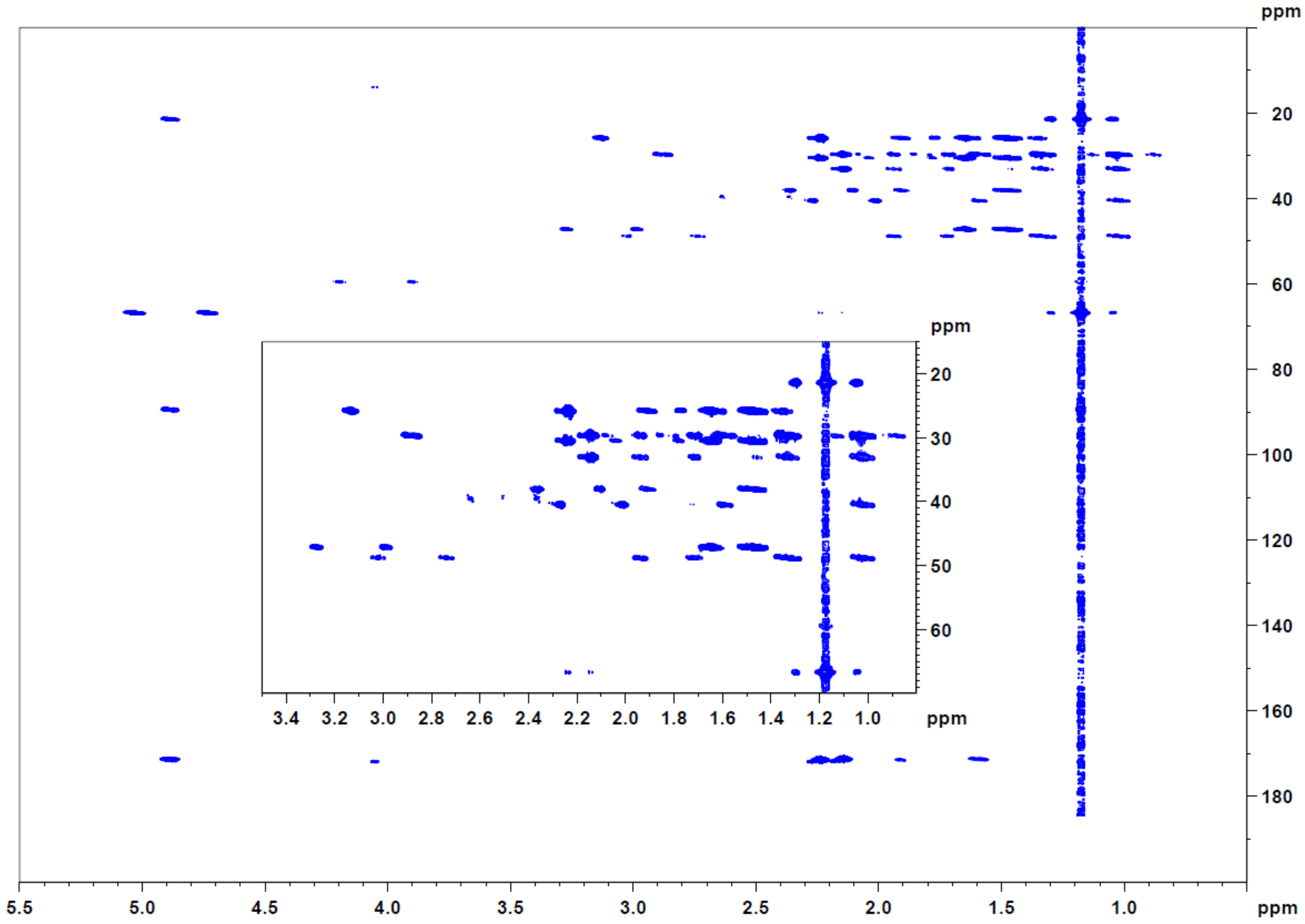

Figure NMR21 HMBC spectrum of *cis*/*trans*-4-(2-isopropoxy-2-oxoethyl)cyclohexan-1-aminium chloride (*cis*/*trans*-**1b**·HCl).

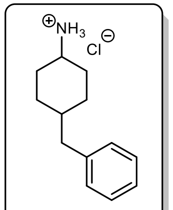

cis/trans-**1c**·HCl

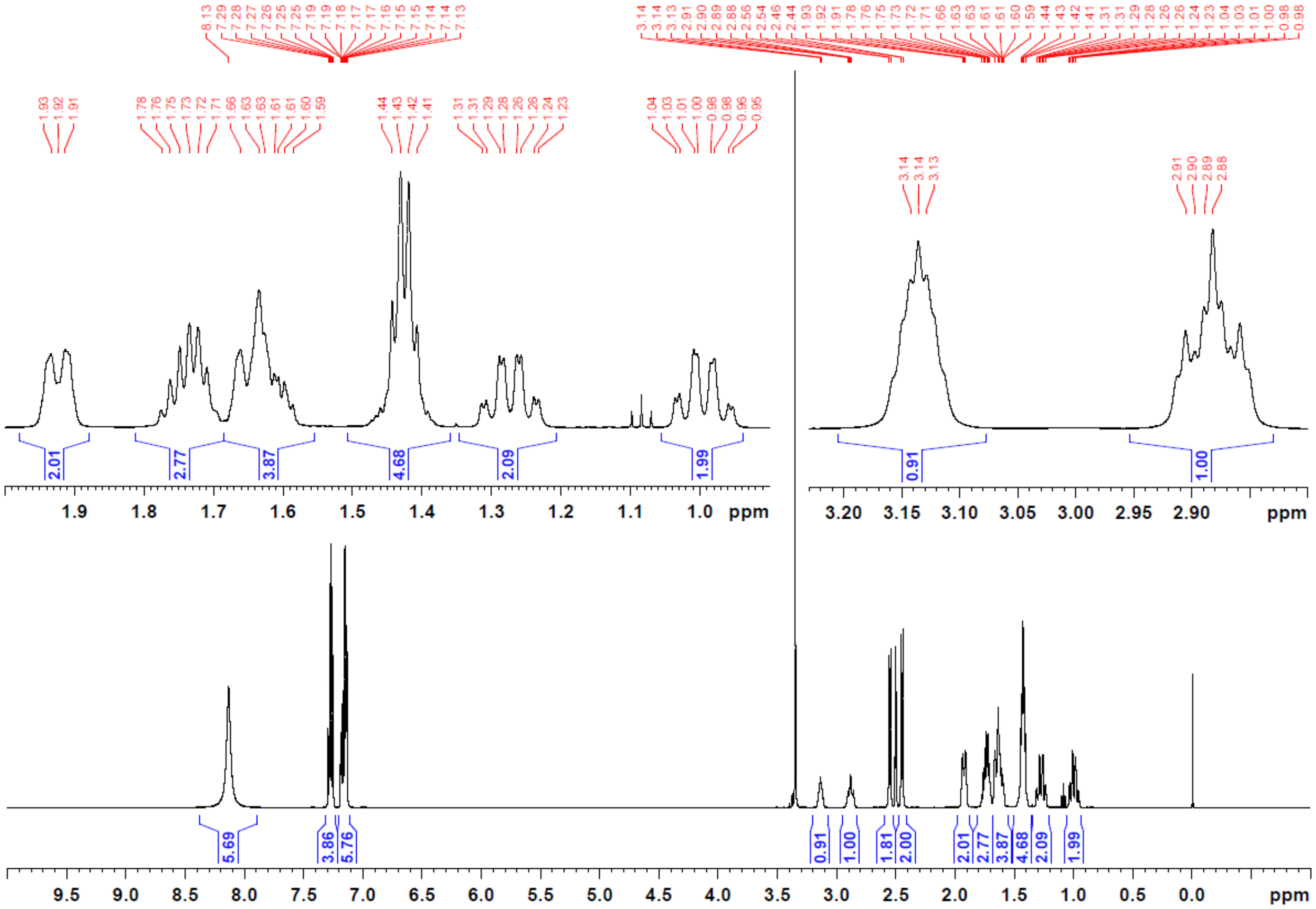

Figure NMR22 ^{1}H-NMR (500 MHz, DMSO-d_6) spectrum of *cis/trans*-4-benzylcyclohexan-1-aminium chloride (*cis/trans*-**1c**·HCl)

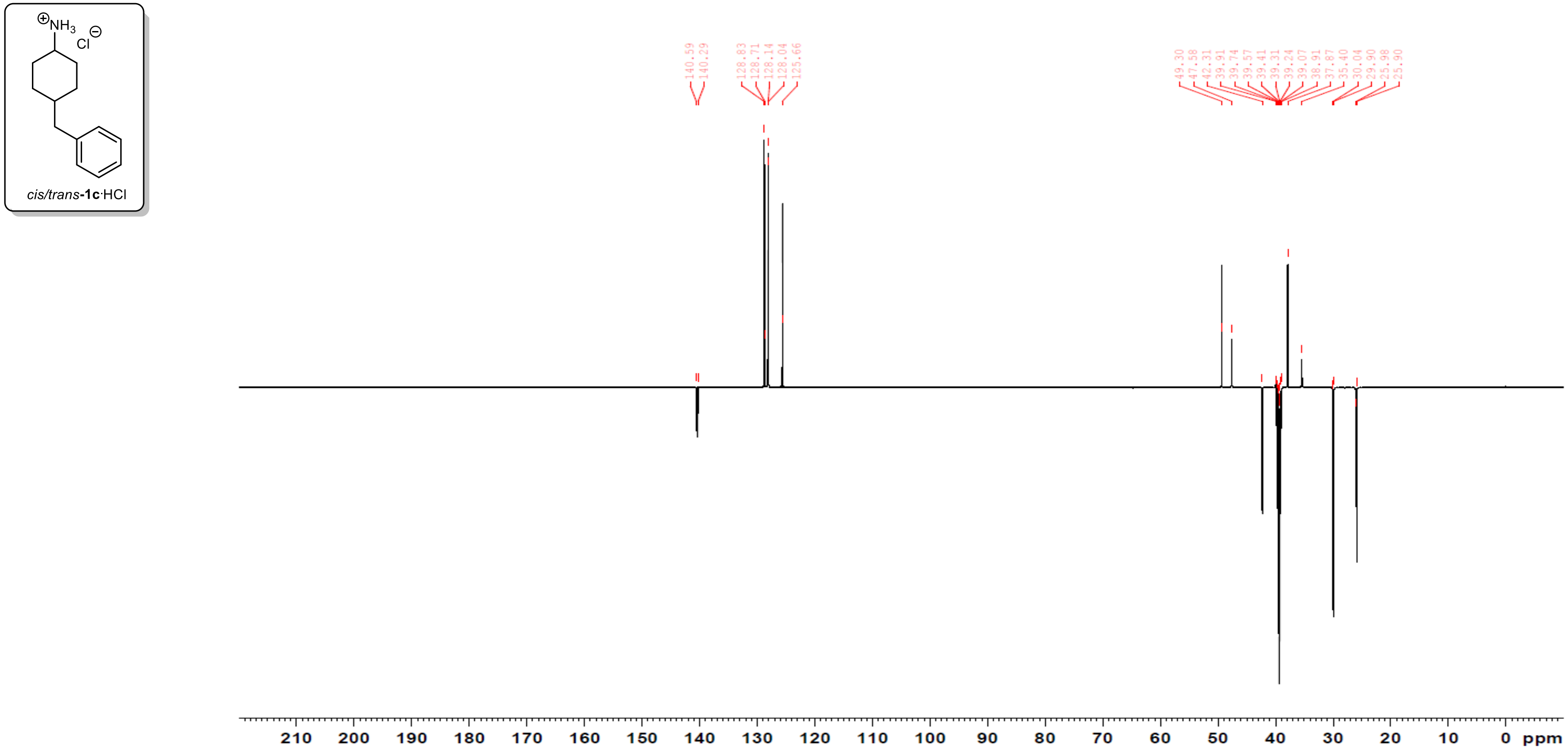

Figure NMR23 DEPTQ -NMR (126 MHz, DMSO-d_6) spectrum of *cis*/*trans*-4-benzylcyclohexan-1-aminium chloride (*cis*/*trans*-**1c**·HCl)

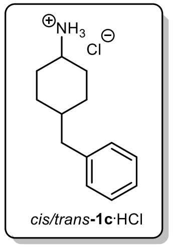

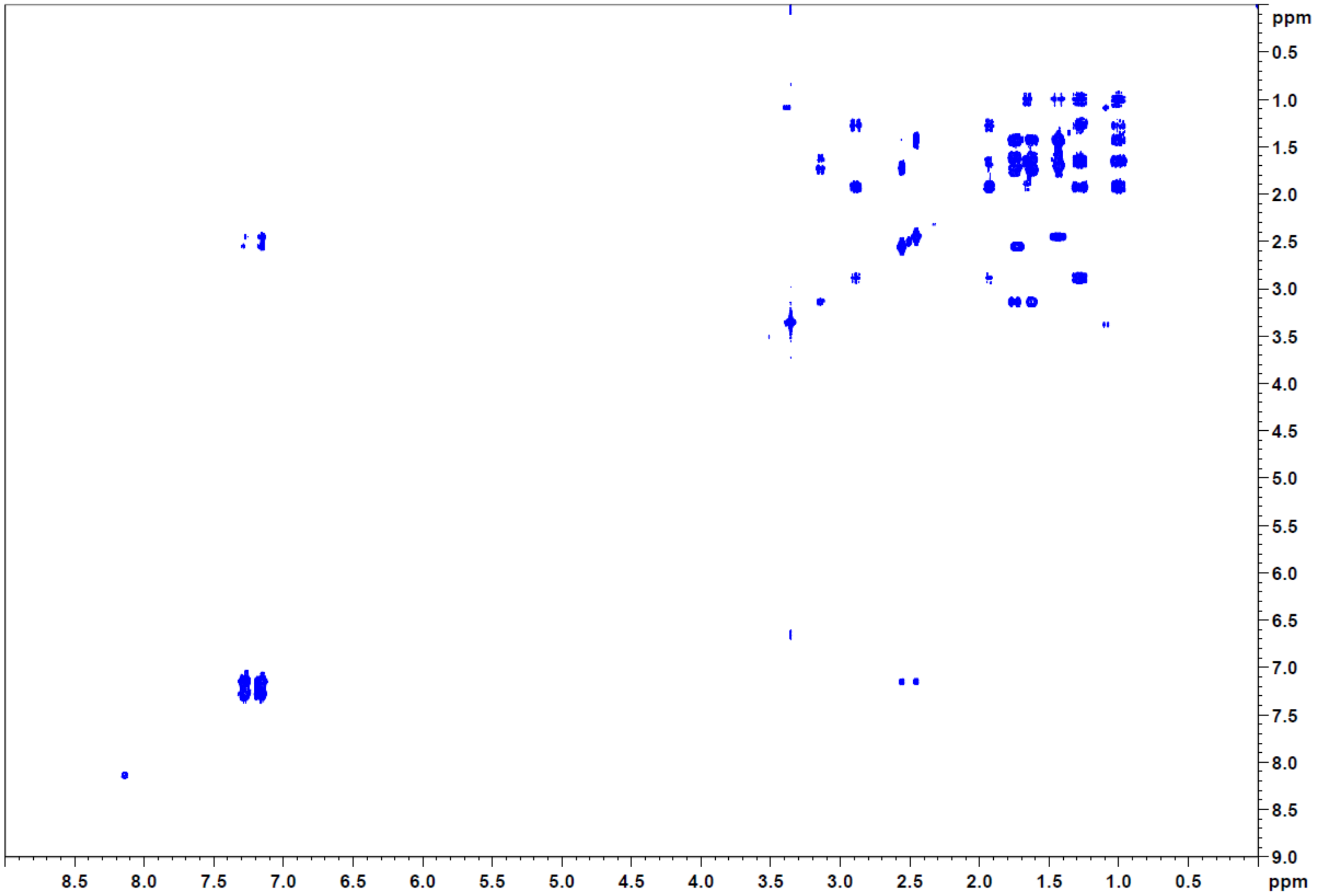

Figure NMR24 COSY spectrum of *cis*/*trans*-4-benzylcyclohexan-1-aminium chloride (*cis*/*trans*-**1c**·HCl)

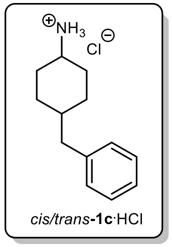

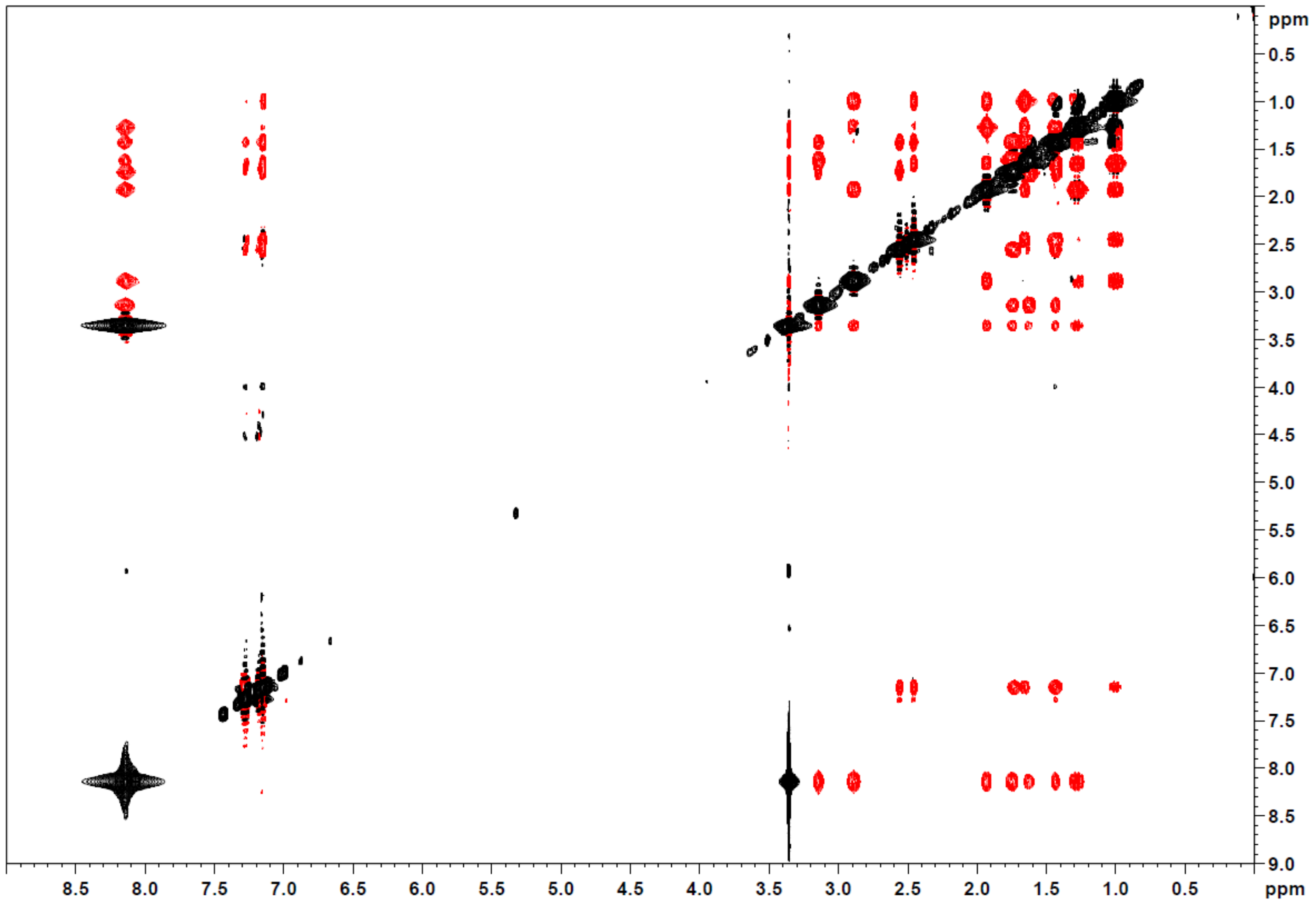

Figure NMR25 NOESY spectrum of *cis*/*trans*-4-benzylcyclohexan-1-aminium chloride (*cis*/*trans*-**1c**·HCl)

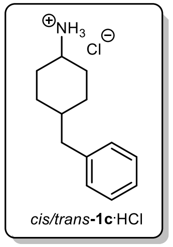

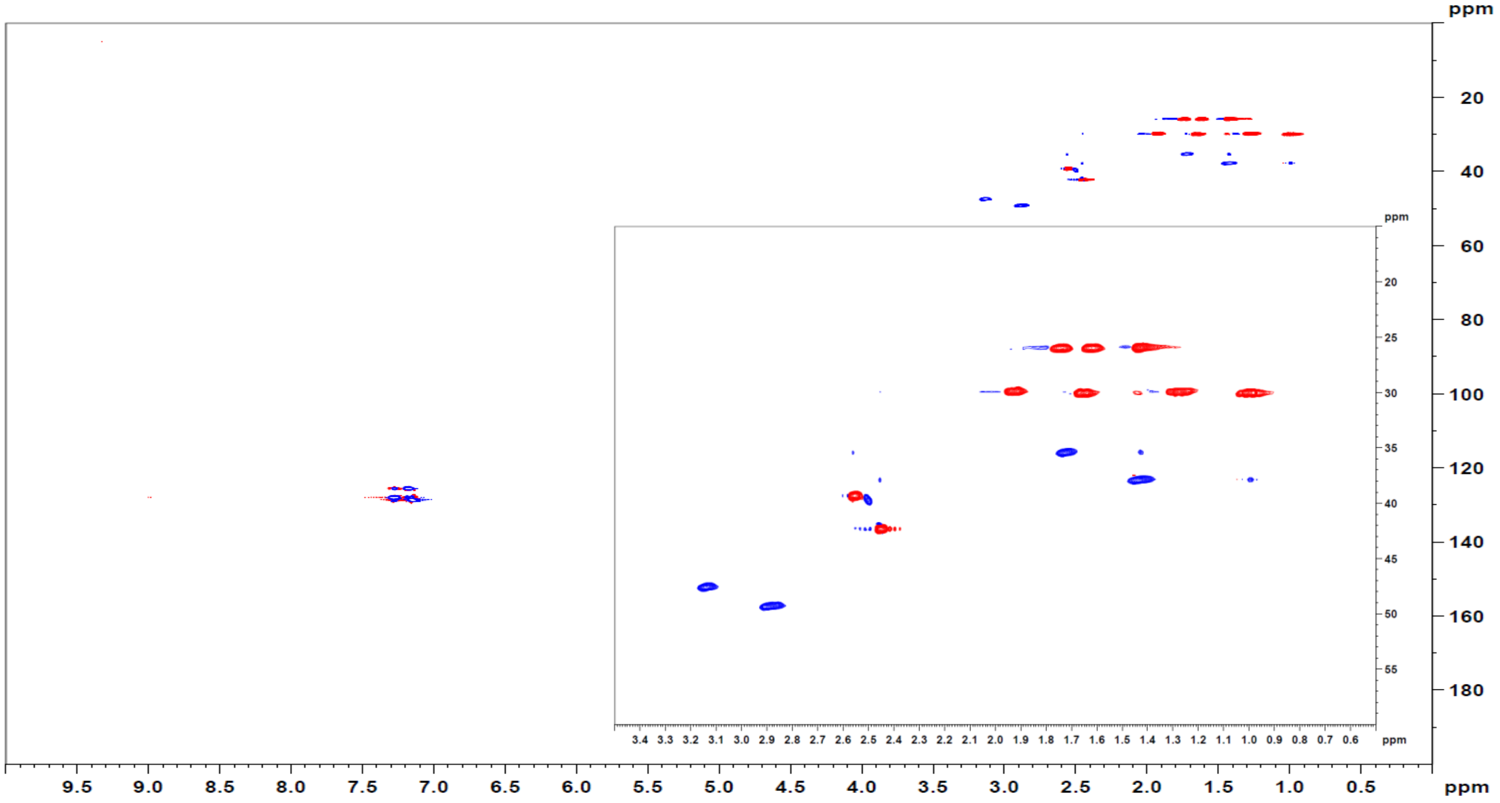

Figure NMR26 HSQC spectrum of *cis*/*trans*-4-benzylcyclohexan-1-aminium chloride (*cis*/*trans*-**1c**·HCl)

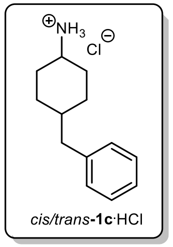

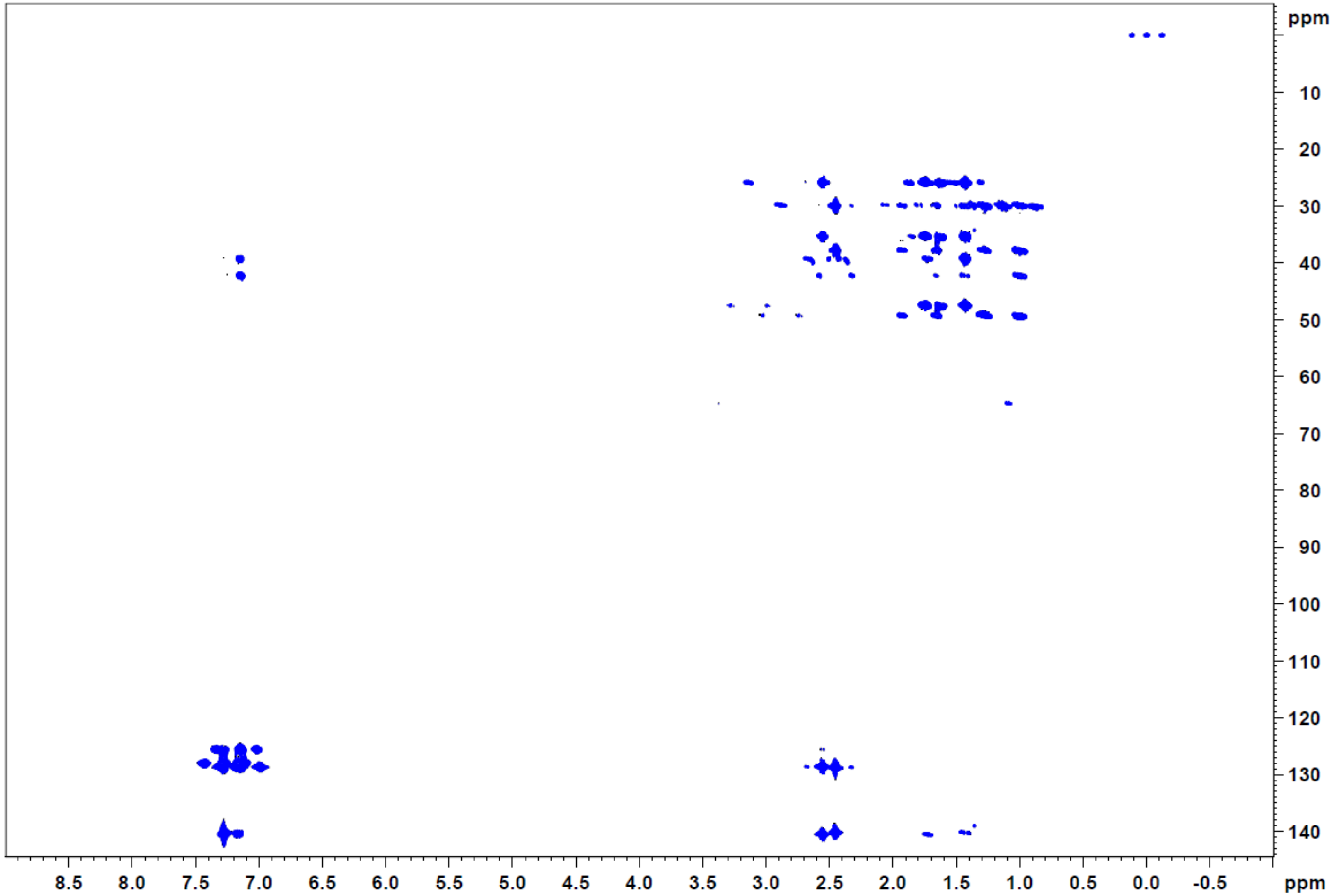

Figure NMR27 HMBC spectrum of *cis*/*trans*-4-benzylcyclohexan-1-aminium chloride (*cis*/*trans*-**1c**·HCl)

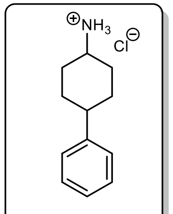

cis/trans-**1d**·HCl

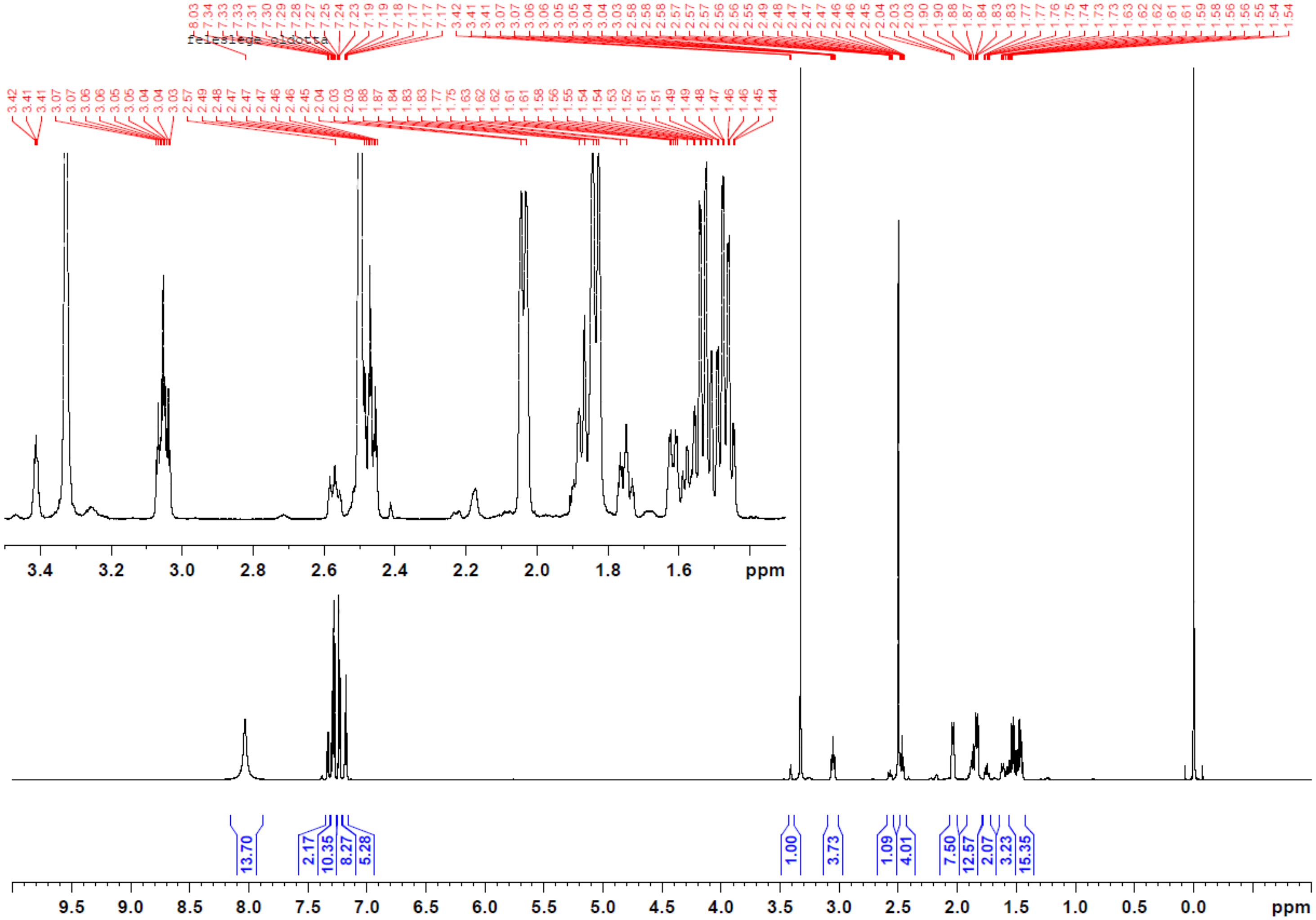

Figure NMR28 ^{1}H-NMR (500 MHz, DMSO-d_6) spectrum of *cis*/*trans*-4-phenylcyclohexan-1-aminium chloride (*cis*/*trans*-**1d·**HCl)

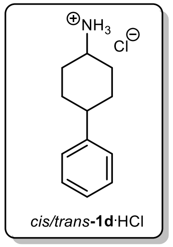

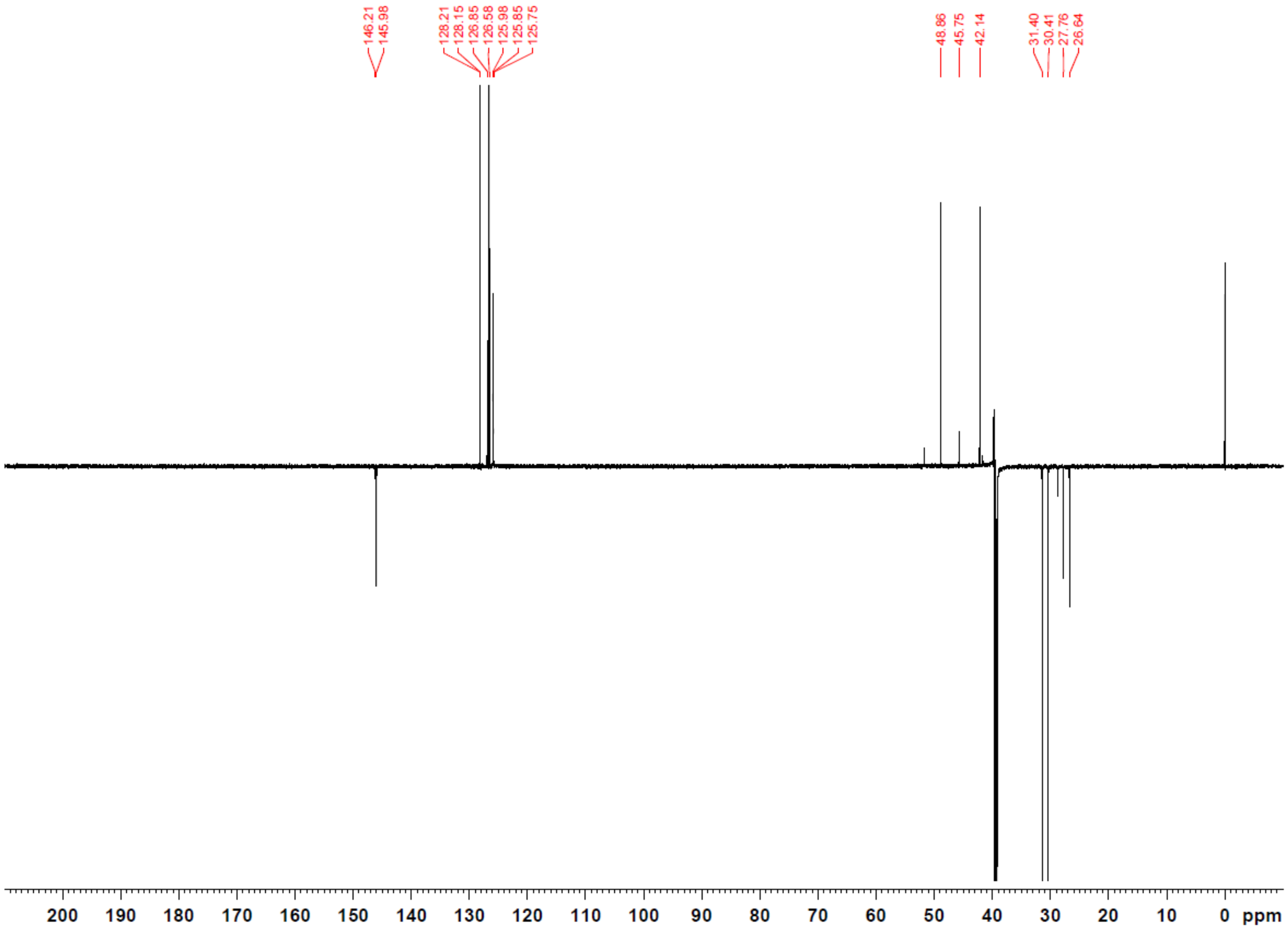

Figure NMR29 DEPTQ -NMR (126 MHz, DMSO-d_6) spectrum of *cis*/*trans*-4-phenylcyclohexan-1-aminium chloride (*cis*/*trans*-**1f**·HCl)

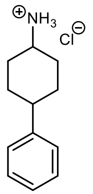

cis/trans-**1d**·HCl

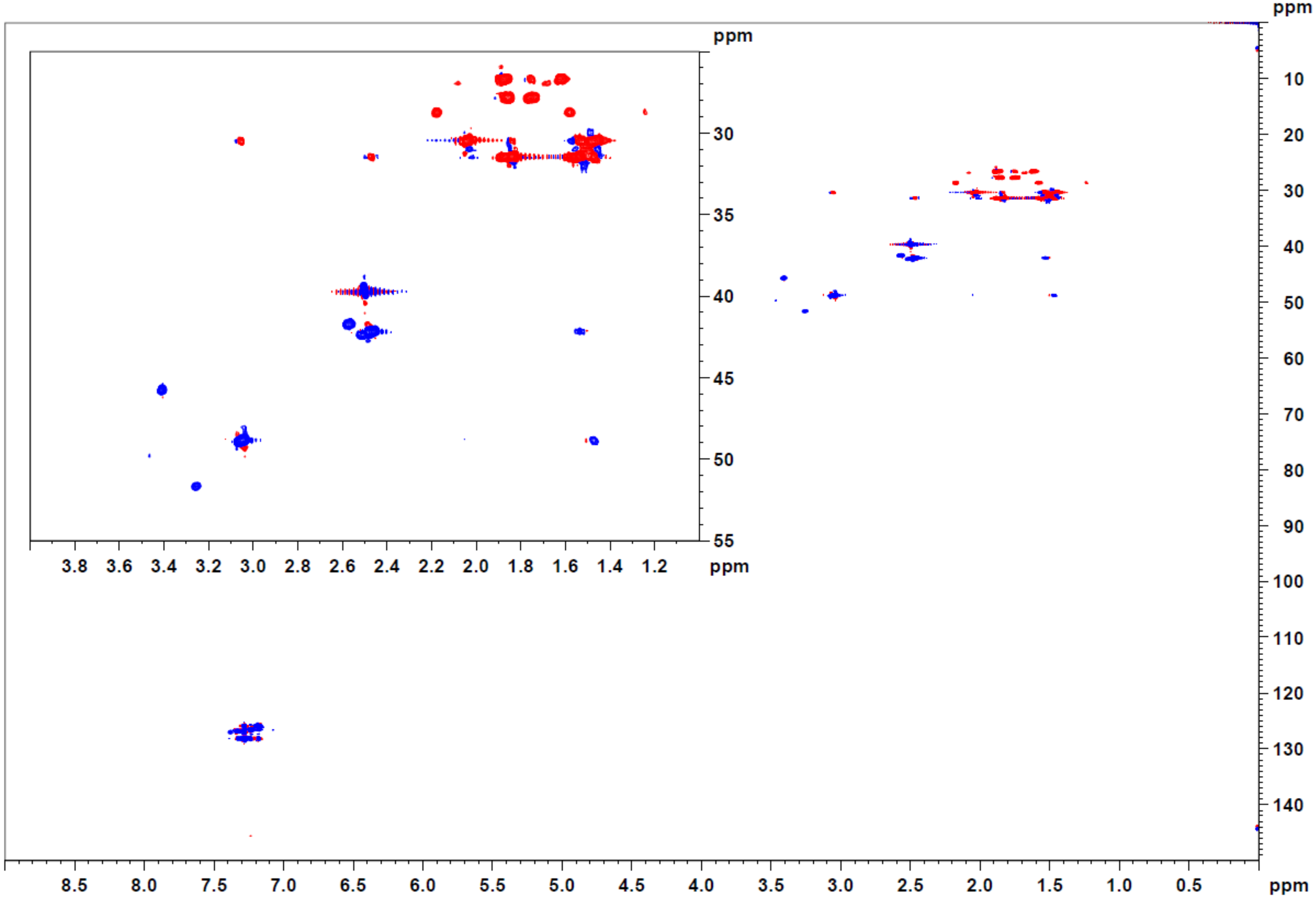

Figure NMR30 HSQC spectrum of *cis*/*trans*-4-phenylcyclohexan-1-aminium chloride (*cis*/*trans*-**1d**·HCl)

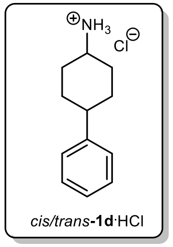

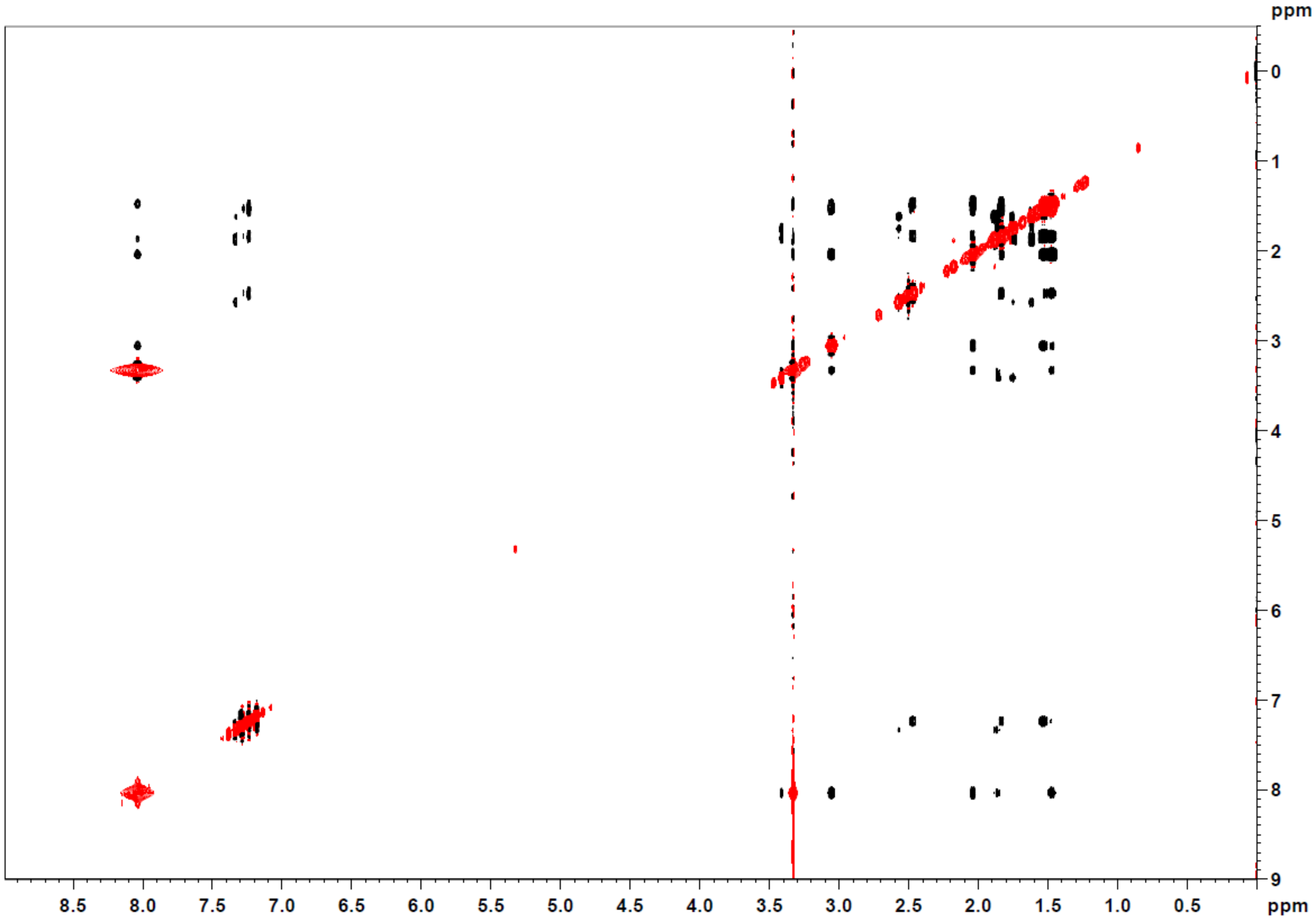

Figure NMR31 NOESY spectrum of *cis*/*trans*-4-phenylcyclohexan-1-aminium chloride (*cis*/*trans*-**1d**·HCl)

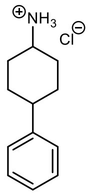

cis/trans-**1d**·HCl

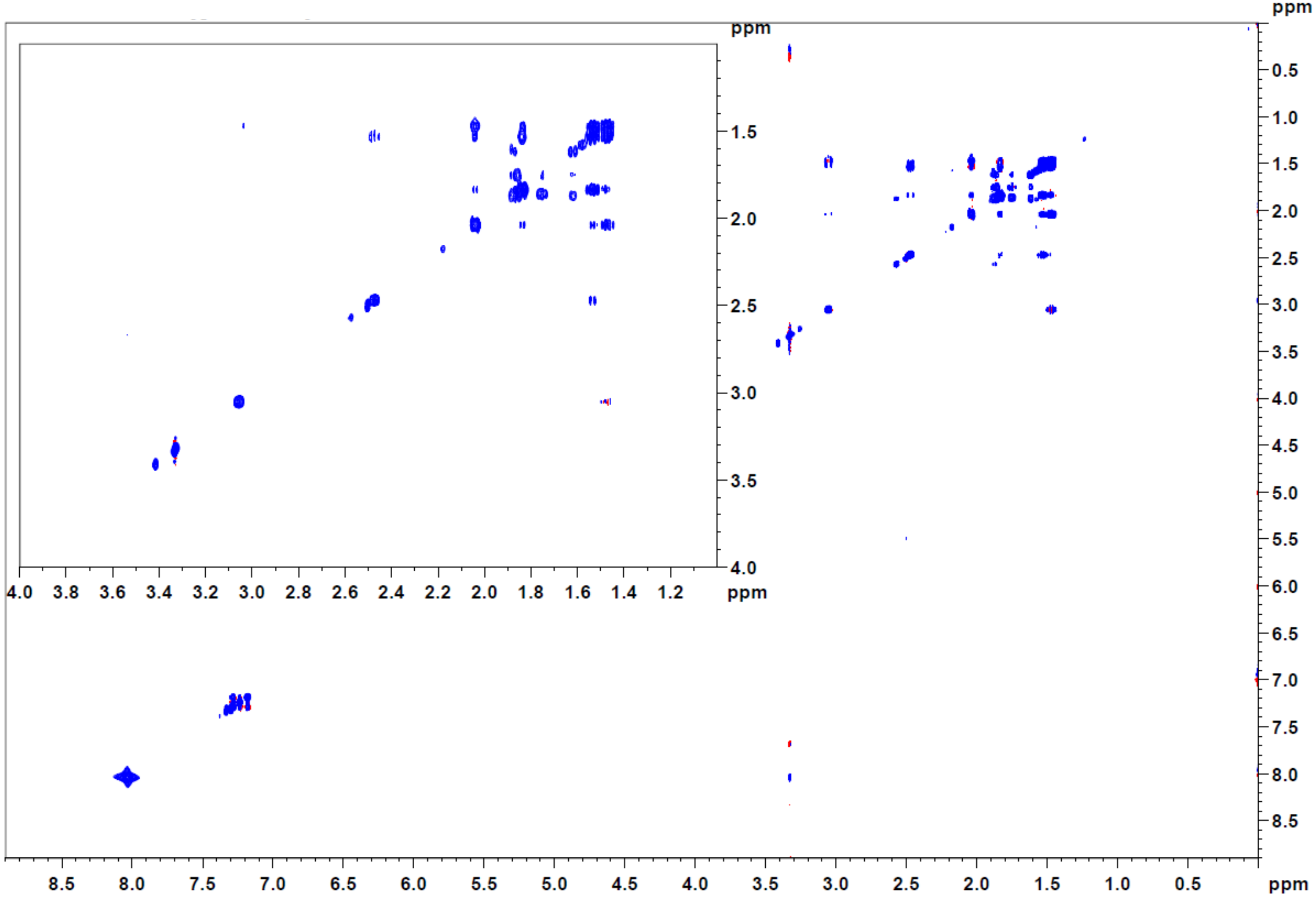

Figure NMR32 MLEV spectrum of *cis*/*trans*-4-phenylcyclohexan-1-aminium chloride (*cis*/*trans*-**1d**·HCl)

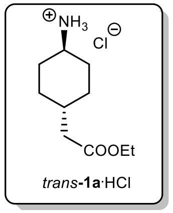

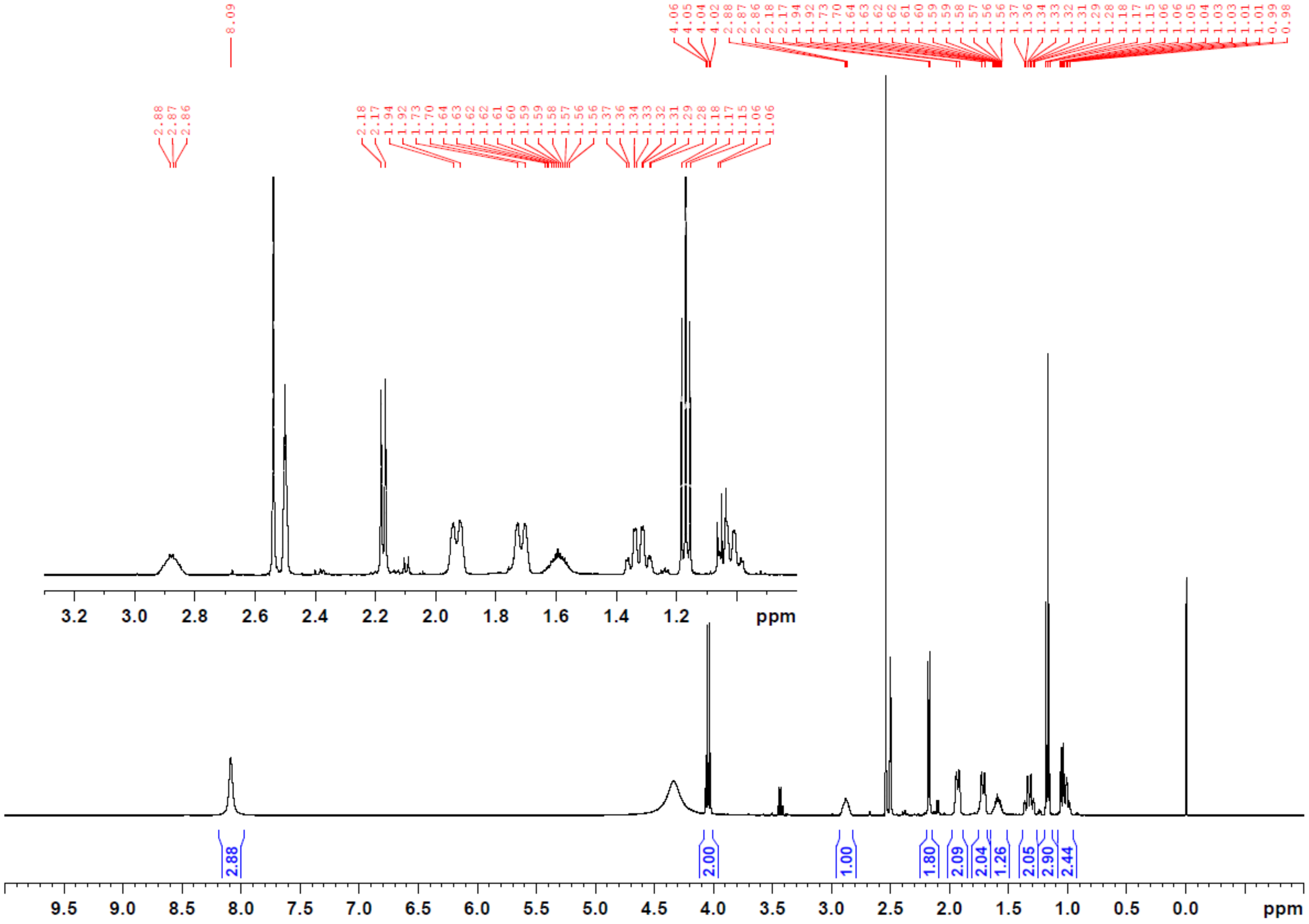

Figure NMR33 ^{1}H-NMR (500 MHz, DMSO-d_6) spectrum of *trans*-4-(2-ethoxy-2-oxoethyl)cyclohexan-1-aminium chloride (*trans*-**1a**·HCl)

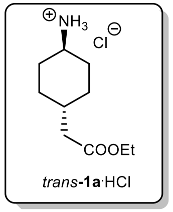

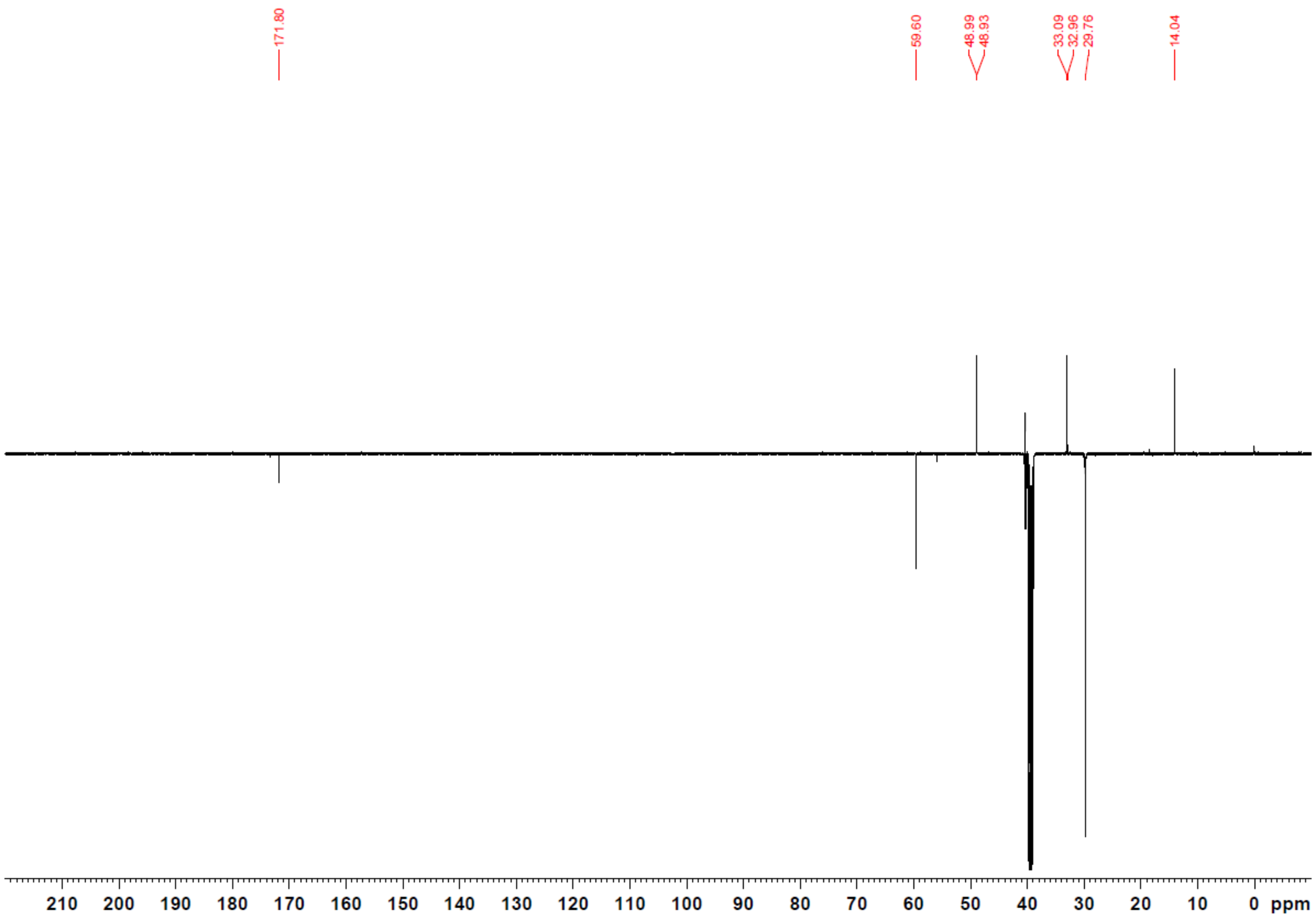

Figure NMR34 DEPTQ – NMR (125 MHz, DMSO-d_6) spectrum of *trans*-4-(2-ethoxy-2-oxoethyl)cyclohexan-1-aminium chloride (*trans*-**1a**·HCl)

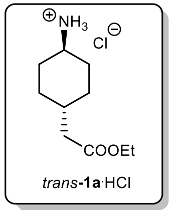

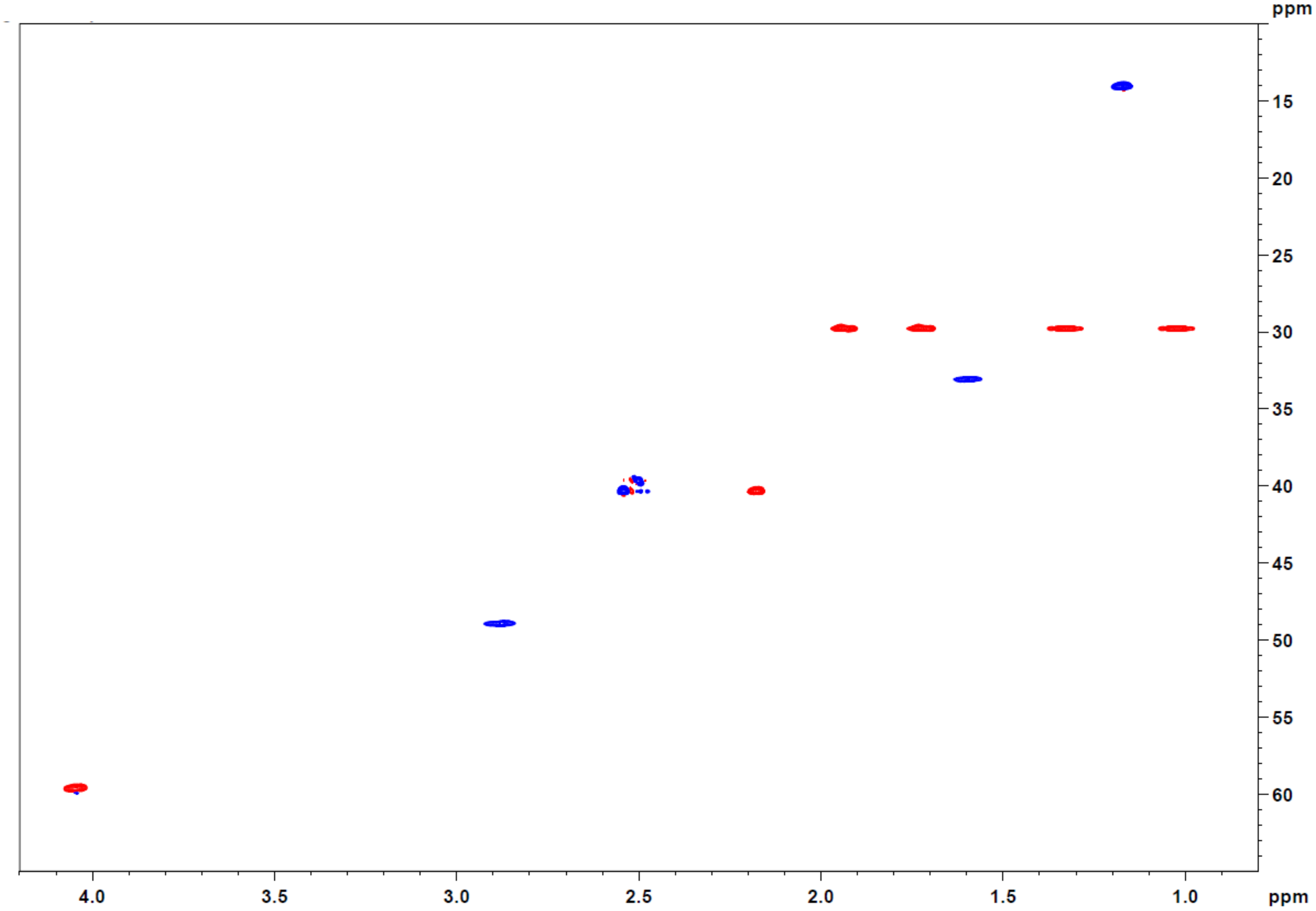

Figure NMR35 HSQC spectrum of *trans*-4-(2-ethoxy-2-oxoethyl)cyclohexan-1-aminium chloride (*trans*-**1a**·HCl)

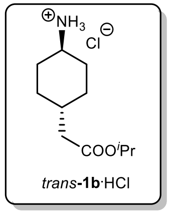

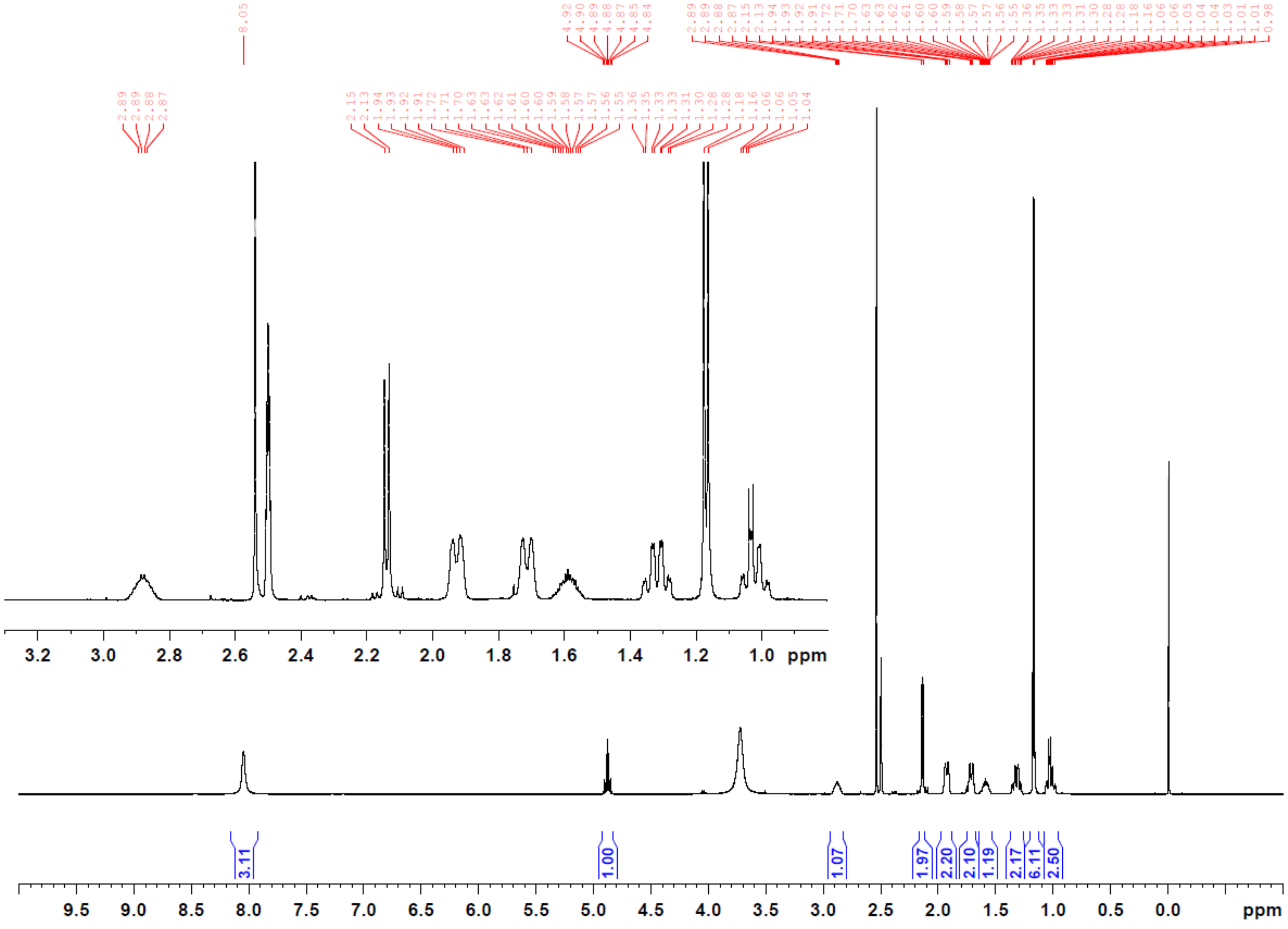

Figure NMR36 ^{1}H-NMR (500 MHz, DMSO-d_6) spectrum of *trans*-4-(2-isopropoxy-2-oxoethyl)cyclohexan-1-aminium chloride (*trans*-**1b**·HCl)

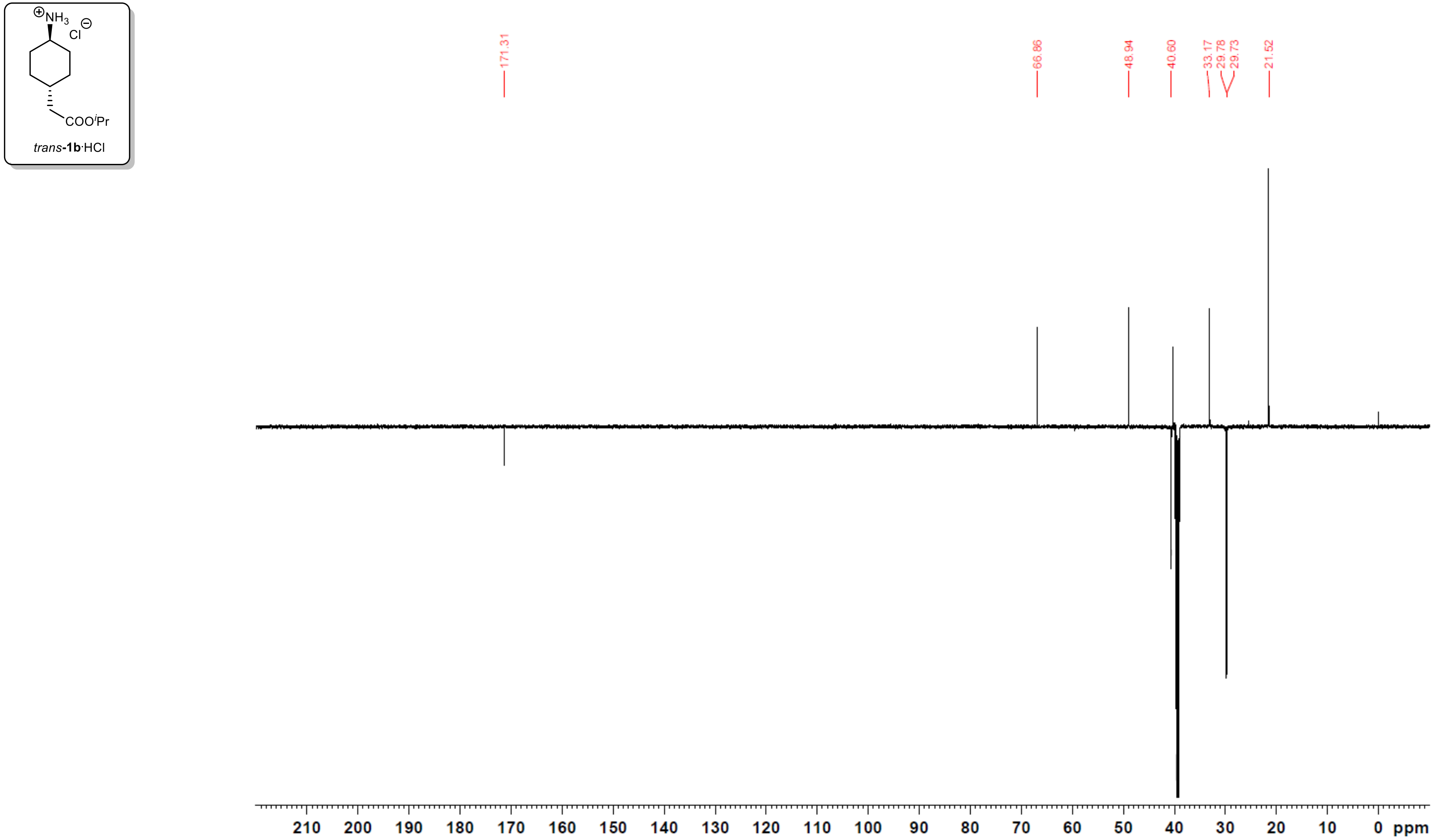

Figure NMR37 DEPTQ -NMR (125 MHz, DMSO-*d*$_6$) spectrum of *trans*-4-(2-isopropoxy-2-oxoethyl)cyclohexan-1-aminium chloride (*trans*-**1b**·HCl)

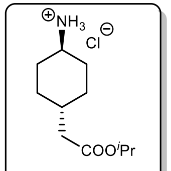

trans-**1b**·HCl

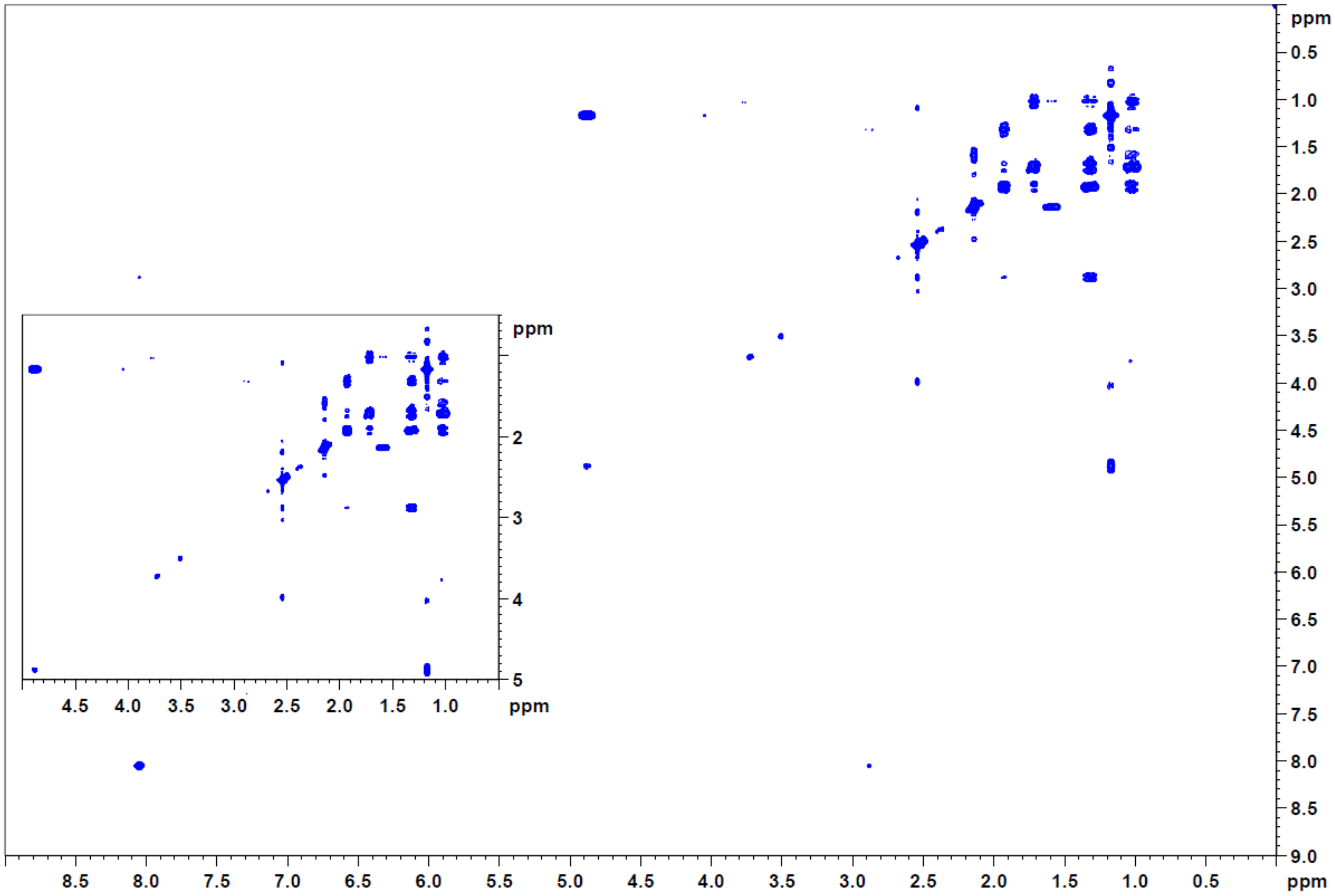

Figure NMR38 COSY spectrum of *trans*-4-(2-isopropoxy-2-oxoethyl)cyclohexan-1-aminium chloride (*trans*-**1b**·HCl)

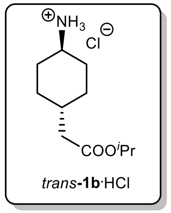

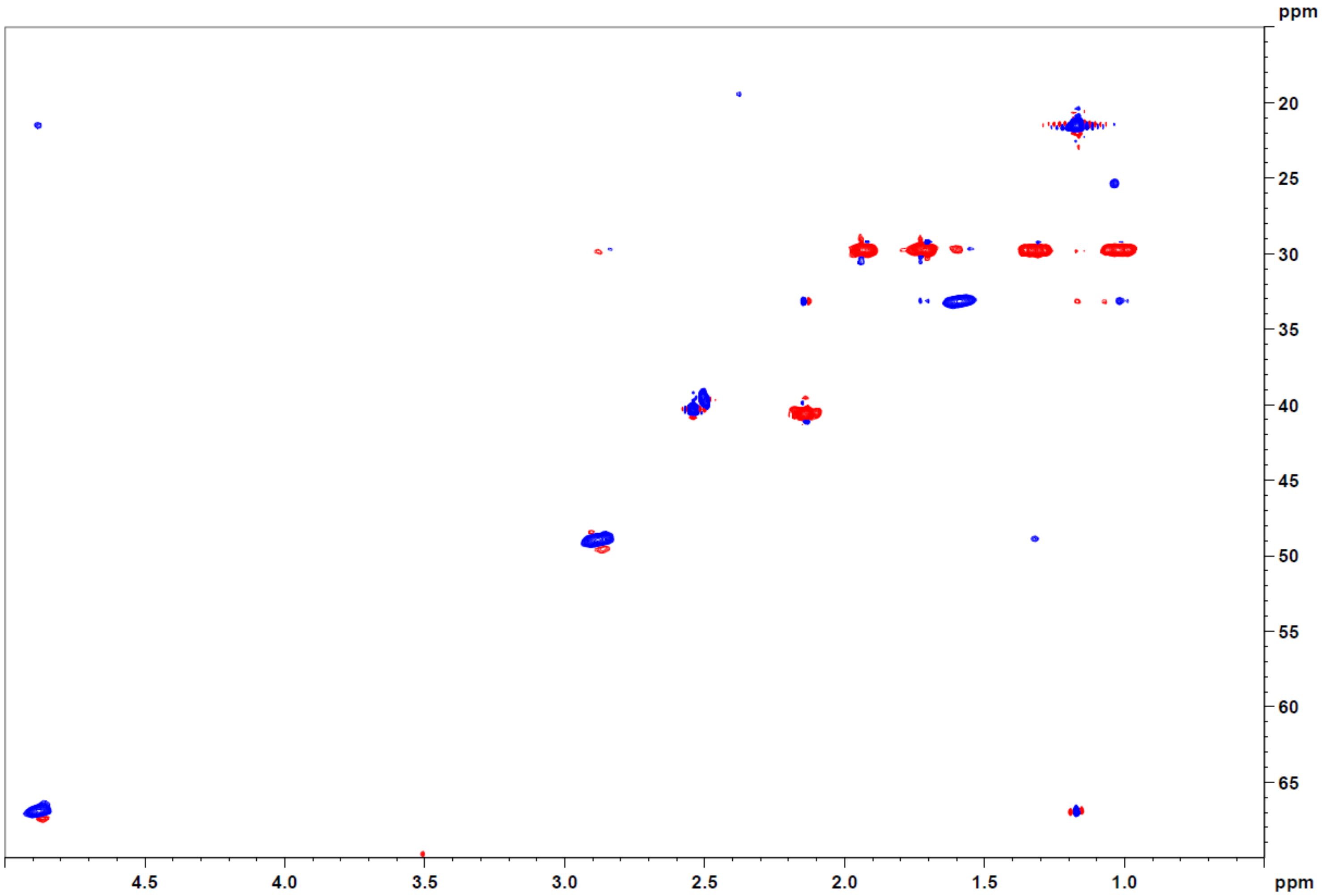

Figure NMR39 HSQC spectrum of *trans*-4-(2-isopropoxy-2-oxoethyl)cyclohexan-1-aminium chloride (*trans*-**1b**·HCl)

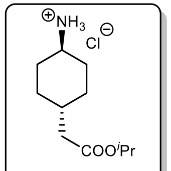

trans-**1b**·HCl

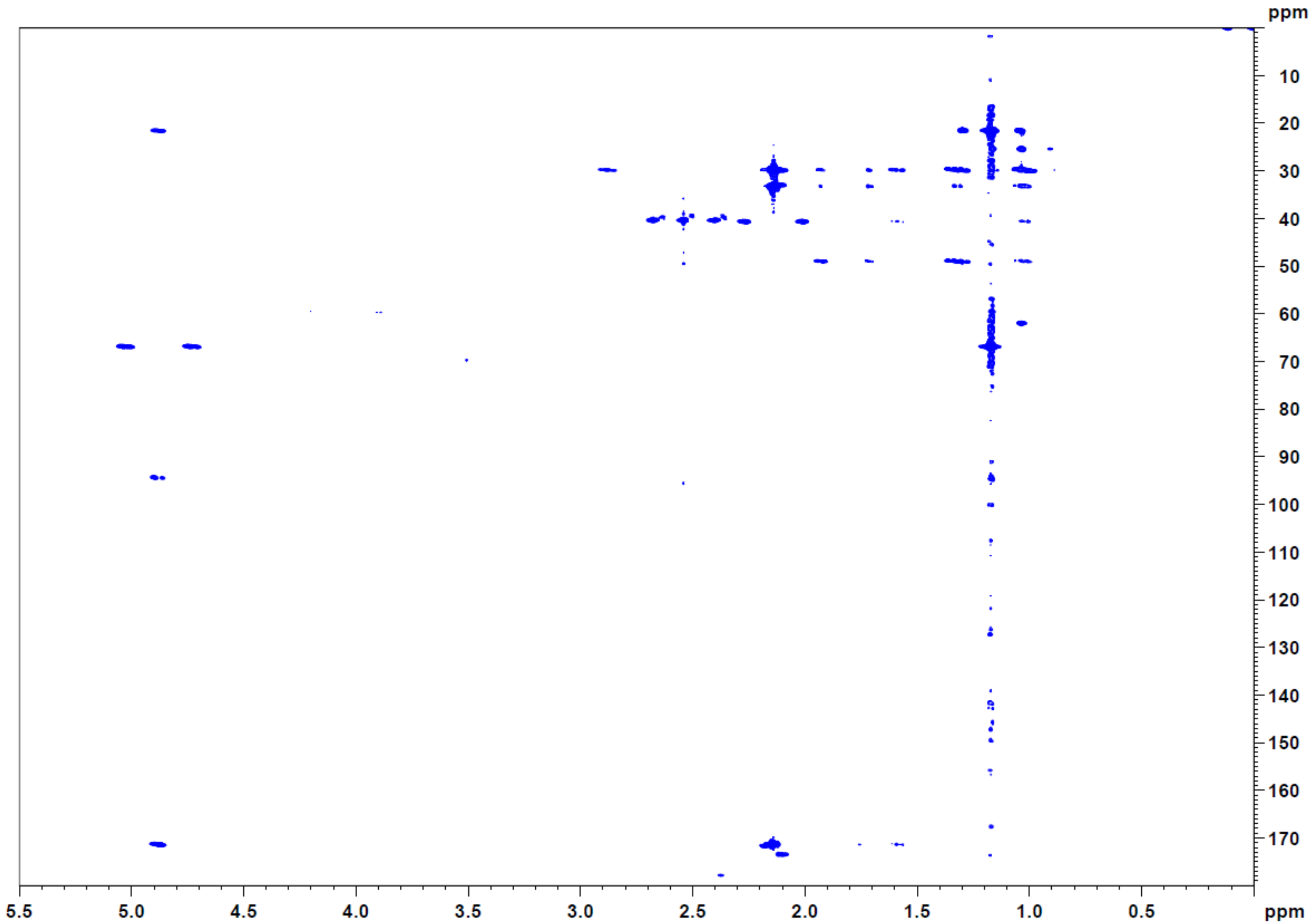

Figure NMR40 HMBC spectrum of *trans*-4-(2-isopropoxy-2-oxoethyl)cyclohexan-1-aminium chloride (*trans*-**1b**·HCl)

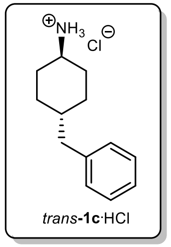

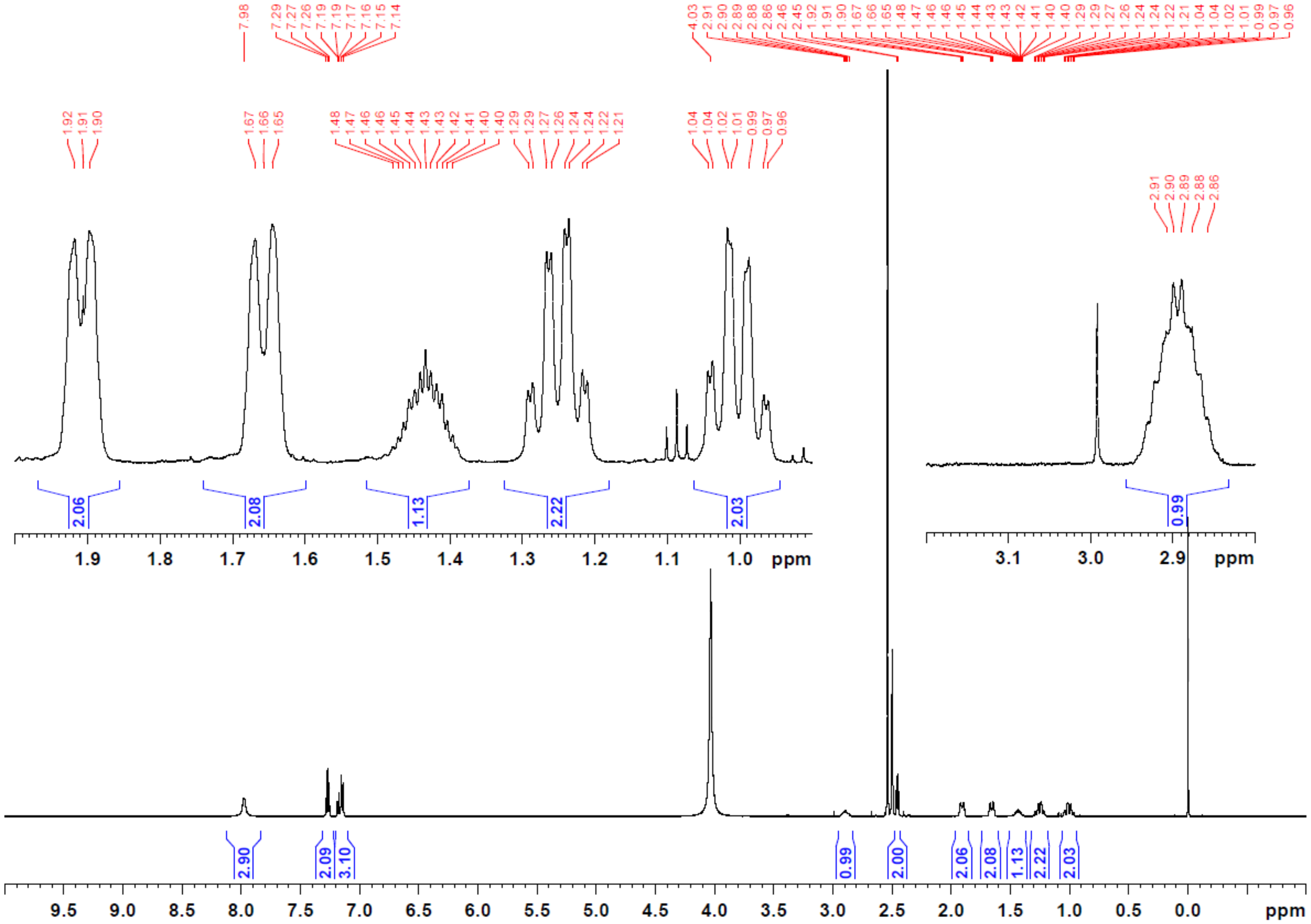

Figure NMR41 ^{1}H-NMR (500 MHz, DMSO-d_6) spectrum of *trans*-4-benzylcyclohexan-1-aminium chloride (*trans*-**1c**·HCl)

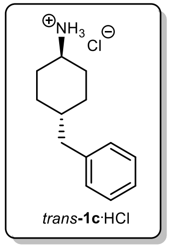

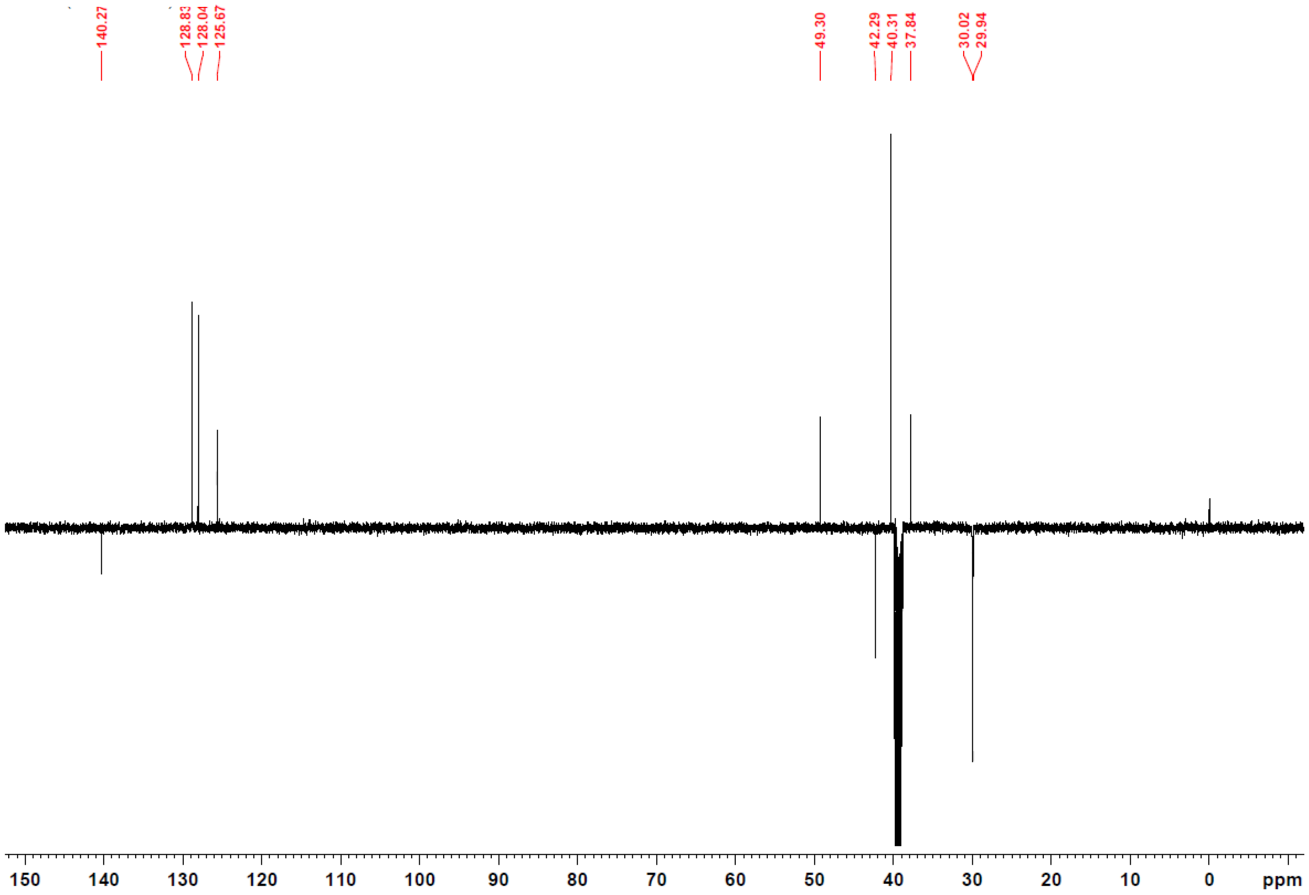

Figure NMR42 DEPTQ -NMR (125 MHz, DMSO-d_6) spectrum of *trans*-4-benzylcyclohexan-1-aminium chloride (*trans*-**1c**·HCl)

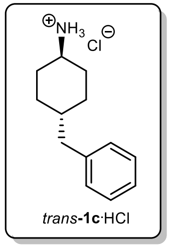

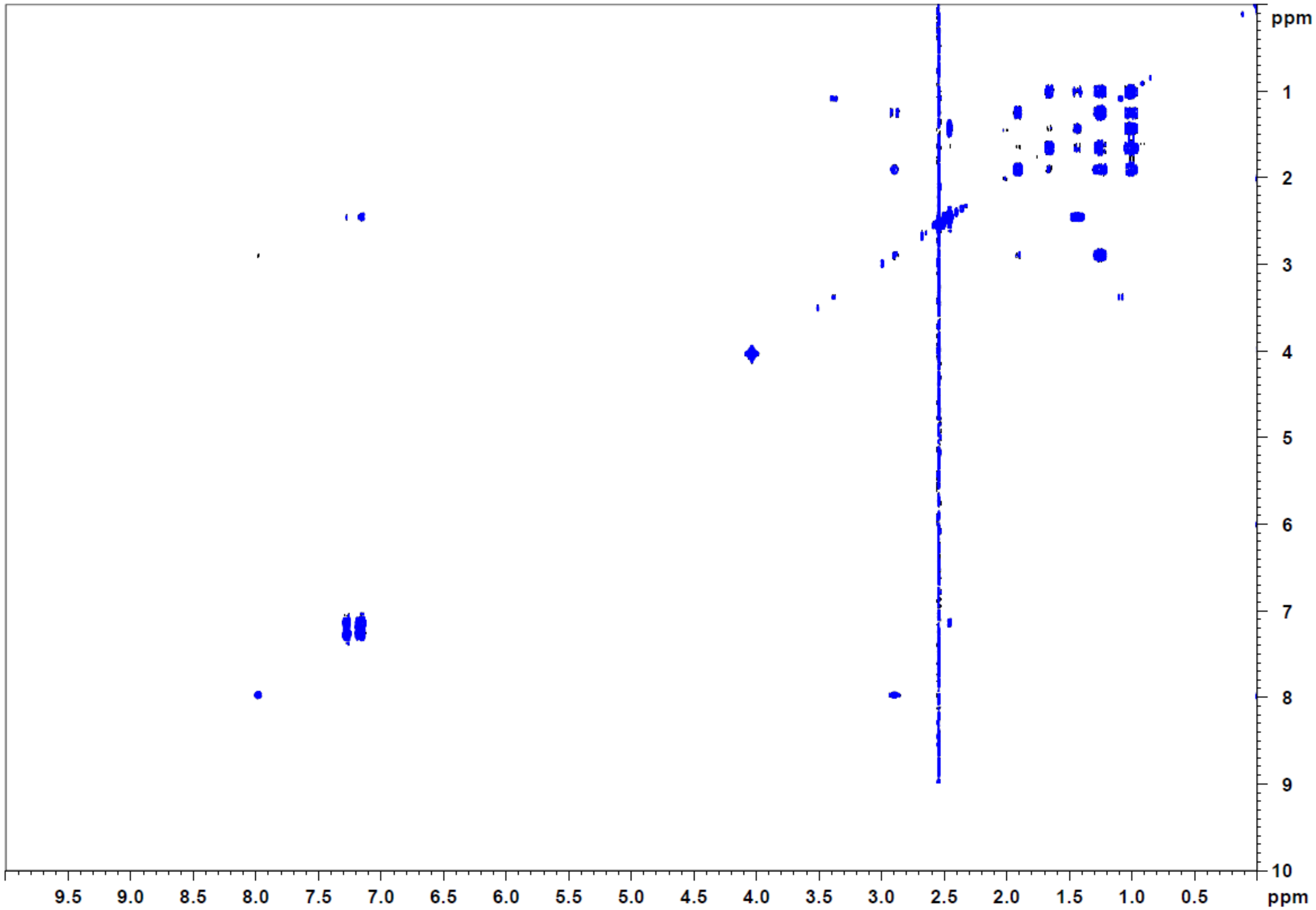

Figure NMR43 COSY spectrum of *trans*-4-benzylcyclohexan-1-aminium chloride (*trans*-**1c**·HCl)

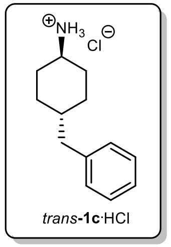

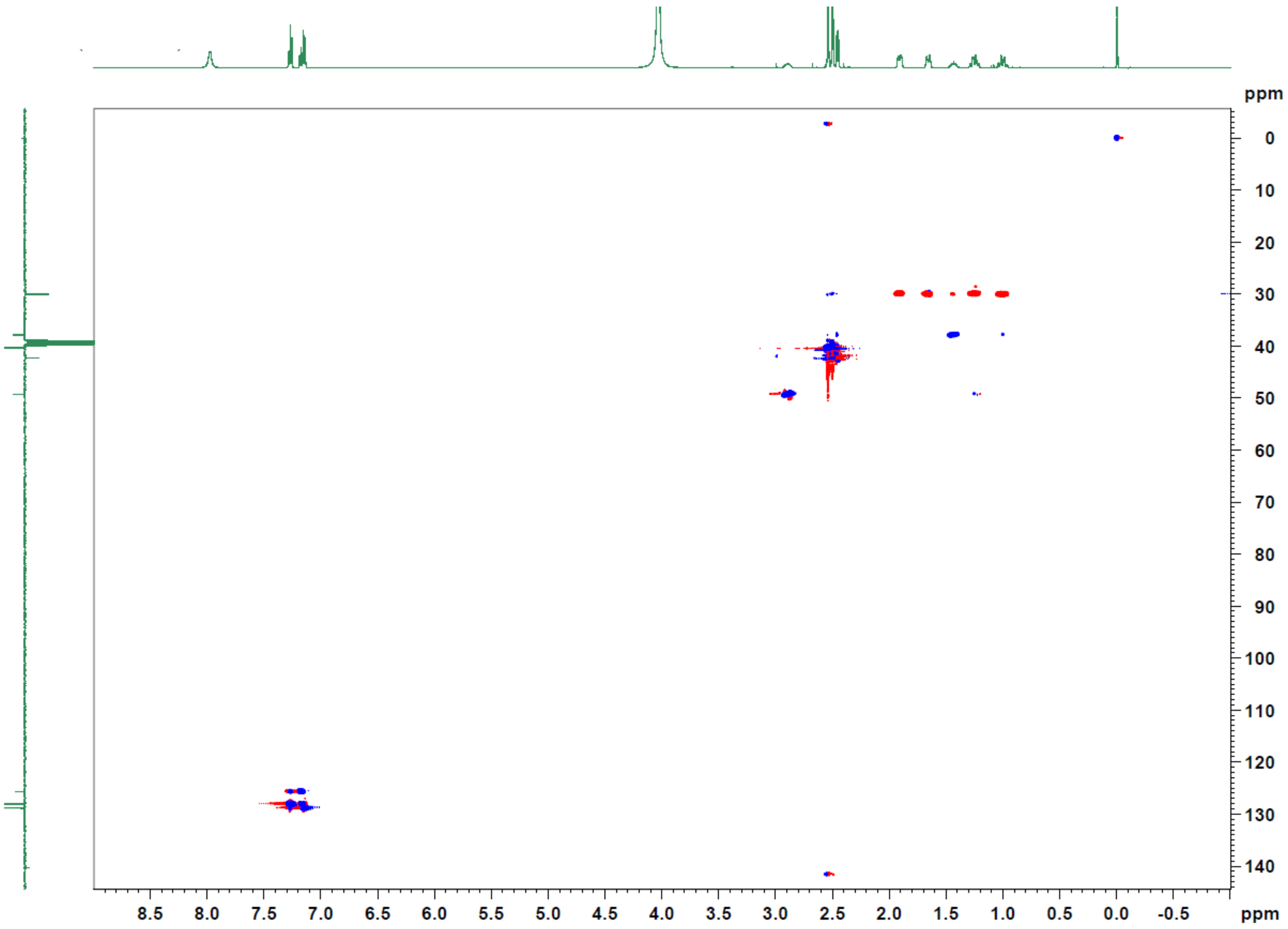

Figure NMR44 HSQC spectrum of *trans*-4-benzylcyclohexan-1-aminium chloride (*trans*-**1c**·HCl)

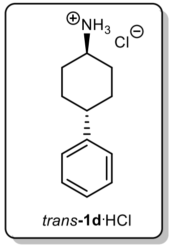

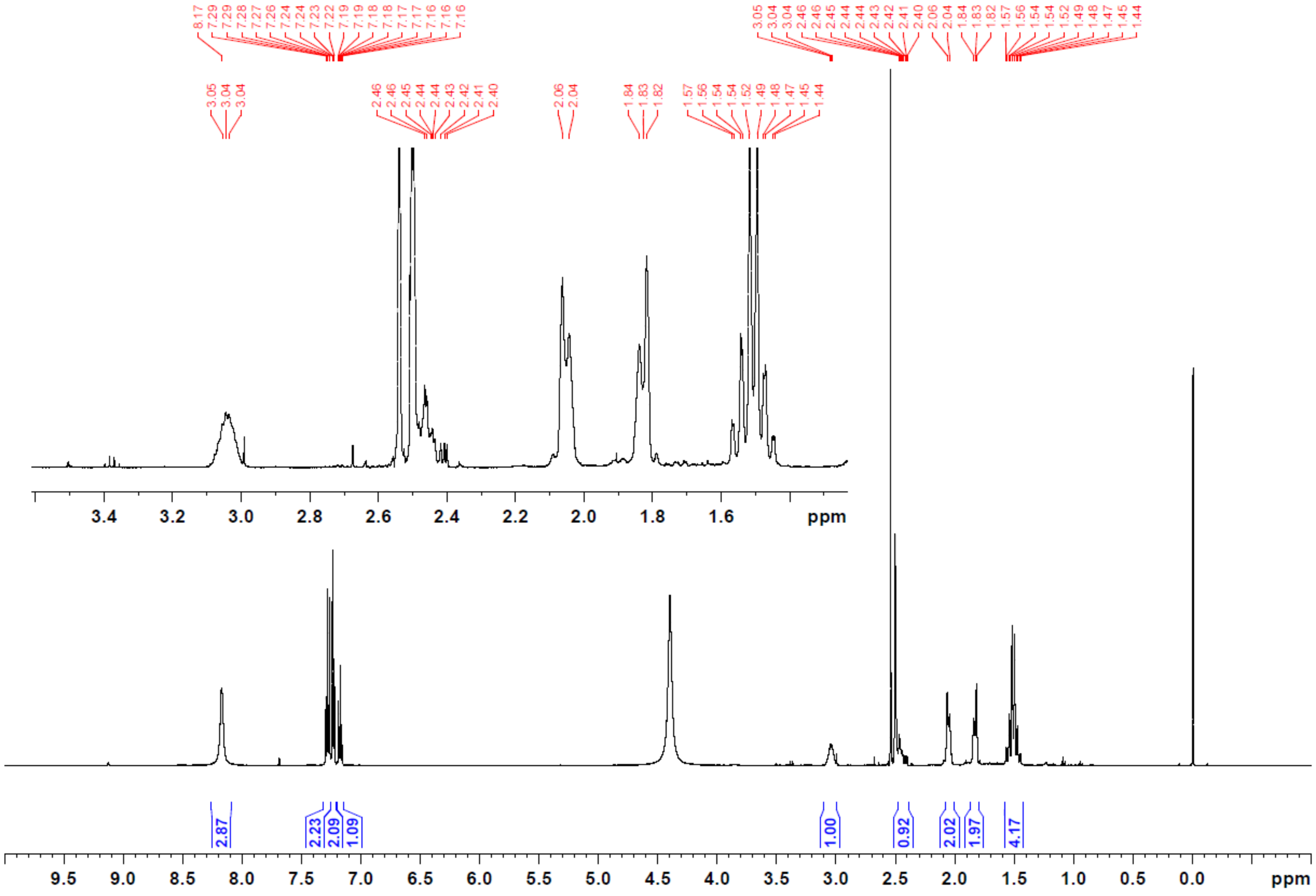

Figure NMR45 ^{1}H-NMR spectrum of *trans*-4-phenylcyclohexan-1-aminium chloride (*trans*-**1d**·HCl)

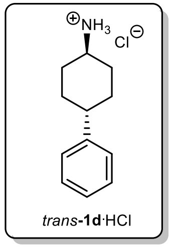

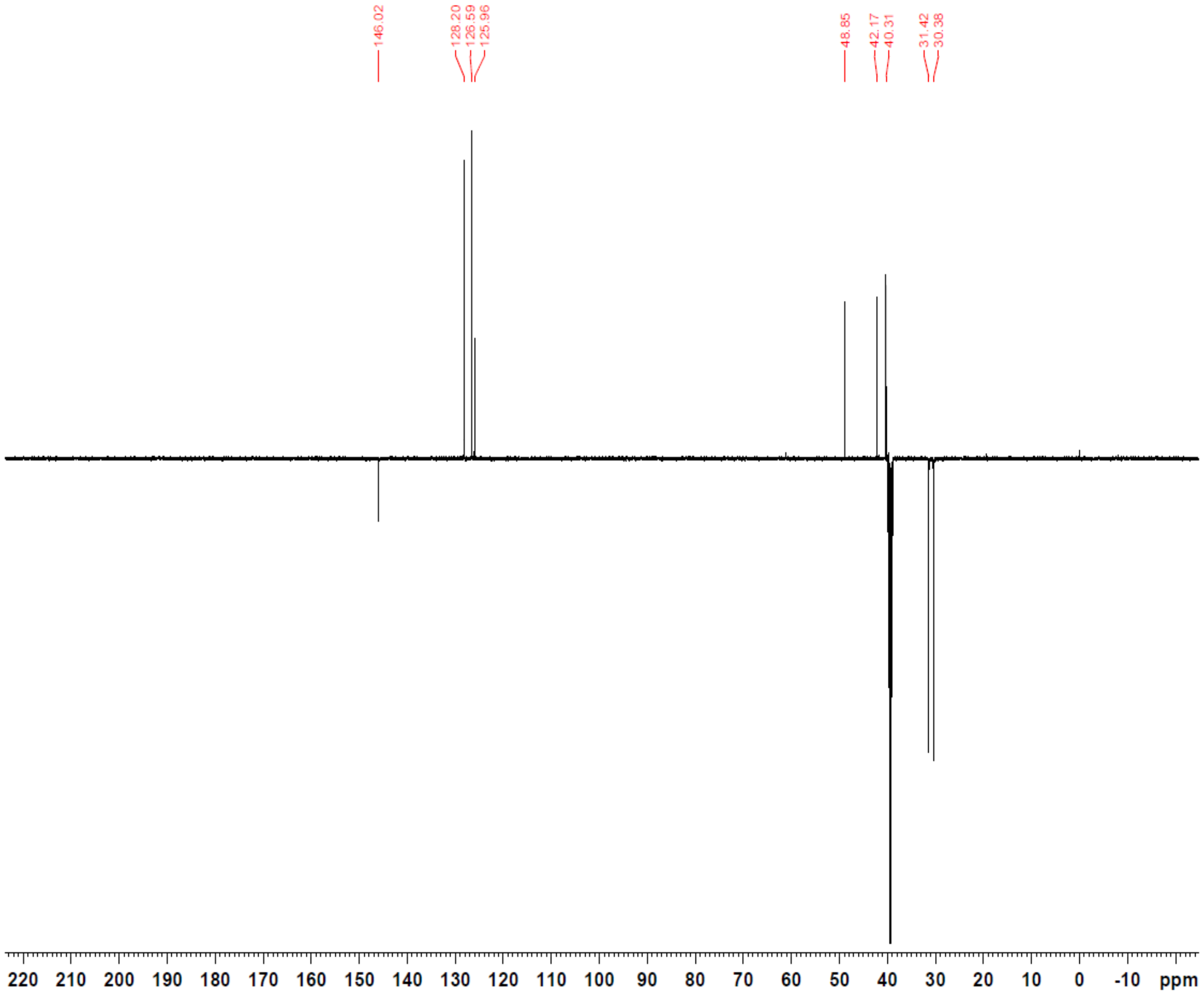

Figure NMR46 DEPTQ -NMR spectrum of *trans*-4-phenylcyclohexan-1-aminium chloride (*trans*-**1d**·HCl)

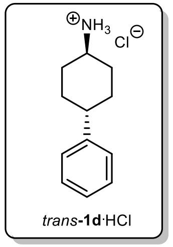

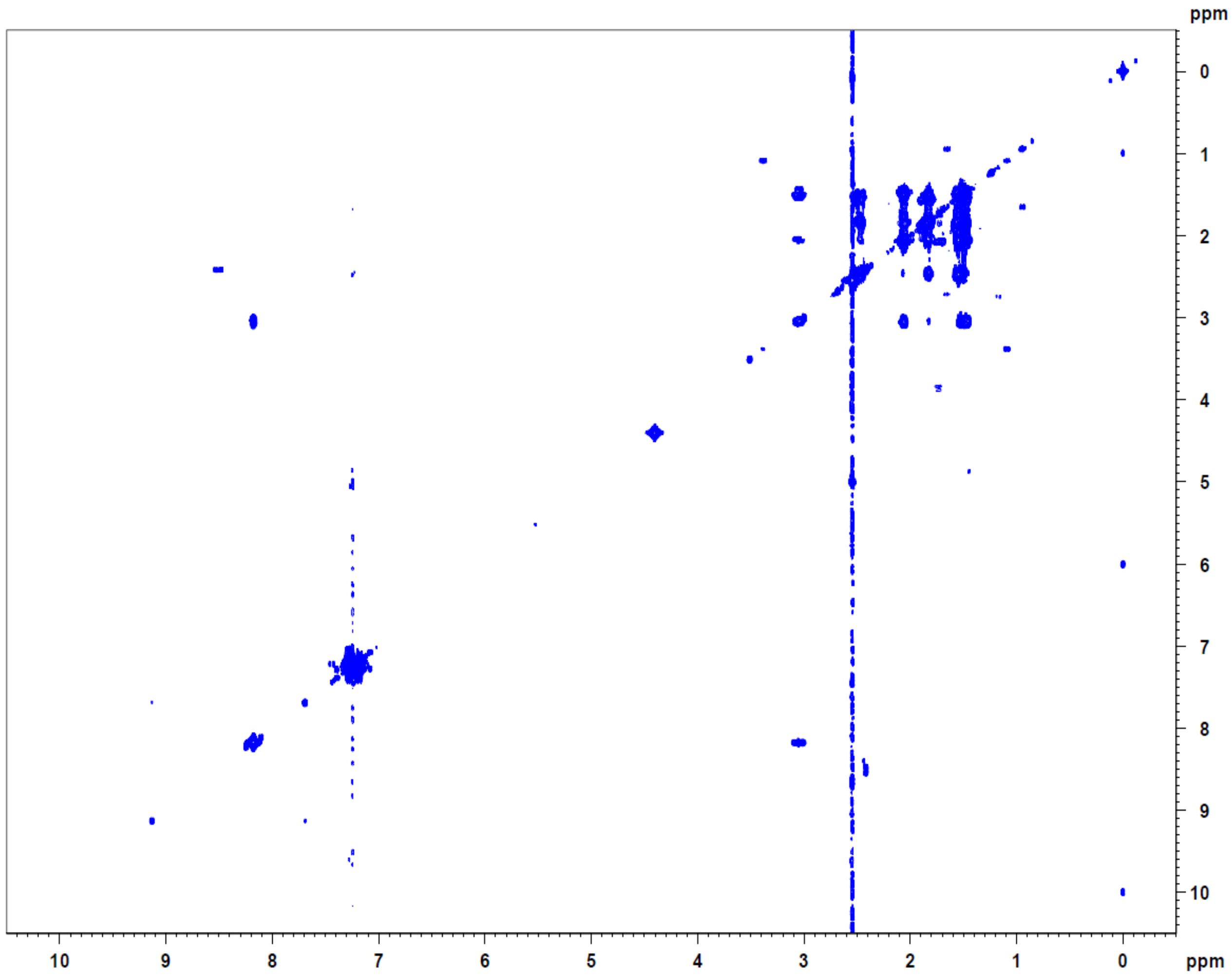

Figure NMR47 COSY spectrum of *trans*-4-phenylcyclohexan-1-aminium chloride (*trans*-**1d**·HCl)

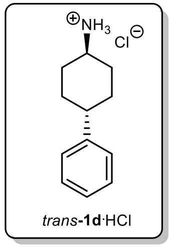

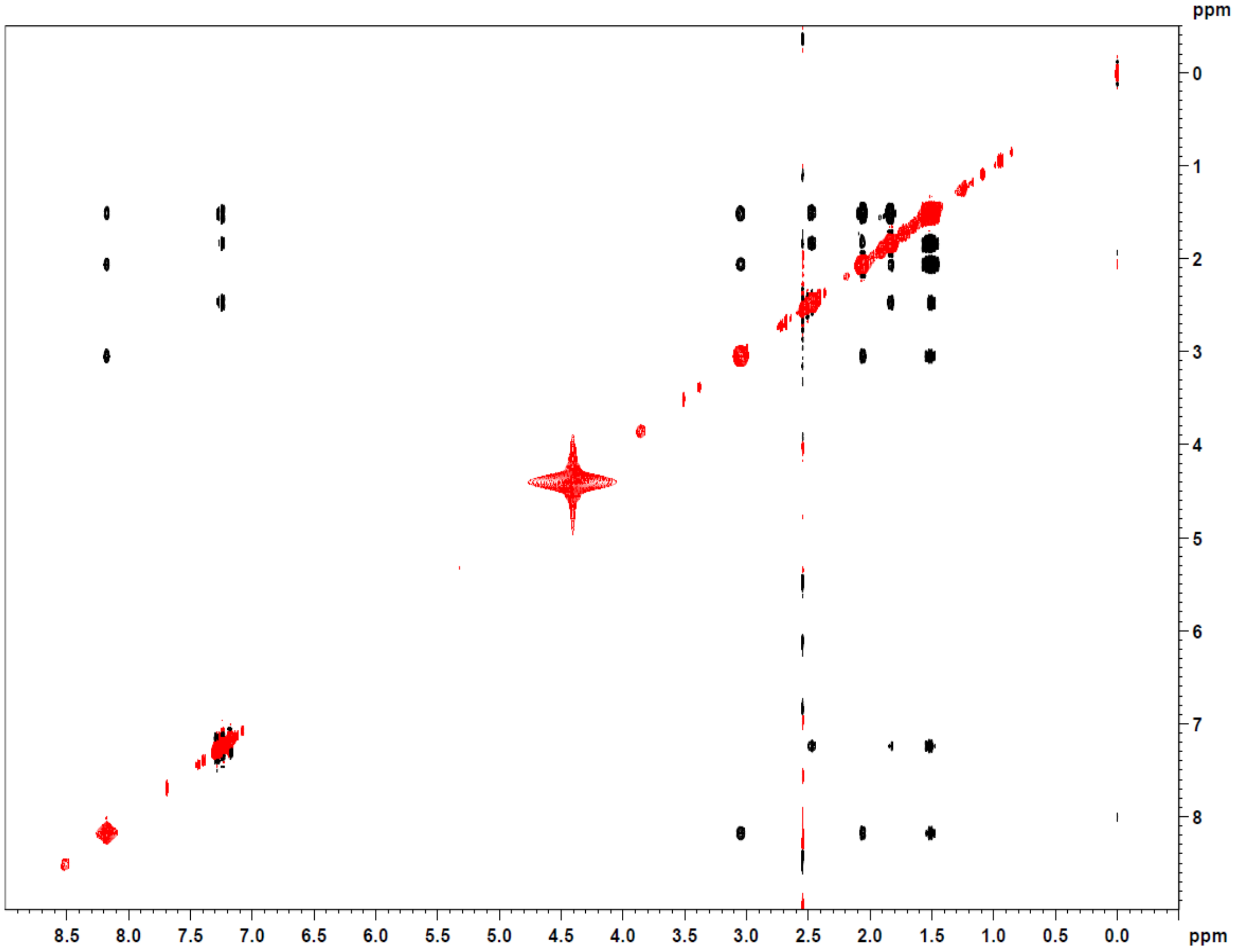

Figure NMR48 NOESY spectrum of *trans*-4-phenylcyclohexan-1-aminium chloride (*trans*-**1d**·HCl)

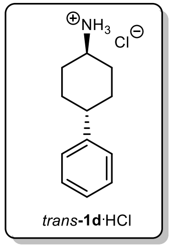

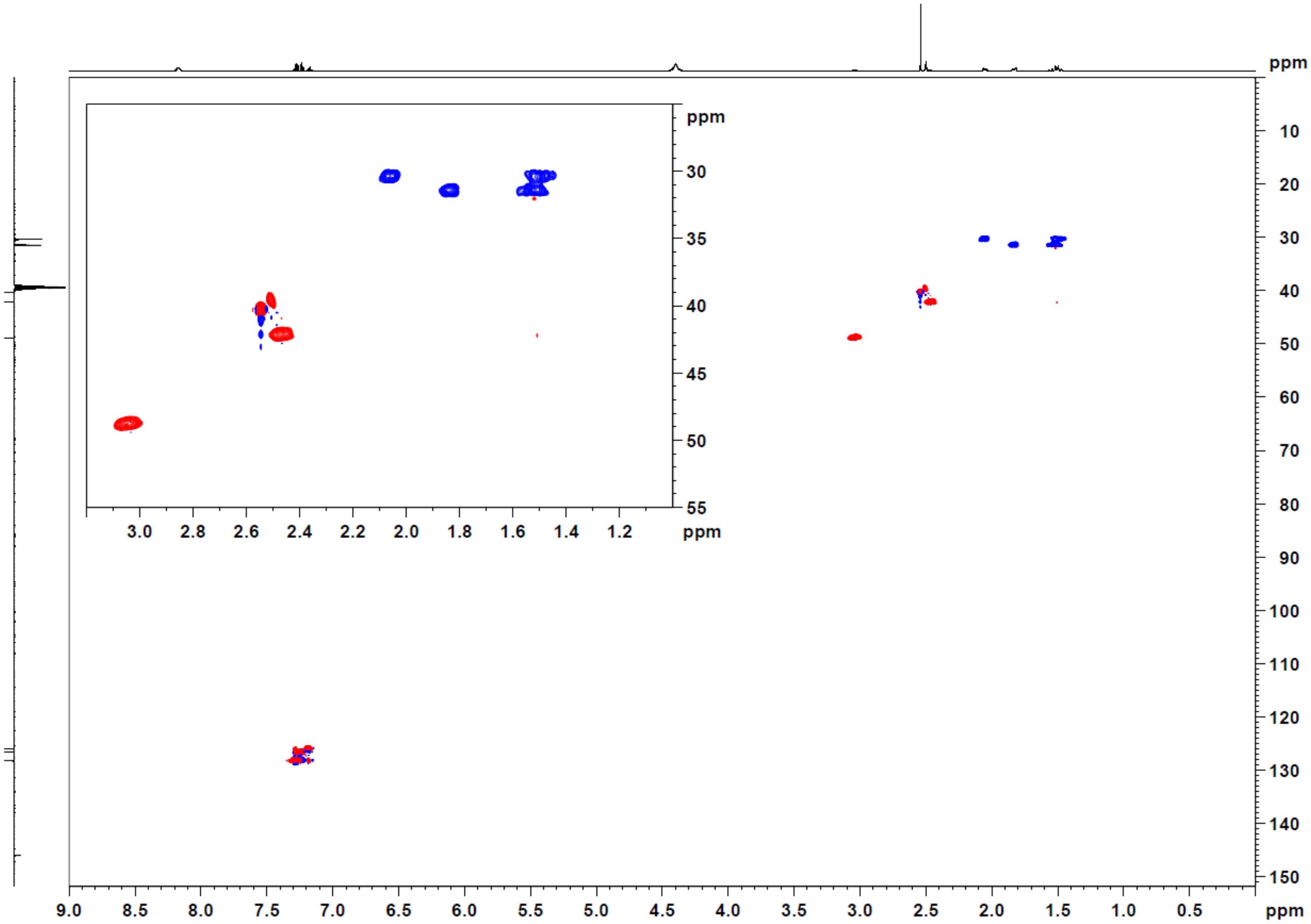

Figure NMR49 HSQC spectrum of *trans*-4-phenylcyclohexan-1-aminium chloride (*trans*-**1d**·HCl)
