# Supplementary material for: Transaminase-catalysis to produce trans-4-substituted cyclohexane-1-amines including a key intermediate towards cariprazine
Source: Commun Chem. 2024 Apr 18;7:86. doi: 10.1038/s42004-024-01148-9 (PMC11026398; doi:10.1038/s42004-024-01148-9)
Supplement: Supplementary file 5 — Supplementary Data 2 [file 42004_2024_1148_MOESM5_ESM.pdf]

The GC chromatograms for reactions and products

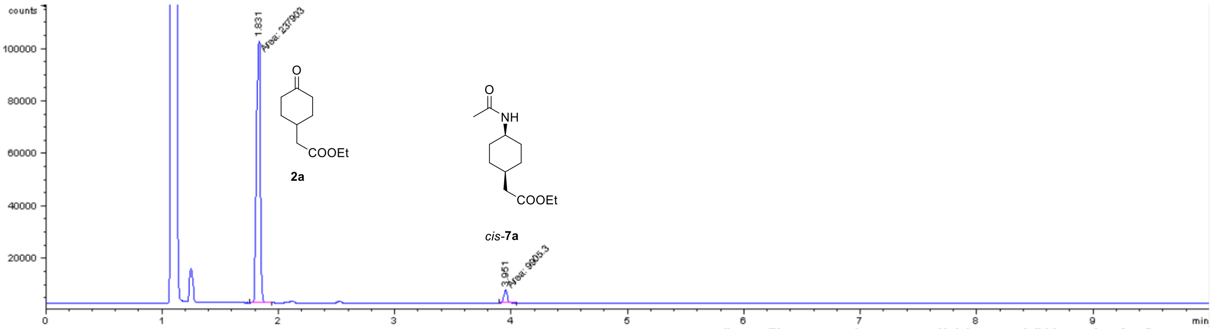

#	Time	Area	Height	Width	Area%	Symmetry
1	1.831	237902.9	100087.9	0.0396	96.003	1.814
2	3.951	9905.3	4918.2	0.0336	3.997	1.216

Figure GC1 GC chromatogram of amination of **2a** with immobilized whole-cell *CvS*-TA_{W60C} after Ac_2O derivatization (stationary conditions; HP-5; oven program: 180-210 °C 5 °C/min, 210 °C 1 min).

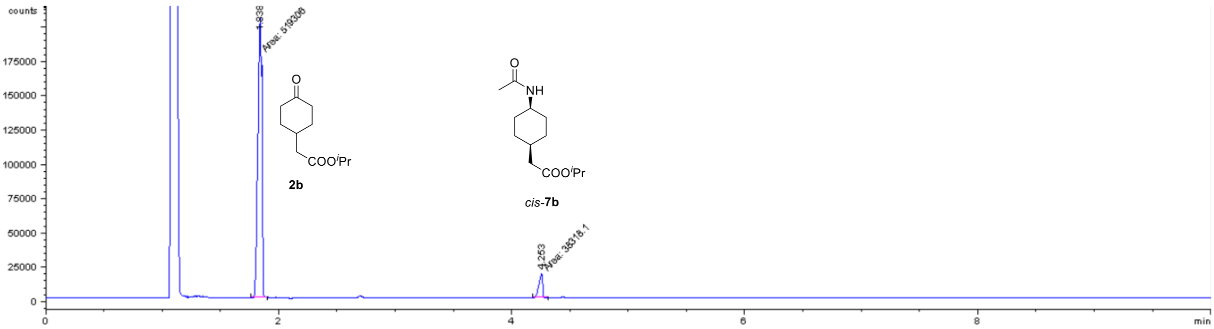

#	Time	Area	Height	Width	Area%	Symmetry
1	1.838	519305.8	204828.9	0.0423	93.128	1.179
2	4.253	38318.1	17628	0.0362	6.872	2.672

Figure GC2 GC chromatogram of amination of **2b** with immobilized whole-cell *CvS*-TA_{W60C} after Ac_2O derivatization (stationary conditions; HP-5; oven program: 180-210 °C 5 °C/min, 210 °C 1 min).

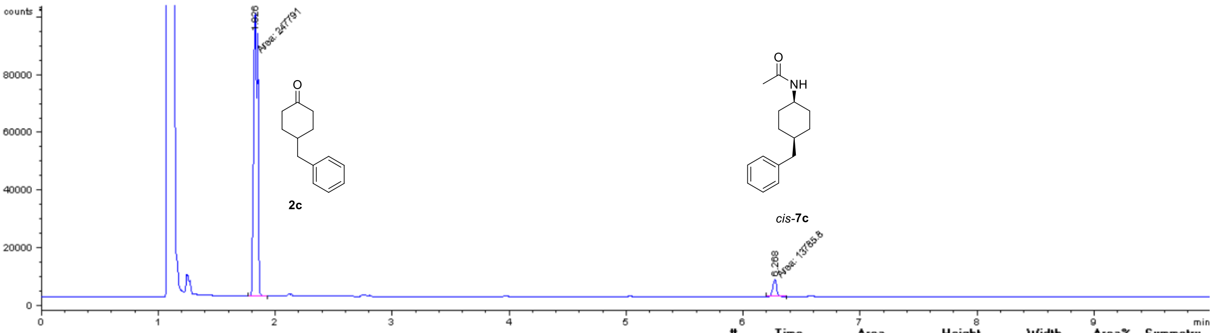

#	Time	Area	Height	Width	Area%	Symmetry
1	1.826	247790.5	98694.9	0.0418	94.730	0.674
2	6.268	13785.8	5826.7	0.0394	5.270	1.162

Figure GC3 GC chromatogram of amination of **2c** with immobilized whole-cell *CvS*-TA_{W60C} after Ac_2O derivatization (stationary conditions; HP-5; oven program: 180-210 °C 5 °C/min, 210 °C 1 min).

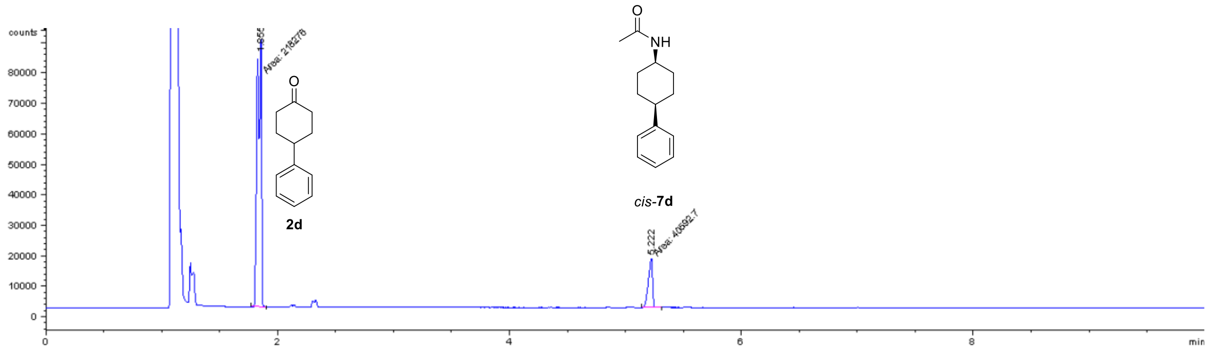

#	Time	Area	Height	Width	Area%	Symmetry
1	1.855	218276	93517.7	0.0389	84.319	5.584
2	5.222	40592.7	16227.6	0.0417	15.681	2.568

Figure GC4 GC chromatogram of amination of **2d** with immobilized whole-cell *CvS*-TA_{W60C} after Ac_2O derivatization (stationary conditions; HP-5; oven program: 180-210 °C 5 °C/min, 210 °C 1 min).

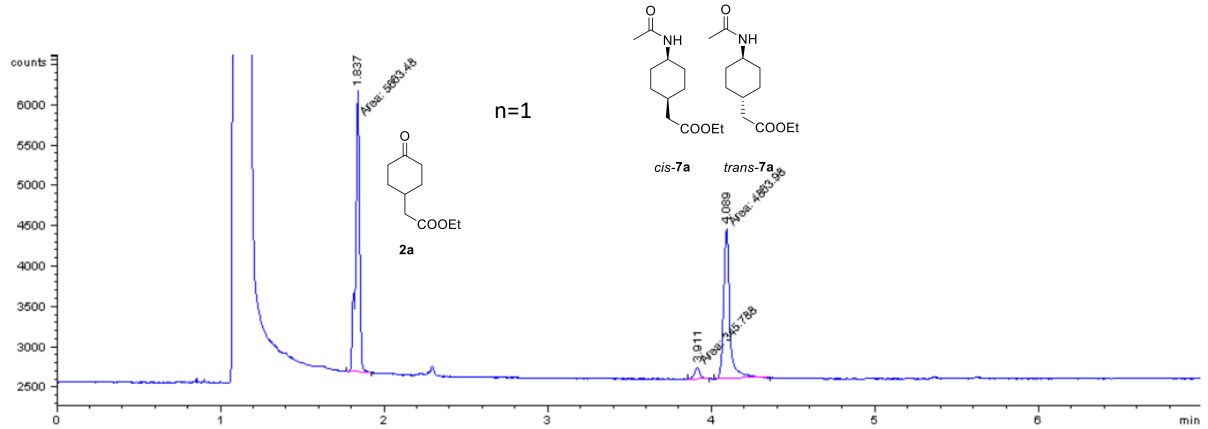

#	Time	Area	Height	Width	Area%	Symmetry
1	1.837	5663.5	3508.5	0.0269	52.086	1.386
2	3.911	345.8	149.2	0.0386	3.180	1.038
3	4.089	4864	1861.3	0.0436	44.733	0.839

Figure GC5 GC chromatogram of deamination of *cis*/*trans*-**1a** with immobilized *CvS*-TA_{W60C} in continuous-flow mode (n=1, stationary conditions; HP-5; oven program: 180-210 °C 5 °C/min, 210 °C 1 min).

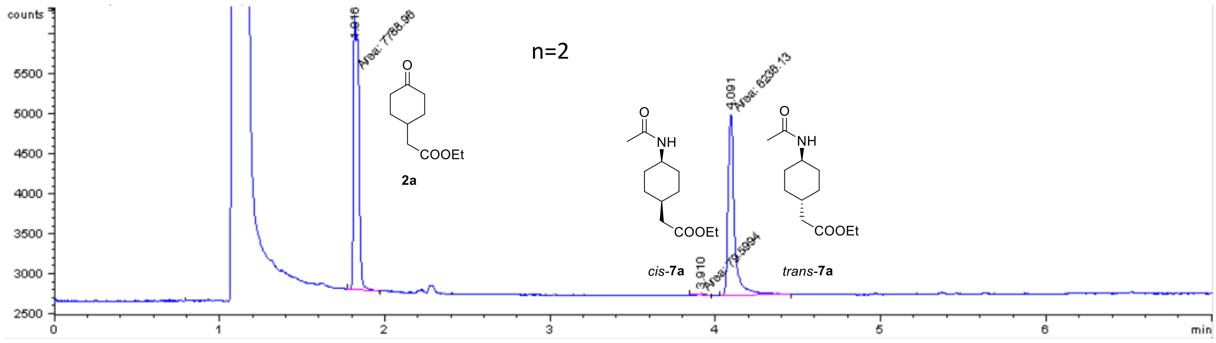

#	Time	Area	Height	Width	Area%	Symmetry
1	1.816	7789	3458.7	0.0375	55.222	0.345
2	3.91	79.6	29.9	0.0443	0.564	0.655
3	4.091	6236.1	2261.6	0.046	44.213	0.721

Figure GC6 GC chromatogram of deamination of *cis*/*trans*-**1a** with immobilized *CvS*-TA_{W60C} in continuous-flow mode (n=2, stationary conditions; HP-5; oven program: 180-210 °C 5 °C/min, 210 °C 1 min).

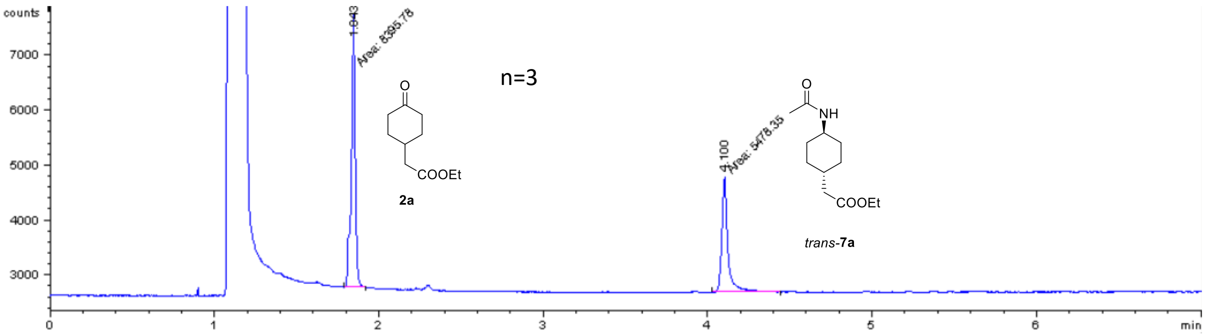

#	Time	Area	Height	Width	Area%	Symmetry
1	1.843	8395.8	4970.9	0.0282	60.514	1.246
2	4.1	5478.4	2098.6	0.0435	39.486	0.706

Figure GC7 GC chromatogram of deamination of *cis*/*trans*-**1a** with immobilized *CvS*-TA$_{W60C}$ in continuous-flow mode (n=3, stationary conditions; HP-5; oven program: 180-210 °C 5 °C/min, 210 °C 1 min).

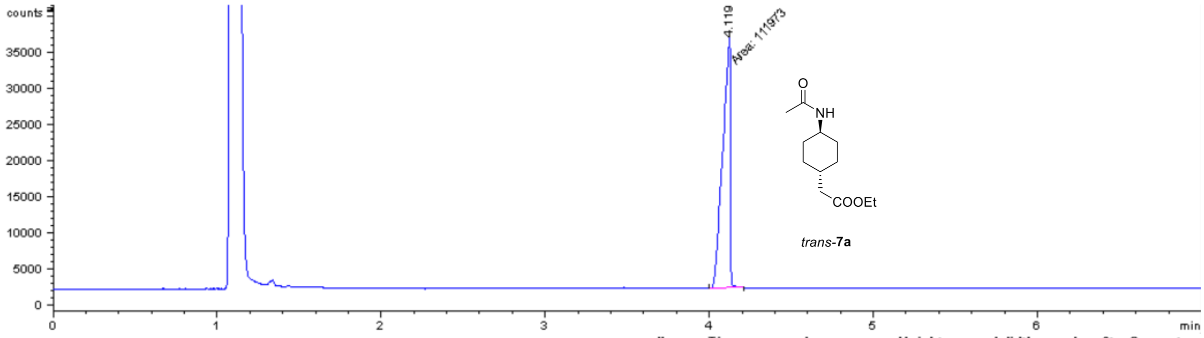

#	Time	Area	Height	Width	Area%	Symmetry
1	4.119	111973.2	35057.7	0.0532	100.000	5.706

Figure GC8 GC chromatogram of *trans*-**1a** after working of reaction mixture from continuous-flow experiment, formation of hydrochloride salt and derivatized (HP-5; oven program: 180-210 °C 5 °C/min, 210 °C 1 min).

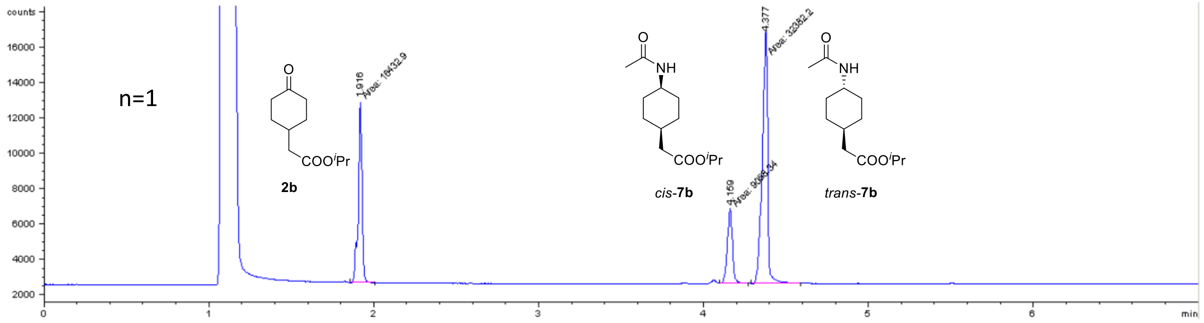

#	Time	Area	Height	Width	Area%	Symmetry
1	1.916	16432.9	10264.1	0.0267	28.395	1.256
2	4.159	9058.3	4234	0.0357	15.652	1.153
3	4.377	32382.2	14593.6	0.037	55.954	2.135

Figure GC9 GC chromatogram of deamination of *cis*/*trans*-**1b** with immobilized *CvS*-TA$_{W60C}$ in continuous-flow mode (n=1, stationary conditions; HP-5; oven program: 180-210 °C 5 °C/min, 210 °C 1 min).

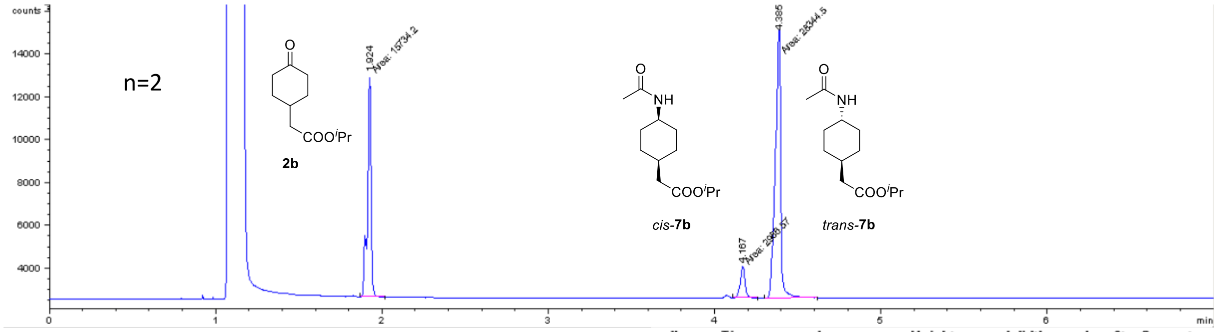

#	Time	Area	Height	Width	Area%	Symmetry
1	1.924	15734.2	10327.9	0.0254	33.431	1.446
2	4.167	2986.6	1475.4	0.0337	6.346	1.065
3	4.385	28344.5	12692.9	0.0372	60.224	1.902

Figure GC10 GC chromatogram of deamination of *cis*/*trans*-**1b** with immobilized *CvS*-TA$_{W60C}$ in continuous-flow mode (n=2, stationary conditions; HP-5; oven program: 180-210 °C 5 °C/min, 210 °C 1 min).

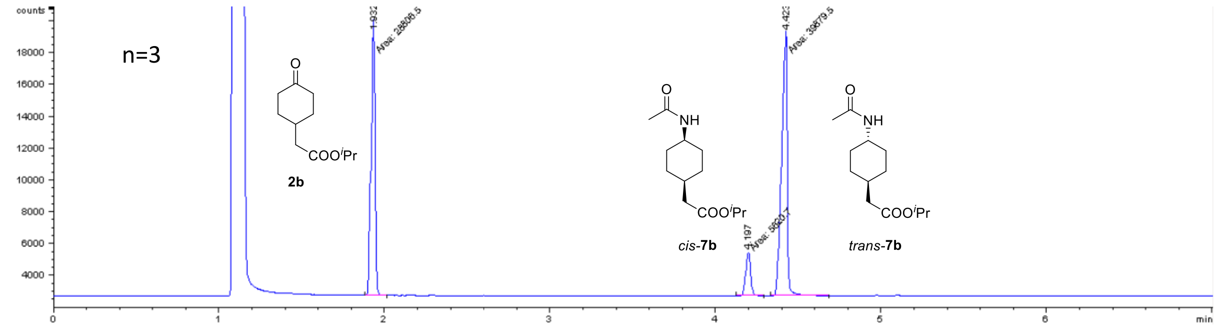

#	Time	Area	Height	Width	Area%	Symmetry
1	1.932	28806.5	17668.8	0.0272	38.767	1.463
2	4.197	5620.7	2708.8	0.0346	7.564	1.182
3	4.423	39879.5	16687.5	0.0398	53.669	2.547

Figure GC11 GC chromatogram of deamination of *cis*/*trans*-**1b** with immobilized *CvS*-TA$_{W60C}$ in continuous-flow mode (n=3, stationary conditions; HP-5; oven program: 180-210 °C 5 °C/min, 210 °C 1 min).

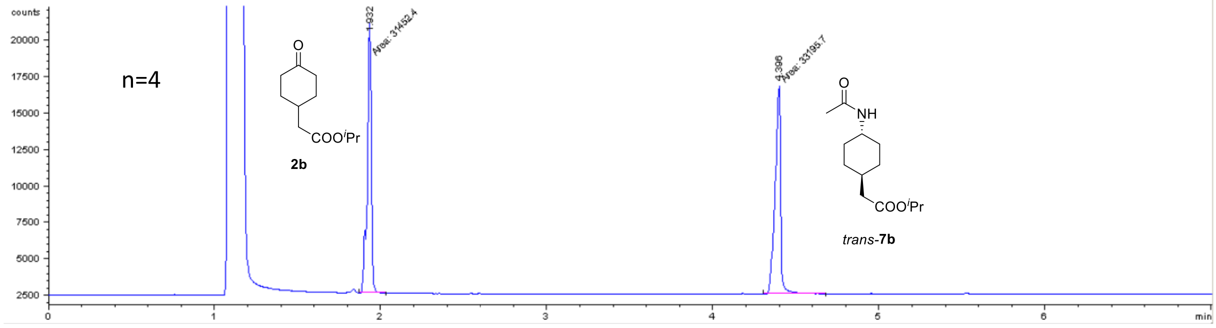

#	Time	Area	Height	Width	Area%	Symmetry
1	1.932	31452.4	18840.2	0.0278	48.652	1.462
2	4.396	33195.7	14299.6	0.0387	51.348	2.048

Figure GC12 GC chromatogram of deamination of *cis*/*trans*-**1b** with immobilized *CvS*-TA$_{W60C}$ in continuous-flow mode (n=4, stationary conditions; HP-5; oven program: 180-210 °C 5 °C/min, 210 °C 1 min).

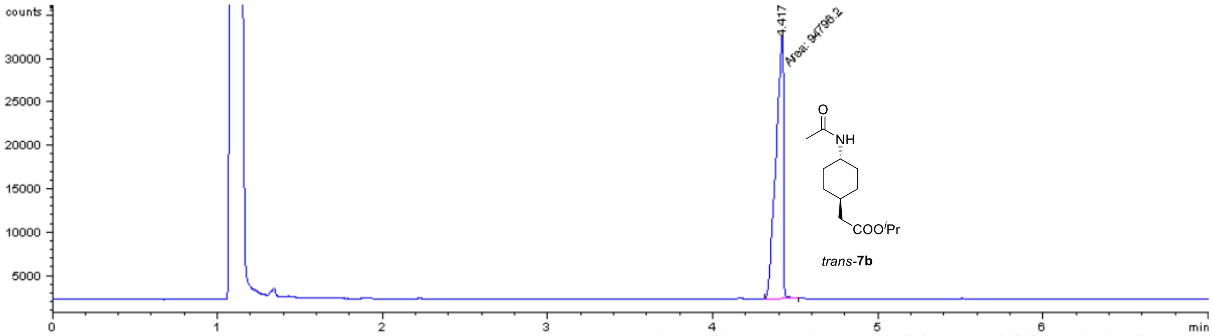

#	Time	Area	Height	Width	Area%	Symmetry
1	4.417	94796.2	30621	0.0516	100.000	4.909

Figure GC13 GC chromatogram of *trans*-**1b** after working of reaction mixture from continuous-flow experiment, formation of hydrochloride salt and derivatized (HP-5; oven program: 180-210 °C 5 °C/min, 210 °C 1 min).

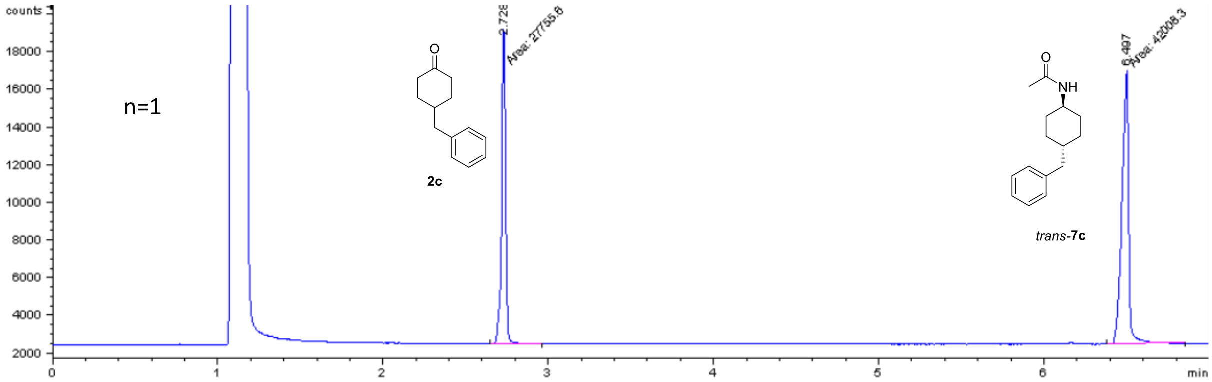

#	Time	Area	Height	Width	Area%	Symmetry
1	2.728	27755.6	16859.3	0.0274	39.785	1.186
2	6.497	42008.3	14549.8	0.0481	60.215	1.923

Figure GC14 GC chromatogram of deamination of *cis*/*trans*-**1c** with immobilized *CvS*-TA$_{W60C}$ in continuous-flow mode (n=1, stationary conditions; HP-5; oven program: 180-210 °C 5 °C/min, 210 °C 1 min).

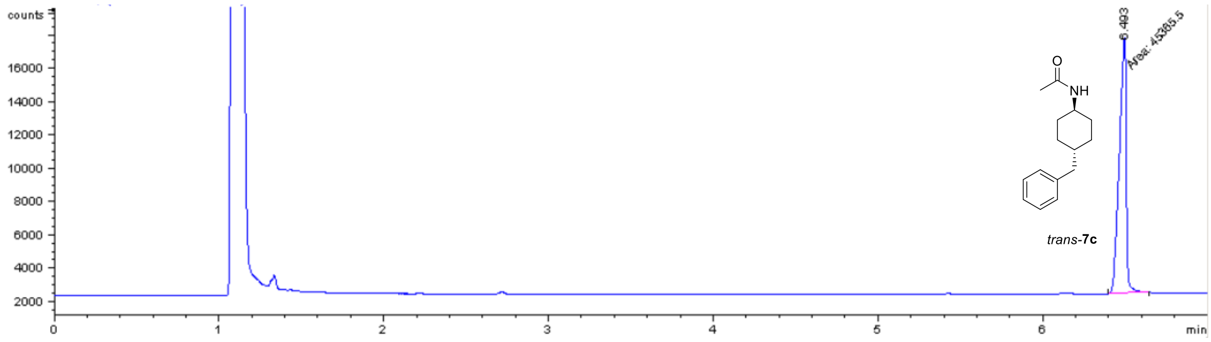

#	Time	Area	Height	Width	Area%	Symmetry
1	6.493	45365.5	15368.6	0.0492	100.000	2.351

Figure GC15 GC chromatogram of *trans*-**1c** after working of reaction mixture from continuous-flow experiment, formation of hydrochloride salt and derivatized (HP-5; oven program: 180-210 °C 5 °C/min, 210 °C 1 min).

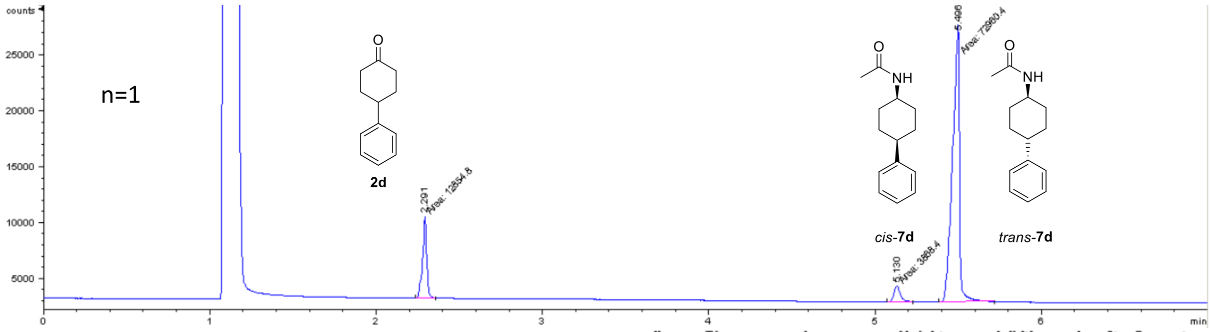

#	Time	Area	Height	Width	Area%	Symmetry
1	2.291	12854.8	7351.6	0.0291	14.334	1.233
2	5.13	3868.4	1423.1	0.0453	4.313	0.866
3	5.496	72960.4	24892.3	0.0489	81.353	2.96

Figure GC16 GC chromatogram of deamination of *cis*/*trans*-**1d** with immobilized *CvS*-TA$_{W60C}$ in continuous-flow mode (n=1, stationary conditions; HP-5; oven program: 180-210 °C 5 °C/min, 210 °C 1 min).

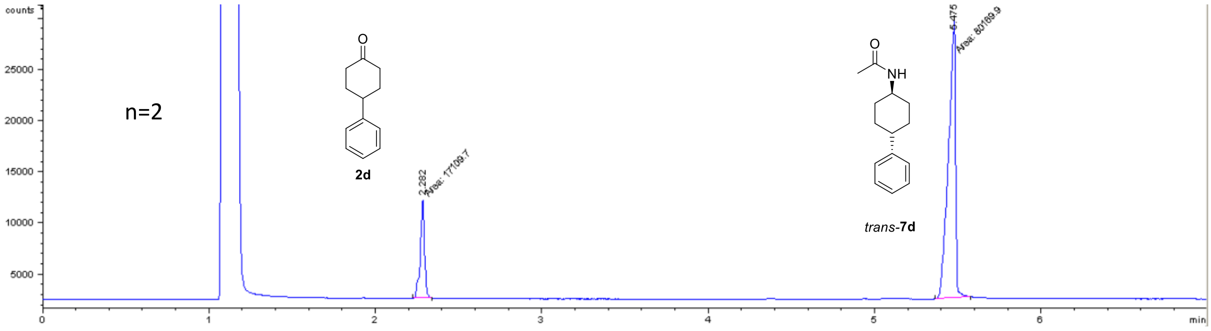

#	Time	Area	Height	Width	Area%	Symmetry
1	2.282	17109.7	9683	0.0294	17.588	1.229
2	5.475	80169.9	27351	0.0489	82.412	3.763

Figure GC17 GC chromatogram of deamination of *cis*/*trans*-**1d** with immobilized *CvS*-TA$_{W60C}$ in continuous-flow mode (n=2, stationary conditions; HP-5; oven program: 180-210 °C 5 °C/min, 210 °C 1 min).

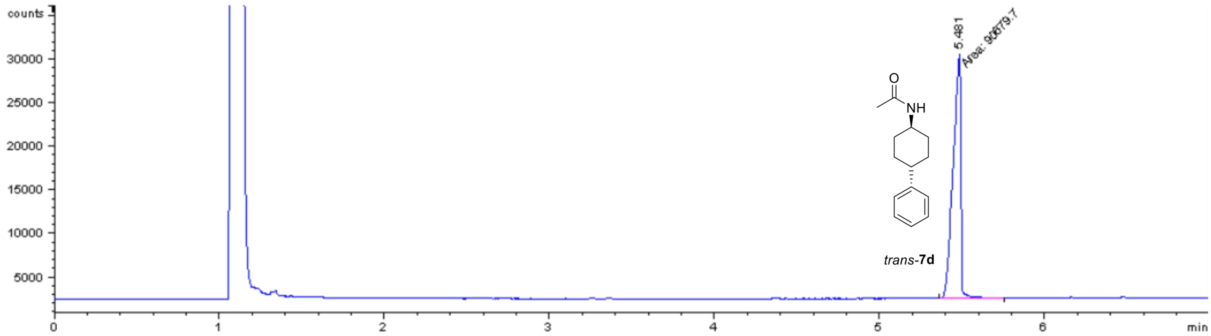

#	Time	Area	Height	Width	Area%	Symmetry
1	5.481	90679.7	28065.7	0.0538	100.000	3.865

Figure GC18 GC chromatogram of *trans*-**1d** after working of reaction mixture from continuous-flow experiment, formation of hydrochloride salt and derivatized (HP-5; oven program: 180-210 °C 5 °C/min, 210 °C 1 min).
