# Supplementary material for: Transaminase-catalysis to produce trans-4-substituted cyclohexane-1-amines including a key intermediate towards cariprazine
Source: Commun Chem. 2024 Apr 18;7:86. doi: 10.1038/s42004-024-01148-9 (PMC11026398; doi:10.1038/s42004-024-01148-9)
Supplement: Supplementary file 6 — Supplementary Data 3 [file 42004_2024_1148_MOESM6_ESM.pdf]

The IR spectra of synthesized substrates and intermediates

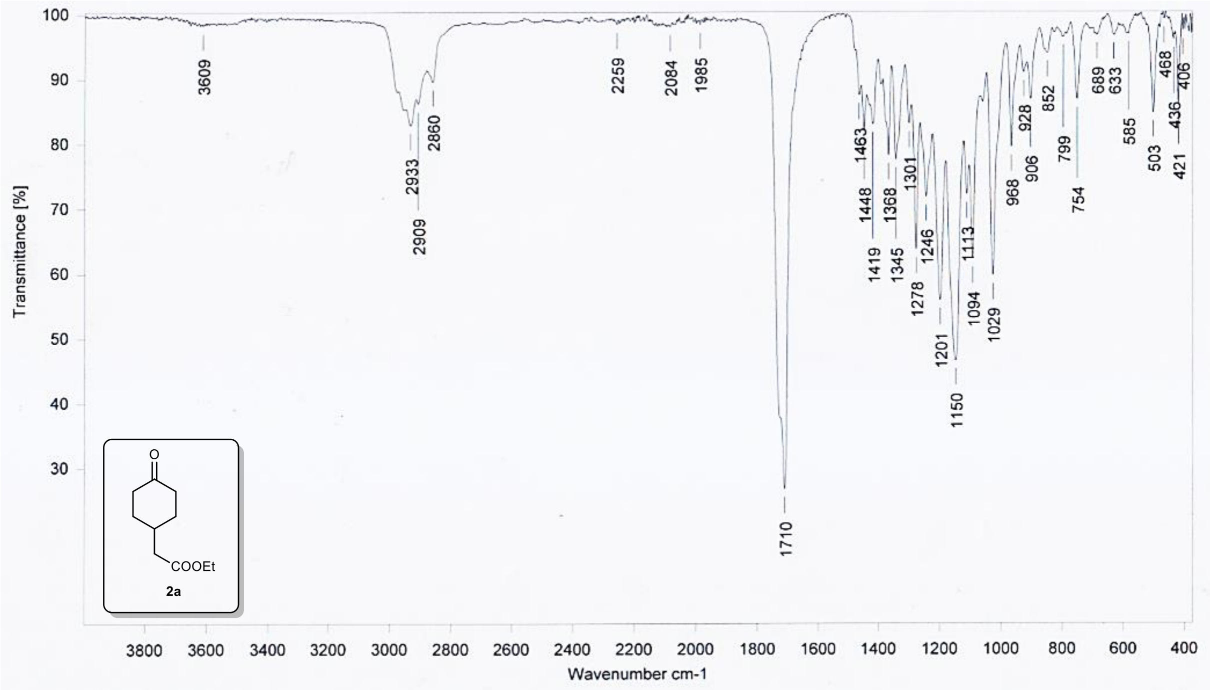

Figure IR1 FT-IR spectrum of ethyl-2-(4-oxocyclohexyl)acetate **2a**

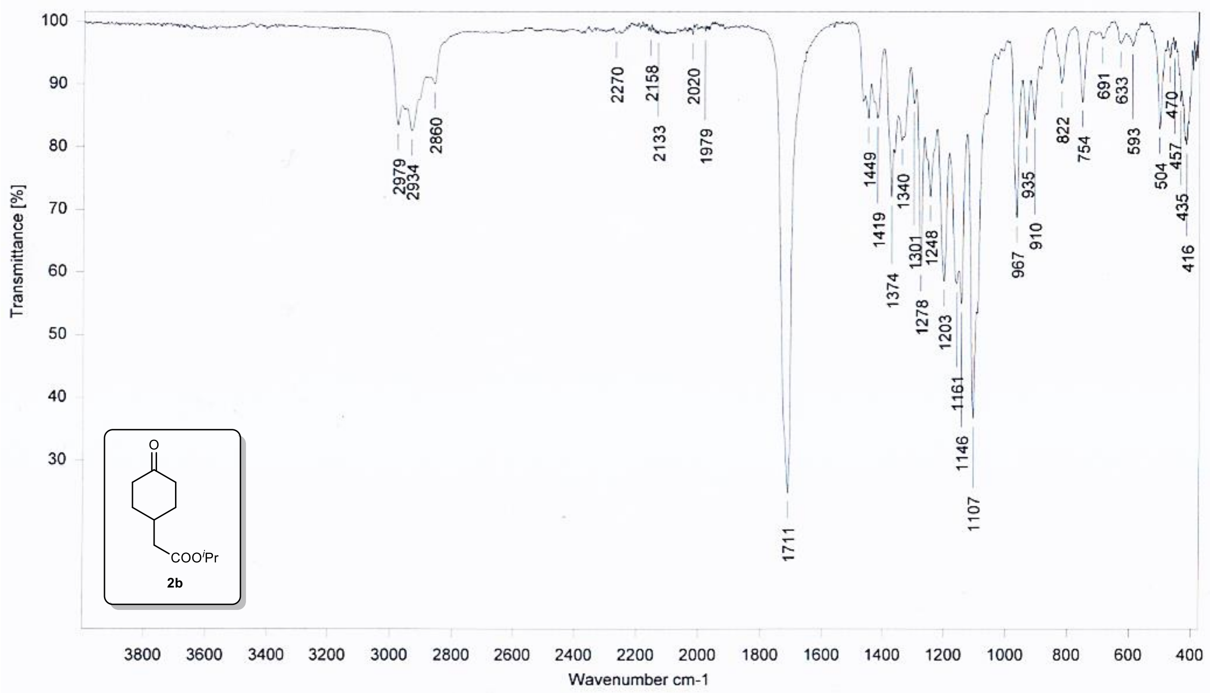

Figure IR2 FT-IR spectrum of isopropyl-2-(4-oxocyclohexyl)acetate **2b.**

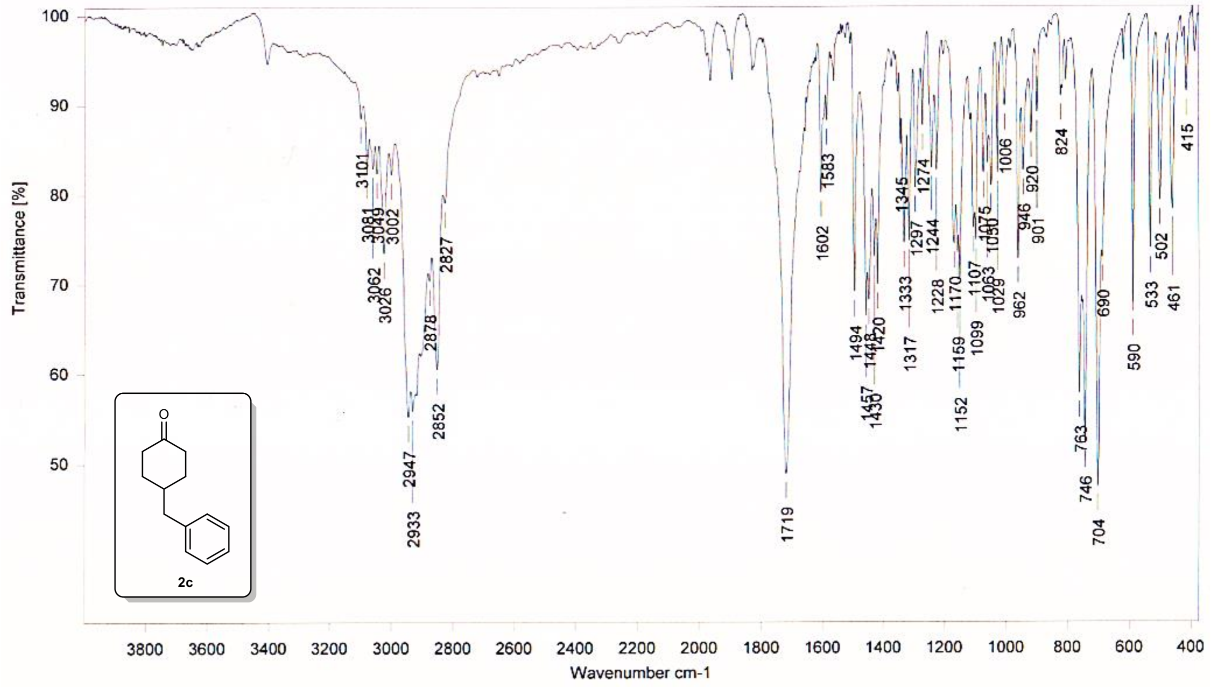

Figure IR3 FT-IR spectrum of 4-benzylcyclohexyl-1-one **2c.**

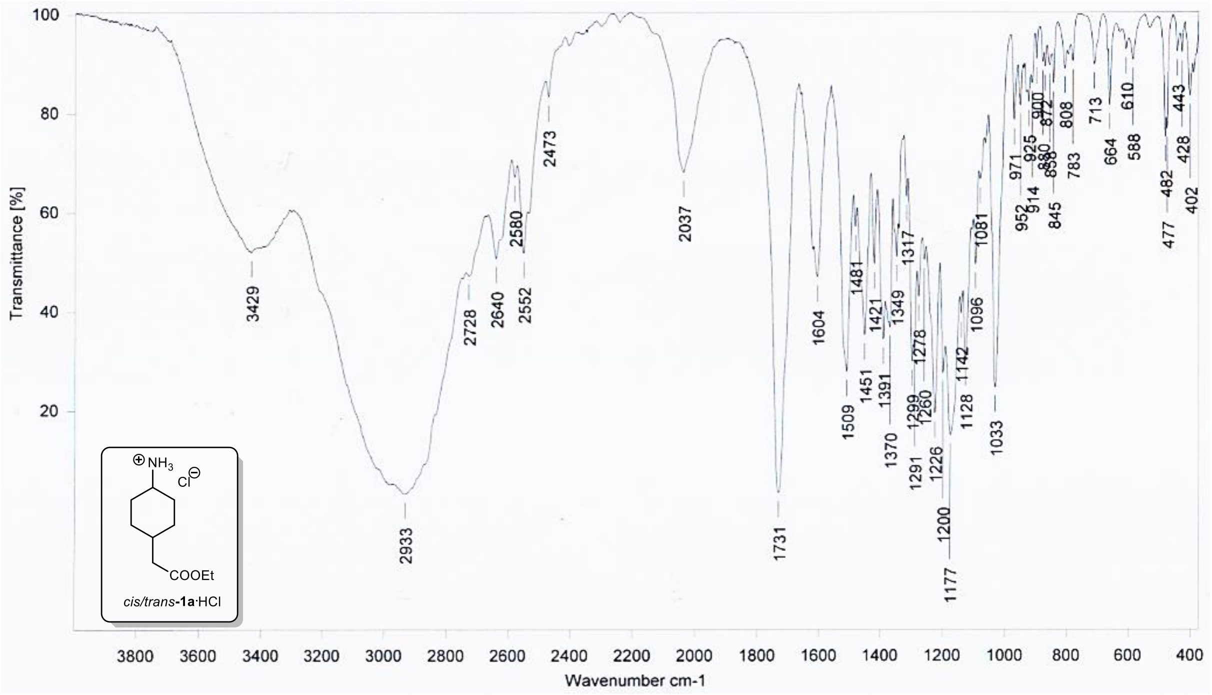

Figure IR4 FT-IR spectrum of *cis/trans*-4-(2-ethoxy-2-oxoethyl)cyclohexan-1-aminium chloride (*cis/trans*-**1a·**HCl)

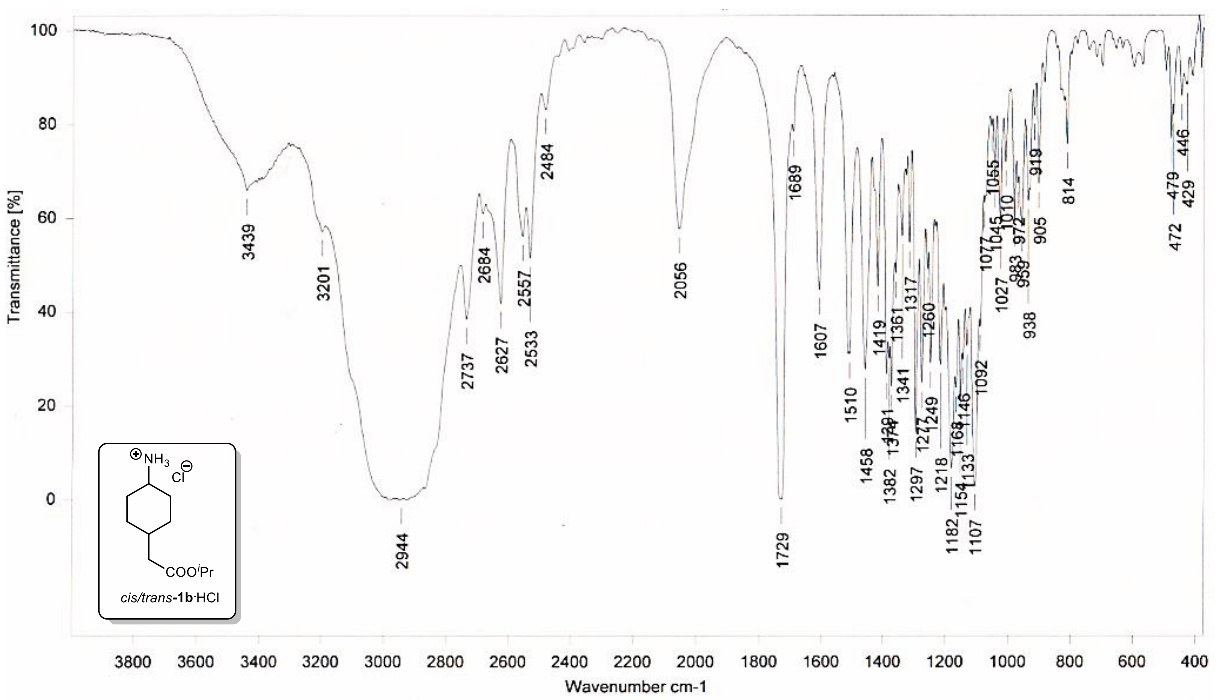

Figure IR5 FT-IR spectrum of *cis/trans*-4-(2-isopropoxy-2-oxoethyl)cyclohexan-1-aminium chloride (*cis/trans*-**1b**·HCl).

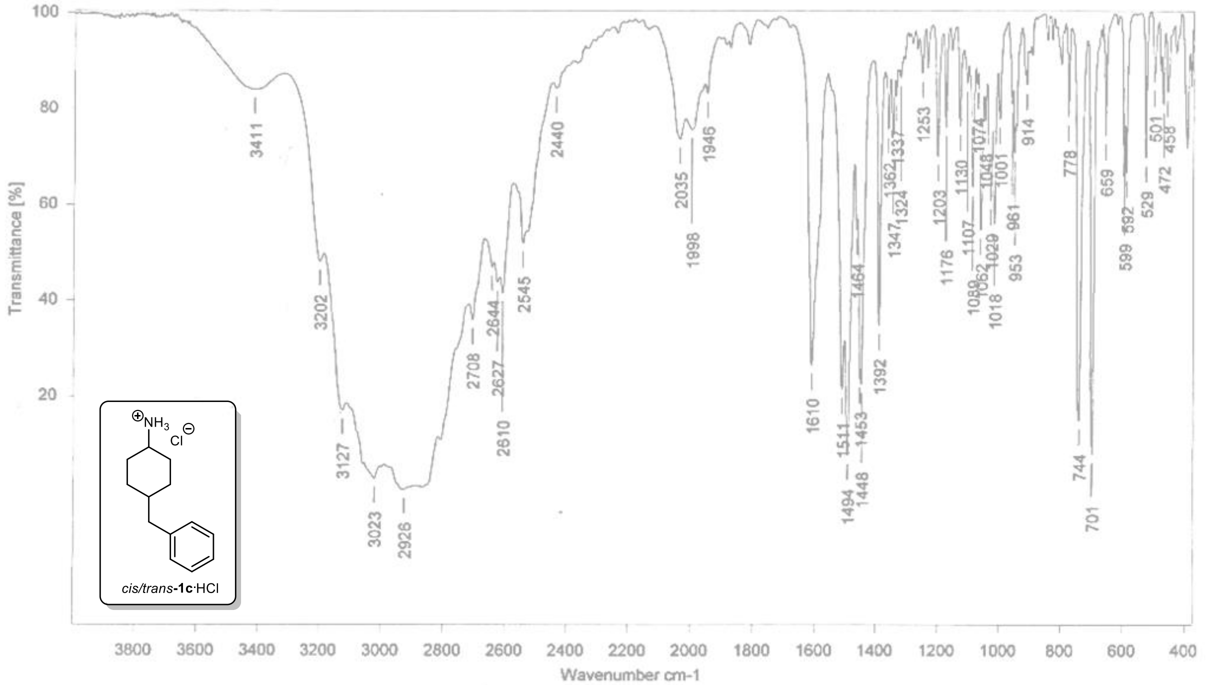

Figure IR6 FT-IR spectrum of *cis/trans*-4-benzylcyclohexan-1-aminium chloride (*cis/trans*-**1c**·HCl)

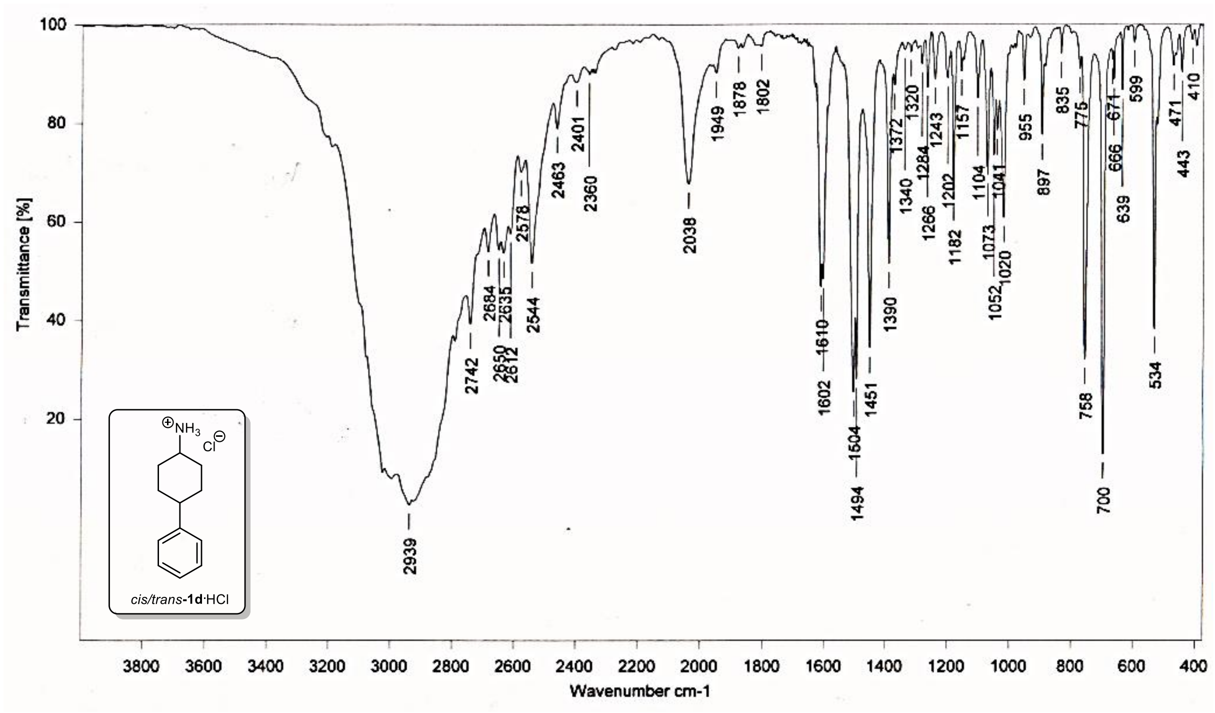

Figure IR7 FT-IR spectrum of *cis/trans*-4-phenylcyclohexan-1-aminium chloride (*cis/trans*-**1d**·HCl)
